# Potent inhibition of miR-27a by neomycin–bisbenzimidazole conjugates†
†Electronic supplementary information (ESI) available: Experimental details and synthesis of compounds. See DOI: 10.1039/c5sc01969a


**DOI:** 10.1039/c5sc01969a

**Published:** 2015-07-09

**Authors:** Smita Nahar, Nihar Ranjan, Arjun Ray, Dev P. Arya, Souvik Maiti

**Affiliations:** a Academy of Scientific and Innovative Research (AcSIR) , Anusandhan Bhawan, 2 Rafi Marg , New Delhi-110001 , India; b CSIR-Institute of Genomics and Integrative Biology , Mathura Road , Delhi-110020 , India . Email: souvik@igib.res.in ; Fax: +91-11-2766-7471 ; Tel: +91-11-2766-6156; c Department of Chemistry , Clemson University , Clemson , SC 29634 , USA; d CSIR-National Chemical Laboratory , Dr. Homi Bhabha Road , Pune , 411008 , India . Email: souvik@igib.res.in ; Email: dparya@clemson.edu

## Abstract

miRNAs are important components of regulatory networks that control gene expression and have implications in various diseases including cancer. Targeting oncogenic miRNAs with small molecules is currently being explored to develop cancer therapeutics. Here, we report the development of dual binding neomycin–bisbenzimidazole conjugates that target oncogenic miR-27a with high affinity (*K*_a_ = 1.2 to 7.4 × 10^8^ M^–1^). These conjugates bring significant reduction (∼65% at 5 μM) in mature miRNA levels and penetrate easily in the cells where they localise both in the cytoplasm and the nucleus. Cell cycle analysis showed significant increase in the G0/G1 phase (∼15%) and decrease in the S phase (∼7%) upon treatment with neomycin–bisbenzimidazole conjugates, suggesting inhibition of cell proliferation. Using the conjugation approach, we show that moderately binding ligands can be covalently combined into high affinity binders. This study also highlights the role of linker optimization in designing high affinity ligands for miR-27a targeting.

## Abstract 2

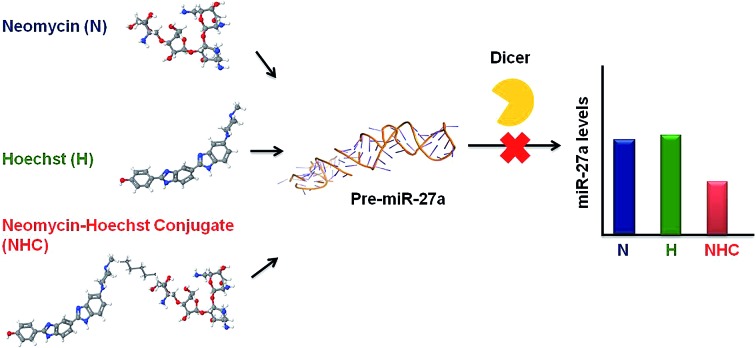
Potent downregulation of oncogenic miRNA is obtained by conjugation of neomycin and bisbenzimidazoles.

## Introduction

miRNAs are ubiquitously expressed conserved class of small non-coding RNAs that regulate gene expression post transcriptionally.^1^ Most miRNAs are derived as long primary transcripts (pri-miRNA) transcribed by RNA Pol-II from their genomic location. The canonical processing of the transcript starts when the nuclear microprocessor complex comprising Drosha (RNAse III enzyme) and its cofactor DGCR8, recognize the hairpin structure in pri-miRNA, cleaving it to a ∼70 nucleotide precursor form (pre-miRNA). This pre-miRNA is subsequently transported by Exportin-5/Ran-GTP to the cytosol where it is subsequently processed by Dicer to generate a ∼22 nucleotide mature miRNA duplex. One of the strands of the duplex is incorporated into the RISC complex containing Argonaute (Ago) proteins. miRNAs guide the RISC complex to 3′ UTR of its cognate mRNA and induce degradation or translation repression based on the extent of Watson–Crick base pairing.^2^ Over one-third of the human genome is predicted to be targeted by miRNAs, having a profound impact on the human proteome.^3^,^4^ The involvement of miRNAs in almost all crucial biological processes such as development, apoptosis, cell differentiation and cell proliferation have highlighted the importance of these tiny regulators.^5^ Deregulation of miRNA expression has been linked with disease onset and progression ranging from cardiovascular diseases to cancer.^6^ miRNAs that promote cellular proliferation and/or repress programmed cell death and are amplified in cancers act as oncomiRs. On the other hand, tumor suppressive miRNAs having regulatory functions to induce apoptosis and/or limit cancer growth are downregulated in various cancers.^7^ Thus, from a therapeutic perspective, restoring the imbalance in miRNA expression levels is imperative. Various loss-of-function studies based on conventional sequence specific antisense inhibition of upregulated miRNAs have been investigated in recent years.^8^ Moreover, an array of chemical modifications in oligonucleotides including phosphorothioates, 2′-*O*-alkyl derivatives such as 2′-*O*-methoxyethyl (MOE),^9^ 2′-OMe,^10^,^11^ 2′-F,^12^ peptide nucleic acids (PNAs)^13^ and locked nucleic acids (LNAs)^14^,^15^ have aimed at improving the stability and affinity of these oligonucleotides. However, major challenges like targeted intracellular delivery, insufficient cellular uptake, poor PK/PD properties and inconvenient scalability restrain their use as promising drugs. Small molecules, on the other hand endeavour to provide an alternative and unconventional approach to target secondary structures embedded in precursor miRNA forms, having added advantages of better cell permeability, ideal PK/PD properties and higher biostability.^16^ The complex secondary structure of pre-miRNA, where hairpin stem loops and bulges are acquiescent to selective ligand binding, makes it an apt candidate for drug invention. Numerous reports have corroborated the proof of concept for small molecule targeting pre-miRNAs, emphasizing their therapeutic utility. These include diazobenzene as inhibitor of pri-miR-21 formation,^17^ sulphonamide targeting miR-122 in liver,^18^ streptomycin hindering pre-miR-21 processing,^19^ a highly selective benzimidazole molecule targeting pre-miR-96 20 and enoxacin and quinazoline compounds universally upregulating miRNAs.^21^,^22^ Thus, discovery of small molecule lead structures that can selectively target and inhibit oncomiRs has wide therapeutic implications.

**Scheme 1:**
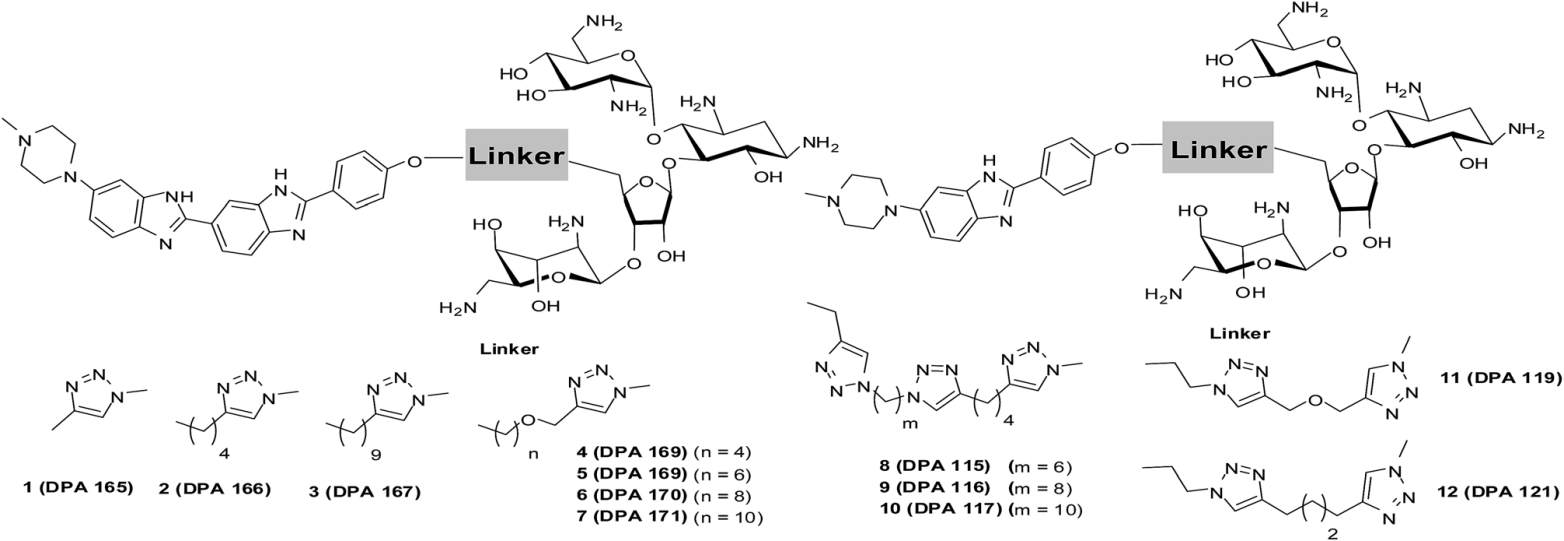
Structures of neomycin–mono/bisbenzimidazoles used in this study (see ESI† for complete list of structures used in this study).

Abnormal upregulation of miR-27a, an oncomiR is known to promote tumor growth and metastasis.^23^–^26^ Thus, downregulation of miR-27a is of therapeutic importance. One of the preceding screens in our lab established neomycin, an aminoglycoside to target the pre-miR-27a terminal loop. This targeting hindered Dicer processing thus repressing mature miR-27a levels.^27^ However, it is known that aminoglycosides are poorly taken up by eukaryotic cells,^28^ and thus, the effective concentration of neomycin for inhibiting miR-27a used was 20 μM. Neomycin and its conjugates have also been found to target other RNA structures and A-form nucleic acids^29^,^30^ including the trans-activating region (TAR) of the HIV virus.^31^,^32^ In order to increase the affinity and selectivity of neomycin against our target sequence miR-27a, we contemplated a synergistic approach which involves covalent linkage of two RNA binding ligands to improve the targeting of pre-miR-27a. We chose the bisbenzimidazole scaffold as the second ligand because these are a highly cell permeable, important class of bioactive, heterocyclic aromatic compounds. Moreover, they are also known to bind to other RNA structures, such as a specific uridine bulge in TAR RNA, deletion of which abolishes binding.^33^ Recently, it was shown that the bisbenzimidazole core structure has the ability to selectively target internal loop structures in RNA.^20^,^34^ We have also shown that the conjugation of neomycin to a bisbenzimidazole (Hoechst 33258) results in enhanced binding to an RNA duplex.^35^ This propelled us to conjugate a known antibiotic, neomycin, to the cell permeable mono and bisbenzimidazoles derived from Hoechst 33258 with varying linker lengths and compositions to evaluate their effectiveness in modulating miR-27a levels.^36^ (Scheme 1, Fig. S1 and S2 in ESI†).

## Results and discussion

**Scheme 2:**
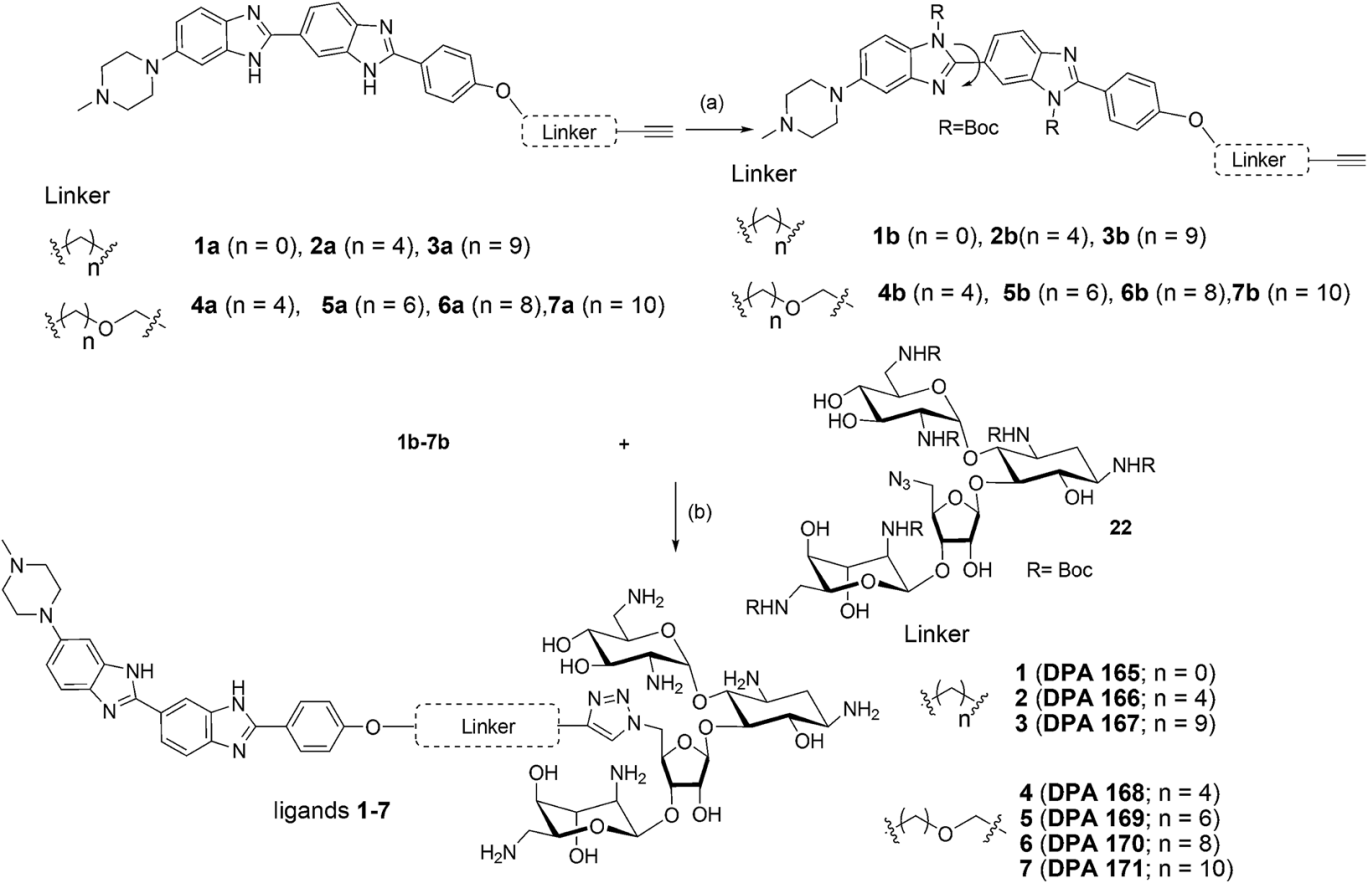
(a) NaH, THF, (Boc)_2_O, rt, ∼51 to 87% overall yield. (b) (i) CuSO_4_, sodium ascorbate, H_2_O, C_2_H_5_OH, rt, 24–30 h; (ii) trifluoroacetic acid, dichloromethane, rt, 3 h (29–48% yield for steps i and ii).

The synthesis of neomycin–bisbenzimidazole conjugates (**1–7**) used in this study was achieved using click-chemistry-based conjugation of terminal alkyne modified Hoechst 33258 derivatives^37^ with Boc-protected neomycin azide^38^ followed by deprotection of the protecting groups. As shown in Scheme 2, the alkyne modified Hoechst 33258 derivatives **1a–7a**^37^ were Boc protected using di-tertiary butyl dicarbonate. This led to the formation of two diBoc-protected rotamers of **1b–7b** which can be separated by column chromatography. The conversion of terminal alkyne modified Hoechst 33258 derivatives **1a–7a** to their corresponding diBoc-protected analogues increases their reactivity towards click reactions under the conditions described in Scheme 2 and also allows for their much easier purification using silica gel column chromatography. The diBoc-protected derivatives **1b–7b** were then reacted with Boc protected neomycin azide (**22**) which led to the formation of Boc protected conjugates of **1–7**. The Boc protecting groups were then removed using trifluoroacetic acid to give the desired conjugates **1–7**. The conjugates were then characterized by spectroscopic methods (NMR, mass spectrometry) and their purity was checked by HPLC which was >95%. In a similar click-chemistry-based conjugation strategy, compounds **8–12** were synthesized; their complete synthesis details are provided in the ESI, Schemes S1–S3.†

**Fig. 1:**
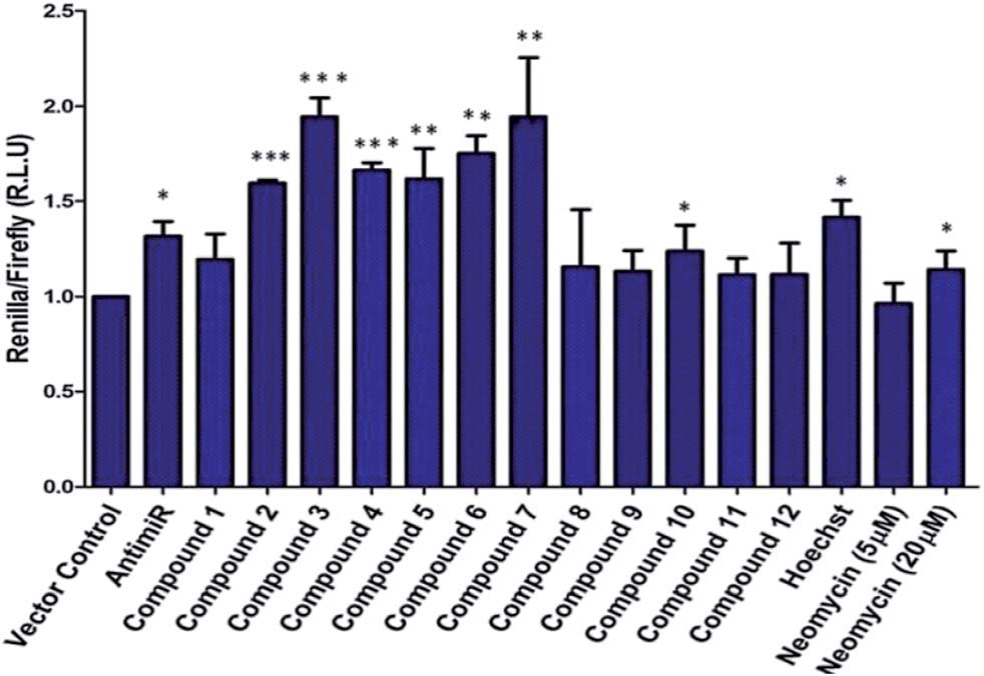
Effect of neomycin-mono/bisbenzimidazole conjugates on prohibitin luciferase signal at a final concentration of 5 μM. Anti-miR-27a (with 5 LNA modifications) at a concentration of 100 nM was used as a positive control. Compounds **2–7** showed substantial increase in luciferase intensity whereas compounds **8–12** did not show any significant effect. Neomycin at 5 μM did not show any effect but exhibited upregulation at 20 μM. Native compound, Hoechst 33258 treated at 5 μM showed slight increase in luciferase intensity. All of the *Renilla* luciferase data were normalized with firefly intensity and data is compared with vector control. Error bars represent ±SD, calculated from three independent experiments. *p < 0.05, **p < 0.01, ***p < 0.001 (Student's t-test).

We examined the cytotoxic potential of each neomycin–mono/bisbenzimidazole conjugate (**1–12**) in order to rule out the direct cytotoxic effects of conjugation. We observed that MCF-7 cells remained viable and the compounds showed no significant toxicity after 24 h compared to the untreated cells (Fig. S3†). The parent compounds neomycin and Hoechst 33258 also did not show a cytotoxic response, as evaluated by MTT assay. This implied that there is no effect of the linkers of varying length and composition on cytotoxicity. Next, we employed a luciferase-based screening strategy to screen twelve water soluble neomycin–mono/bisbenzimidazole conjugates (**1–12**) for their ability to modulate miR-27a levels in the MCF-7 breast cancer cell line. A dual luciferase construct harbouring a 3′ UTR of the miR-27a target site, prohibitin (PHB), was cloned downstream of *Renilla* luciferase gene.^39^ Firefly luciferase gene was used as a normalising control. MCF-7 cells overexpressing miR-27a would lead to decrease in *Renilla* luciferase intensity due to the repressive nature of miR-27a towards its target prohibitin in most cellular contexts. This reporter system could be exploited for screening of potential small molecule inhibitors that could reverse the decrease in the luciferase signal. We reasoned that conjugation of neomycin to Hoechst 33258 might enhance its cell penetrating properties, thus we screened the twelve conjugates independently at a lower dosage of 5 μM for their ability to enhance the *Renilla* luciferase signal (Fig. 1). An antimiR-27a (with 5 LNA modifications) was transfected at 100 nM as positive control to ensure that the assay is working optimally. Compounds **2–7** showed greater than 1.5 to 2 fold enhancement in PHB levels, thus showing higher potency than the antimiR in downregulating miR-27a. Hoechst 33258 administered at 5 μM also showed slightly elevated levels of PHB luciferase signal.

**Fig. 2:**
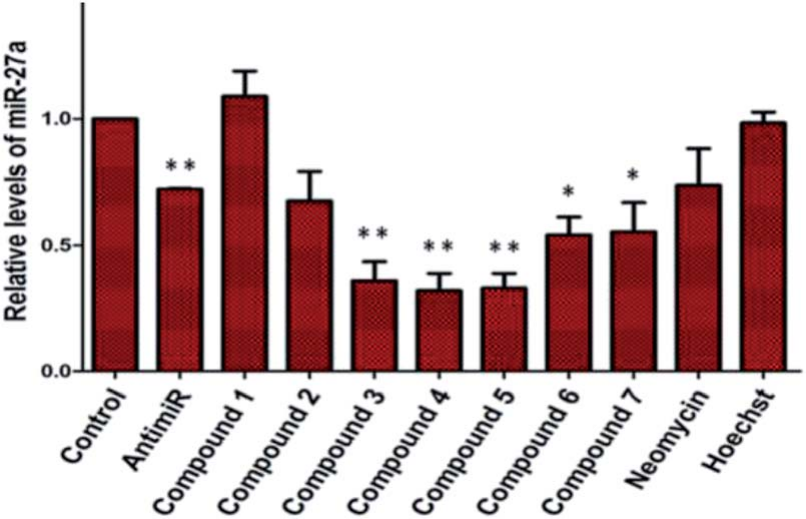
qPCR analysis to determine the expression of miR-27a post treatment of neomycin–bisbenzimidazole conjugates (**1–7**). Compounds **3–5**, showed significant decrease in mature miR-27a levels as compared to untreated control. Five LNA modified anti-miR-27a (100 nM) showed ∼30% reduction in miR-27a levels. Compound **1**, Hoechst 33258 and neomycin (at 5 μM concentration) did not show any significant effect on levels of mature miR-27a. Error bars represent ±SD, calculated from three independent experiments. *p < 0.05; **p < 0.01 (Student's t-test).

A control experiment with unconjugated neomycin at 5 μM did not show any significant effect on luciferase intensity, however, it showed an increase in R.L.U when treated at 20 μM. Thus, conjugation of Hoechst-33258-derived bisbenzimidazole units to neomycin enhances their potency at low dosage. For further validation we selected compounds **2–7**, which produced most significant effect on PHB levels (higher than Hoechst 33258 alone). Compound **1** (having a short linker) did not show a significant effect on PHB levels and was taken forward as a negative control. The extent and specificity of these potential inhibitors (conjugates **2–7**) in down-regulating miR-27a was further examined by real-time qPCR. MCF-7 cells were each treated with conjugates **1–7** and expression of miR-27a was assessed after 48 h in comparison with the untreated control by qPCR (Fig. 2).

Most potent inhibitors of miR-27a levels were compounds **3**, **4** and **5** with ∼65% reduction in mature miRNA levels at 5 μM dosage, whereas parent compounds neomycin and Hoechst 33258 did not show a significant effect on the levels of miR-27a at 5 μM. Compounds **6** and **7** also showed significant inhibition in miR-27a levels of ∼55%.

**Fig. 3:**
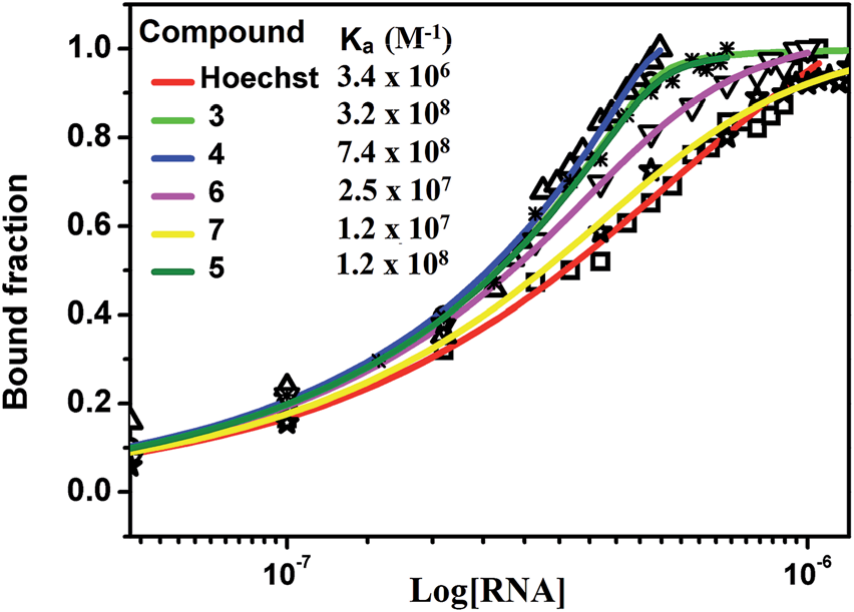
Fluorescence binding isotherm for conjugates **3–7** and Hoechst 33258 titrated with pre-miR-27a.

The different degree of variation in repressing miR-27a levels arises due to difference in the linker length, flexibility and composition as they change in aliphatic content and number of oxygen atoms. Next, to rule out the possibility of neomycin–bisbenzimidazole conjugates eliciting an indirect effect on miR-27a downregulation, we monitored direct interaction between pre-miR-27a and compounds **3–7** by fluorescence titration. At a fixed concentration (500 nM) of each of the compounds **3–7**, titration was performed with purified pre-miR-27a monitoring the changes in the fluorescence emission of the bisbenzimidazoles upon binding. We observed an increase in fluorescence upon incremental addition of pre-miR-27a until the signal reached saturation. The emission changes were used to construct a binding isotherm representing a fraction of the ligand bound (*α* fraction) plotted as a function of pre-miR-27a concentration (Fig. 3). The equilibrium binding affinity, *K*_a_ was calculated after fitting the binding isotherms for compounds **3–7** and Hoechst 33258 (assuming a 1 : 1 stoichiometric model of binding). Compounds **3**, **4** and **5** displayed strong equilibrium association constants (*K*_a_ = 3.2 × 10^8^ M^–1^, 7.4 × 10^8^ M^–1^ and 1.2 × 10^8^ M^–1^) showing enhancement in association constants by nearly two orders of magnitude when compared to Hoechst 33258 binding (*K*_a_ = 3.4 × 10^6^ M^–1^). The *K*_a_ for neomycin could not be obtained due to lack of intrinsic fluorescence.

Neomycin binds near the terminal loop of pre-miR-27a, as established in our previous study,^27^ so we wanted to determine the possible binding domain of neomycin–bisbenzimidazole conjugates. Thus, an *in-silico* approach was used to predict and study the binding pose of neomycin–bisbenzimidazole conjugates to the miRNA. The first step involved the prediction of the secondary and tertiary structure of the pre-miR using the MC-Fold|MC-Sym pipeline. The energy-minimized three-dimensional model was obtained (Fig. S4a, ESI†) using the protocol mentioned above (see the Experimental section for details). The compounds were built using ChemDraw, followed by OpenBabel and finally were minimized using Maestro9.8. To study the docking poses of neomycin–bisbenzimidazole conjugates, we performed molecular docking using AutoDock.^40^ The results from the docking experiment are given in Table 1. The best-docked position for each molecule (the selection criteria is mentioned in the Experimental section) was chosen for an all-atomistic molecular dynamic simulation for 100 ns to check for conformational stability using GROMACS 4.6.1.

**Table 1:** Estimated binding parameters for compounds **3–7** calculated using AutoDock

Compound	**3**	**4**	**5**	**6**	**7**
Estimated binding energy/kcal mol^–1^	–9.34	–7.06	–7.22	–8.44	–11.31
Estimated inhibition constant	142.90 nM	6.64 μM	645.27 nM	645.27 nM	5.14 nM
Electrostatic energy/kcal mol^–1^	–11.92	–10.87	–12.10	–12.10	–14.04
Ligand efficiency	–0.11	–0.08	–0.10	–0.10	–0.13
Number of hydrogen bonds	2	2	1	1	5

The simulations revealed that, though the compound **7** showed a high estimated binding energy with the miRNA, there was destabilization of the complex (Fig. S5 and S6, ESI†). The rest of the ligand–miRNA docked complexes showed stability across the simulation time. The docking results also display a partly stacked bisbenzimidazole unit (next to the piperazine moiety) between the base pairs of nucleotides 54 and 55 (Fig. S4b, ESI†). Anchored by neomycin in the major groove adjacent to the hairpin loop region, the bisbenzimidazole unit traverses through the groove curvature and undergoes bond rotation between the benzimidazole units before making stacking interactions. These results are in agreement with experimental studies using linear dichroism which have suggested intercalative binding of the bisbenzimidazole units of the same conjugates to a polymeric RNA duplex poly (rA)·r(U) (Ranjan, Arya, unpublished results).

**Fig. 4:**
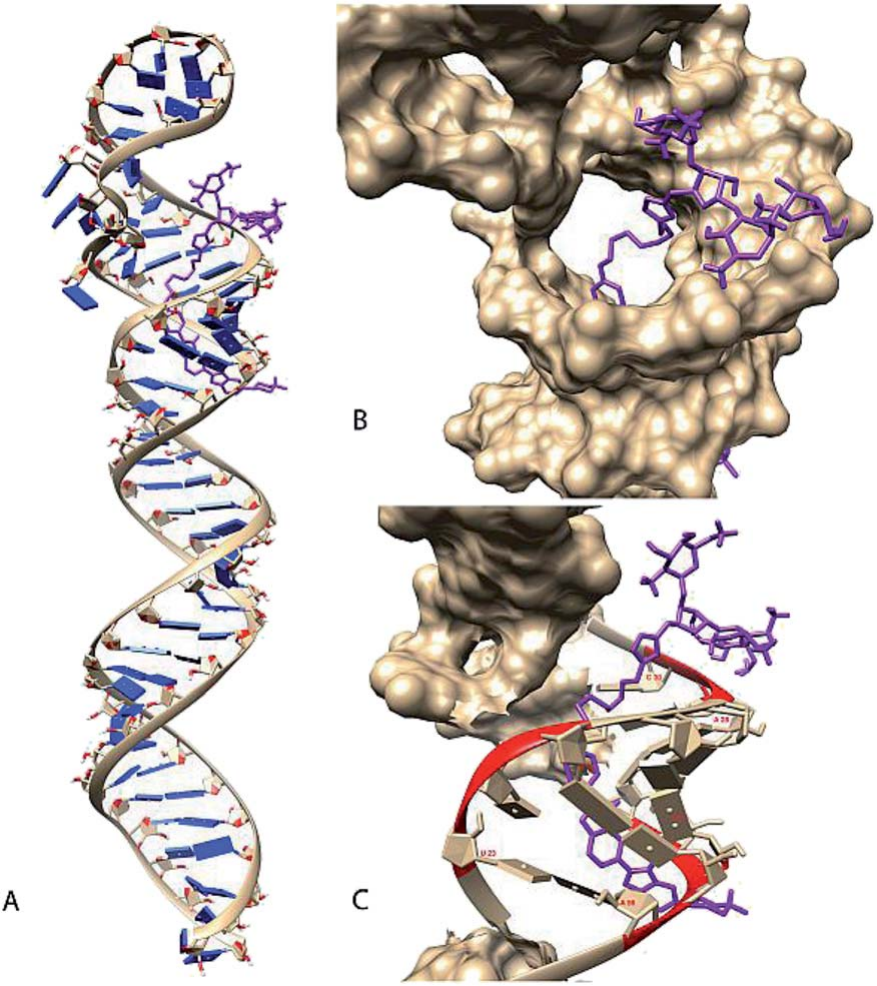
Manually selected model (Rank 3) of compound **5** docked with pre-miR-27a (A). Docked model of molecule (coloured in purple) bound to the pre-miR-27a having a binding energy of –7.72 kcal mol^–1^ (B). Magnified view of the docked structure showing the solvent accessible surface area (SASA) of the mirRNA (C) and contacts (coloured in red) between the molecule and pre-miR-27a. Compound **5** is shown to make contacts in regions between 23–25, 28–30 and 53–56 of mir-27a. The SASA and contacts were calculated using Chimera.

**Fig. 5:**
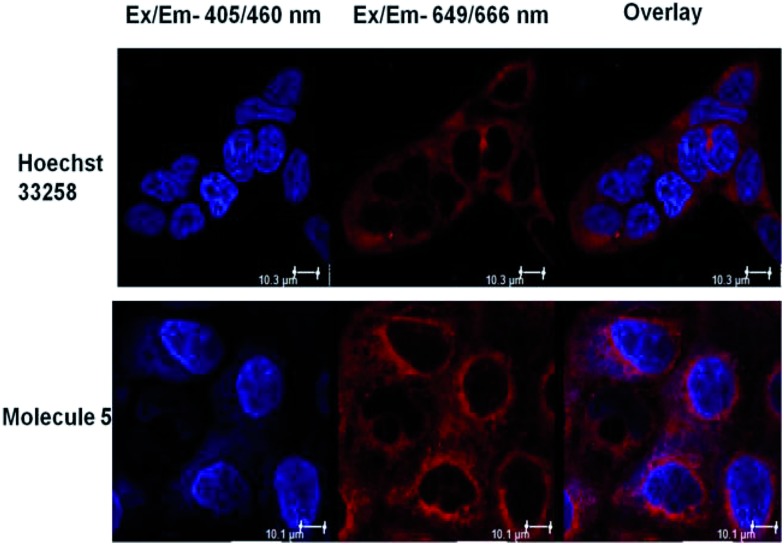
Representative image depicting localisation of Hoechst 33258 and neomycin–bisbenzimidazole conjugate **5**. The cell membrane and cytoplasm of MCF-7 cells is stained with CellMask deep red stain excited at 649 nm. Hoechst-33258-treated cells show clear nuclear localisation (blue) whereas conjugate-**5**-treated cells show nuclear as well as cytoplasmic localisation after 48 h of treatment, as seen in the overlay image. The white scale bar represents 10 μm.

We also ascertained the uptake of neomycin–bisbenzimidazole conjugates (data for conjugate **5** is shown in Fig. 4) in MCF-7 and examined its intracellular localization. For this, MCF-7 cells were treated with conjugate **5** at 5 μM and live cells were imaged after 48 h by confocal microscopy. As a negative control, we treated Hoechst 33258 at same concentration (5 μM). The cells were treated with CellMask stain (deep red) which marks the plasma membrane and cytoplasm. Hoechst 33258 showed a distinct localisation in the nucleus itself, as compared to conjugate **5** which was present both in the nucleus and distributed throughout cytoplasm, with few punctate vesicular structures also reported elsewhere (Fig. 5).^41^ Hoechst 33258 fits optimally in the binding pocket of the minor groove of DNA and thus occupies the nucleus densely. Conjugation of Hoechst 33258 with neomycin perturbs its structure such that it is less amenable to the binding pocket in the minor groove and has fewer tendencies to localize in nucleus itself. Distribution of the conjugate in cytoplasm is also explained by the fact that neomycin has the propensity to confine to the cytoplasm^41^ and hence can drive the conjugate to localise there.

In most cellular contexts, the final outcome of miRNA mediated target repression is reduced protein expression.^42^ PHB is a well-documented tumor suppressor and a target of miR-27a.^24^,^43^,^44^ High expression of miR-27a in MCF-7 cells leads to lower endogenous levels of PHB. Thus, we wanted to inspect further the ability of potential inhibitors to down-regulate miR-27a and thereby increase PHB levels. To do so, we treated the MCF-7 cells with neomycin–bisbenzimidazole conjugates at 5 μM for 48 h, isolated proteins and performed western blotting (Fig. S7, ESI†). Compound **3** and **5** were found to significantly up-regulate PHB levels as compared to the untreated control.

**Fig. 6:**
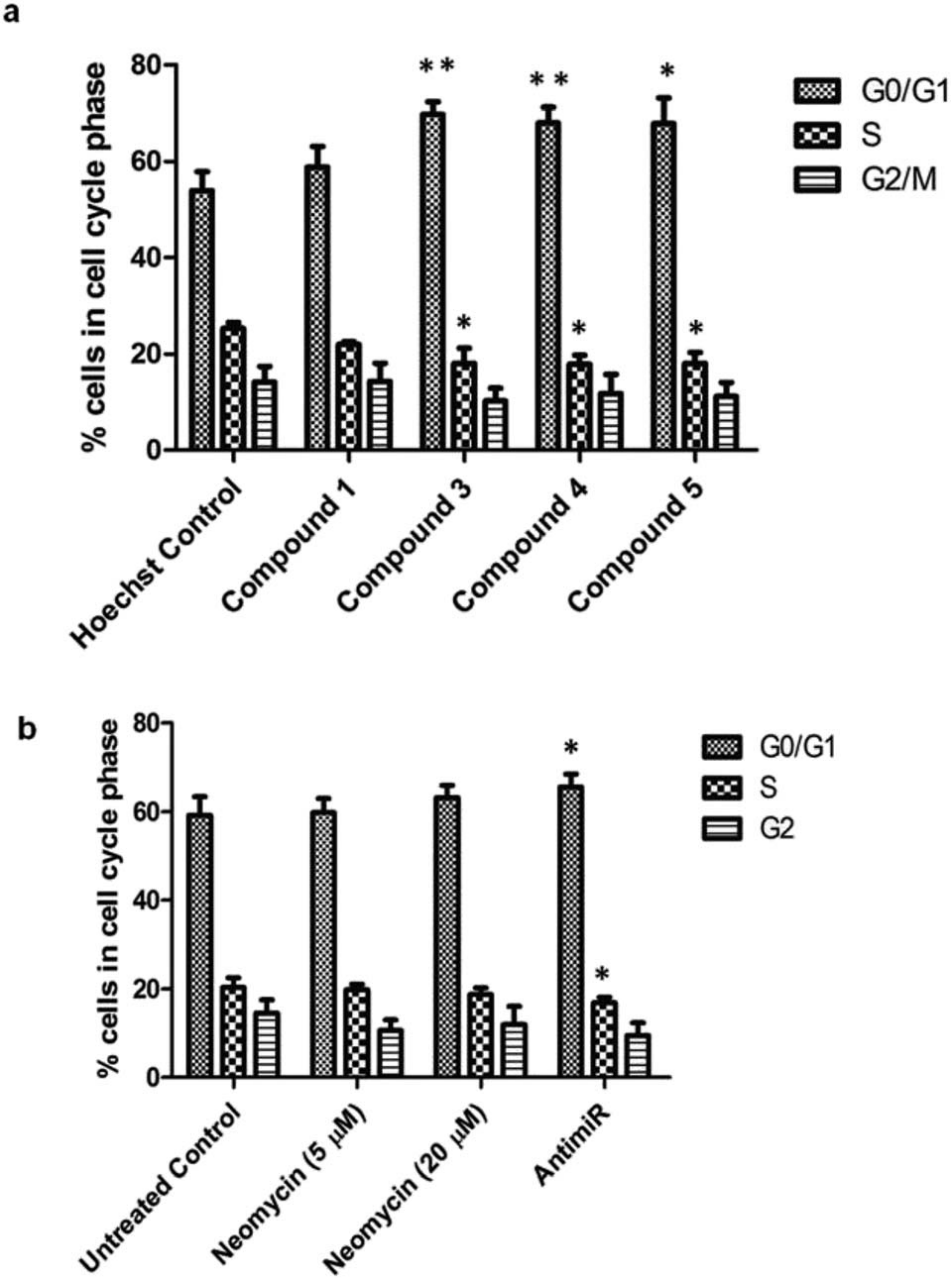
Percentage of cells in various cell cycle phases upon treatment with neomycin–bisbenzimidazole compounds. (a) Compounds **3**,** 4** and **5** induce a G0/G1 cell cycle arrest and decrease in the S-phase population as compared to the Hoechst control. Compound **1** had a minimal effect on cell cycle distribution at each phase. (b) As compared to the control, antimiR treatment caused an increase in the G0/G1 population. The parent compound, neomycin, only had a minor change. Results are expressed as means ± SD for three replicate determinations for each treatment group; *p < 0.05; **p < 0.01 (Student's t-test).

The oncogenic activity of miR-27a might be due to deregulation of cell cycle checkpoints. miR-27a is involved in cell cycle progression by regulating tumor suppressor target genes like FOXO1a and Fbw7.^25^,^45^ Overexpression of oncogenic miR-27a leads to improper distribution of cells in different phases of cell cycle and promotion of G1-to-S phase-transition, resulting in enhanced proliferation.^46^ We investigated the effect of miR-27a inhibition on the cell cycle after treatment with the most potent compounds (**3**, **4** and **5**). We treated the MCF-7 cells with each of the compounds or compound **1** for 48 h, stained with propidium iodide and monitored by flow cytometry (Fig. 6, Fig. S8 of ESI†). Compared to the Hoechst 33258 control, the compounds **3**, **4** and **5** showed a significant increase in the G0/G1 phase (∼15.8, ∼14% and ∼13.9%, respectively) and a minor but significant decrease in the S phase (∼7.3%, 7.46% and ∼7.26%) suggesting inhibition of proliferation. The G2/M phase of the cell cycle remained minimally affected by the treatments. However, compound **1** did not have any significant effect on the distribution of cells in any of the cell cycle phases. This pattern of G0/G1 arrest was comparable to the antimiR treated cells. The neomycin–bisbenzimidazole conjugates at 5 μM were more effective than parent compound neomycin at 5 μM and also at 20 μM. Hence, inhibition of miR-27a by these potent bivalent ligands might serve in decreasing the proliferation by arresting the cells at the G0/G1 stage and delaying the G1-to-S transition.

## Conclusions

New approaches aimed at enhancing both the affinity and specificity of small molecules are central to the development of nucleic-acid-based therapeutics. Here we show that chemical conjugation of two RNA binding molecules, leads to profound changes in the binding affinity towards target miR-27a and downregulation of the mature miRNA levels. The results obtained in this study lead us to draw the following conclusions: (a) neomycin–bisbenzimidazole conjugates demonstrate enhanced potency for downregulating miR-27a levels even at a ∼four fold lower dosage than parent molecules. This shows that conjugation leads to significant enhancement in efficacy. (b) The binding of neomycin–bisbenzimidazole conjugates is affected by the linker length and composition. (c) Out of all compounds tested, neomycin–bisbenzimidazole conjugates **3–5** were found to be the most effective and consistent in targeting and inhibiting miR-27a as compared to their parent compounds neomycin and Hoechst 33258, respectively. This also signifies the role of optimal linker length and composition in its precise fit into the spatial secondary conformation of pre-miR-27a. (d) Fluorescence-based determination of association constants for pre-miR-27a show close to nanomolar affinities of neomycin–bisbenzimidazole conjugates **3–5** (*K*_a_ = 1.2 to 7.4 × 10^8^ M^–1^) establishing these compounds as one of the tightest binders of miRNA to date. (e) Molecular modelling experiments suggest a mixed groove binding and stacking interaction of the two binding moieties at distinct sites without interfering with their respective binding domains. (f) Cell cycle analysis studies show inhibition of cell proliferation possibly by arresting cells at the G0/G1 stage and prolonging the G1 to S transition. (g) Neomycin–monobenzimidazole conjugates (**8–12**) did not lead to significant increase in the PHB levels suggesting the need for a bisbenzimidazole moiety for effectiveness in the binding.

miRNAs constitute a major and abundant class of ncRNAs that influence nearly all fundamental biological processes. Small molecules targeting oncogenic miRNAs which are overexpressed in cancer are an innovative and promising therapeutic strategy. This study underscores the finding that ligands that bind to non-competing sites on the same nucleic target can be covalently conjugated for a much better overall response to the target sequence. Clearly, the linker length and its composition are key determinants of the optimal binder. The findings of this work open new avenues towards more focused design of small molecules that target miRNAs. Conjugation of hits from high throughput screening endeavours or perhaps appropriately spaced small molecule–antisense oligonucleotide (ASO) conjugates could bring additional promising leads towards miRNA based cancer therapeutics. Undoubtedly, structural studies aimed at deciphering the microscopic details of small molecule interaction would provide a strong impetus to current drug design efforts in this area.

## Experimental

### Synthesis

Complete synthesis details and characterization for all newly synthesized compounds are provided in the ESI.†

### Cell culture

Human breast cancer cell line, MCF-7, obtained from American Type Culture Collection (Manassas, VA) was routinely maintained in Dulbecco's Modified Eagle Medium (DMEM medium, High Glucose, GIBCO) supplemented with 10% fetal bovine serum (FBS) without antibiotic and antimycotic at 37 °C in humidified air containing 5% CO_2_ air atmosphere.

### MTT assay

To determine if the compounds show direct cytotoxic effects due to conjugation with varying linkers of different length and composition, we performed a cell viability assay. Briefly, cells (6 × 10^3^ cells per ml) were seeded into 96-well plates and treated with all the synthesized compounds (twelve), parent compounds neomycin and Hoechst 33258 at 5 μM, and neomycin at 20 μM. The cells were incubated for 24 h at 37 °C. An MTT solution (3-(4,5-dimethylthiazol-2-yl)-2-5-diphenyltetrazolium bromide) with 0.5 mg ml^–1^ as working concentration was added to each well, and cells were incubated for 3 h at 37 °C. The supernatants were carefully removed, and formazan crystals were dissolved in 200 μl of dimethyl sulfoxide (DMSO). The absorbance was measured at 565 nm on a microplate reader and cell viability was calculated relative to untreated control cells.

### Luciferase screening

The *in cellulo* dual luciferase screening was performed using the psiCHECK-2-prohibitin vector, as described previously,^4^ where the endogenous target of miR-27a (Prohibitin), is fused downstream of *Renilla* luciferase gene, with firefly luciferase for normalization. MCF-7 cell line is reported to have high endogenous levels of miR-27a. Approximately 2 × 10^4^ cells were seeded equally in each well of a 24-well plate. Next day, 200 ng of the dual luciferase construct (psiCHECK-2-prohibitin vector) was transfected at ∼60% confluency using Lipofectamine 2000 transfection reagent (Invitrogen). The cells were incubated at 37 °C for 4 h followed by the replenishment of transfection media with DMEM growth media (500 μL). At the same time, cells were treated with 5 μM of neomycin–benzimidazole conjugates for 48 h. Post treatment, cells were lysed in 100 μl of 1X Passive Lysis Buffer (Promega) and centrifuged at 14 000 rpm for 15 min. The supernatants were assayed for *Renilla* and firefly luciferase signal using the dual-luciferase reporter assay kit (Promega), according to manufacturer's protocol. *Renilla* luciferase values were normalized using firefly luciferase values. The neomycin–bisbenzimidazole compounds treated were compared to vector control.

### In vitro transcription of pre-miR-27a

First, a DNA template was synthesized using primer extension method. Two partially overlapping oligonucleotides (forward and reverse) were used to make a hybrid duplex template from which *in vitro* transcription could be carried out. Forward oligonucleotide containing T7 promoter site (sequences in bold) **5′TAATACGACTCACTATAGGG**CTGAGGAGCAGGGCTTAGCTGCTTGTGAGCAGGGTCCACACCAAGTCGTGTTCACAGTGG 3′ and reverse oligonucleotide 5′CTGGGGGGCGGAACTTAGCCACTGTGAACACGACTTGGTGTGGACCCTGCTCACAAGCAGCTAAGCCCTGCTCCTCAGCC 3′ were mixed at 2 μM concentration each. To the reaction mixture, Taq polymerase (5 U), dNTPs (0.2 mM), Taq polymerase buffer (1X) and MgCl_2_ (2 mM) was added. The reaction mixture was denatured by heating at 95 °C for 5 minutes followed by snap-chilling on ice for 10 minutes, followed by primer extension incubation at 72 °C for 30 minutes. The hybrid template with T7 promoter was first gel checked for its proper size and used for *in vitro* transcription by using Megascript® High Yield Transcription Kit (Ambion Inc.) following manufacturer's instructions. The pre-miR-27a substrate was loaded, eluted and purified from 15% denaturing PAGE.

### Fluorescence titration

To determine the binding affiinity of neomycin–bisbenzimidazole compounds towards IVT purified pre-miR-27a, fluorescence titration experiment was carried out in Fluoromax 4 (Spex) spectrofluorometer equipped with a thermoelectrically-controlled cell holder (quartz cuvette 1 cm × 1 cm). Initially, pre-miR-27a was folded in buffer A (10 mM sodium cacodylate buffer, 1 mM MgCl_2_, and 10 mM NaCl) heated at 90 °C and cooled slowly at room temperature. The fluorescence spectra of the neomycin–bisbenzimidazole conjugates (compounds 3–7) were monitored after serial addition with increasing concentrations of pre-miR-27a. The excitation wavelength used was 350 nM. The excitation and emission slit widths were kept at 5 nm and 10 nM, respectively. The fluorescence titration experiments were carried out in buffer A at 25 °C and pH 7.5. pre-miR-27a was added serially followed by rapid mixing to a solution of fixed compound concentration (500 nM). The change in the fluorescence intensity at wavelength of fluorescence maxima (*λ*_max,fluor_) was monitored as a function of RNA concentration till no visible change in the fluorescence intensity was observed on further addition (after two minutes of incubation). The binding affinity between the pre-miR-27a and the neomycin–bisbenzimidazole conjugates was thus obtained using following expressions.

At any given ligand/RNA concentration ratio, the overall measured fluorescence intensity can be defined as the sum of fluorescence from free form and the bound ligand as described by the equation

1 *F* = (1 – *α*)*F*_0_ + *αF*_b_

where *F* is the observed fluorescence intensity at each titrant concentration; *F*_0_ is the fluorescence of free fluorophore and *F*_b_ are the fluorescence intensity of the bound fluorophore and *α* is the mole fraction of RNA in bound form. If we assume 1 : 1 stoichiometry of binding, the equilibrium association constant, *K*_a_, between the pre-miR-27a and neomycin–bisbenzimidazole compounds is related to the total compound concentration, [R]_0_ and the added RNA concentration, [L]_t_, through2
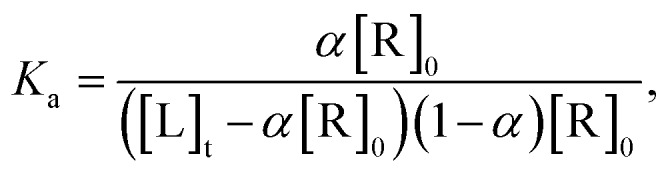

3 *α*^2^[R]_0_ – *α*([R]_0_ + [L]_t_ + 1/*K*_a_) + [L]_t_ = 0

Solving quadratic eqn (3)4
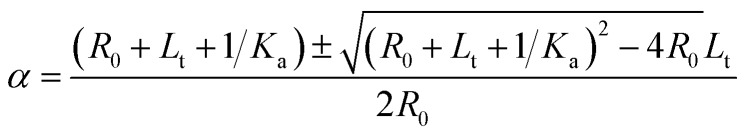



Using eqn (1) and (4), we obtain5
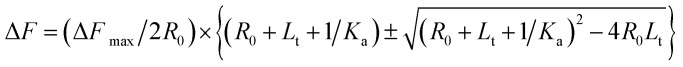
where,

6 Δ*F* = *F* – *F*_0_

and

7 Δ*F*_max_ = *F*_max_ – *F*_0_

The plot of change in fluorescence normalized with respect to the maximum fluorescence change (Δ*F*/Δ*F*_max_) *versus* pre-miR-27a concentration ([L]_t_) was fitted using eqn (5), from which *K*_a_ values were obtained.

### cDNA synthesis

MCF-7 cells cultured in DMEM growth medium were seeded at equal densities in 24-well plate (2 × 10^4^ cells per well), treated with neomycin–bisbenzimidazole conjugates at a cell confluency of ∼60% with a final concentration of 5 μM. Cells were incubated for 48 h after treatment, following which the growth medium was removed, washed with 1X PBS and RNA isolation was done using TRizol® Reagent (Invitrogen). cDNA was prepared by reverse transcription of 2 μg of total RNA by miRNA specific stem loop RT primers and random nonamers (for U6) as supplied by Reverse Transcriptase Core Kit (cat no. RT-RTCK-03 Eurogentec, USA). Stem-loop qPCR strategy was used to design the primers to specifically reverse transcribe miR-27a.^5^ Primers used for qRT-PCR is listed below:

Forward primer (miR-27a): 5′ACACTCCAGCTGGGTTCACAGTGGCTAAG 3′.

Stem-loop primer (miR-27a): 5′CTCAACTGAATTGCCGACTCCACGACACCAGTTGAGGCGGAACT 3′.

Common reverse primer: 5′ GTGTCGTGGAGTCGGCAATTC 3′.

Briefly, the extracted RNA were treated with DNase (Fermentus) for 30 min at 37 °C in presence of 10X DNase buffer supplemented with MgCl_2_. Inactivation of DNase was carried out at 65 °C for 15 min. The DNAse treated RNA was then mixed with 1 μl of miR-27a stem loop primer of 10 μM and 1 μl of 2.5 mM random nonamers. The reaction cocktail was heated at 65 °C for 5 min and cooled to room temperature. The other reaction components (1X reaction buffer, 10 mM MgCl2, 1 mM dNTPs, 0.8 U μl^–1^ RNAse inhibitor, 2.5 U μl^–1^ of Euroscript Reverse Transcriptase enzyme) were added to set up a 20 μl reaction. The reaction proceeded at 48 °C for 60 min, followed by inactivation of the RT enzyme at 95 °C for 5 min.

### Quantitative real time PCR

Once the cDNA was synthesized, real time qPCR was carried out to detect expression of miR-27a using Sybr-green I PCR master mix (Applied Biosystems) on Roche Lightcycler 480. All reactions were run in triplicate including a non-template control. The PCR reaction was carried out in 15 μl volume with 1X Sybr-green I PCR master mix, 2 μl of cDNA, 0.33 μM of miR-27a specific forward primer, 0.33 μM of common reverse primer. For the endogenous control, U6, cDNA synthesized by random nonamers was used as a template and the following primers were used. Forward primer (U6): 5′ CTCGCTTCGGCAGCACATATACT 3′. Reverse primer (U6): 5′ ACGCTTCACGAATTTGCGTGTC 3′. The data was normalized with respect to the reference gene U6. Relative expression was calculated using the comparative Ct method.^6^

### Modeling of hsa-miR-27a

The miRNA was modeled using MC-Fold|MC-Sym pipeline.^7^ The secondary structure and the resulting three-dimensional structure are given in Fig. S4a (ESI†). The structure was then minimized with all restraints removed, and a steepest descent minimization of 1000 step, followed by a conjugate gradient minimization of 1500 steps. The long-range cut-off for non-bonded interactions during the minimization was 8 Å.

### Small molecule preparation and docking

The compounds were drawn using ChemDraw 8 software. The two-dimensional molecules were then converted to three-dimensional structures using OpenBabel.^47^ The energy minimization was performed using Maestro9.8.^48^ Docking was performed using AutoDock 4.2.6 and MGLTools of the Scripps Research Institute.^40^,^49^ Hydrogen atoms and Kollman and Gasteiger partial charges were assigned to the ligands (compounds) with all torsions allowed during the docking. A grid box was built around the entire mirRNA to allow the ligands to move freely and affinity maps of the protein (500 × 500 × 500 with random number generator seeded) were calculated using AutoGrid. Fifty Lamarckian Genetic Algorithm (LGA) runs with 250 000 000 number of energy evaluations were performed. The docking results were ranked according to the lowest docked energy for the ligands in which the neomycin group interacts with the mirRNA's stem-loop region in the major groove.^50^,^51^ Molecular graphics and analyses were performed with the UCSF Chimera package.^52^

### All atomistic molecular dynamics simulation

To check for conformational stability, molecular dynamic simulation was done using GROMACS 4.6.1.^53^ All atomistic simulations were carried out using the CHARMM36 all-atom force field (November release)^54^,^55^ using periodic boundary conditions. The starting docked models were solvated in a periodic box with TIP3 water model. Na ions were added to the solvent to neutralize the electrical net charge of the protein. Each system was then minimized for 50 000 steps using a steepest-decent algorithm. The NPT ensemble was used for production simulation. Systems were simulated at 310 K, maintained separately for miRNA, docked molecule, water by a Berendsen thermostat with a time constant of 1 ps. Pressure coupling was done employing a Berendsen barostat using a 1 bar reference pressure and a time constant of 2 ps. Electrostatic interactions were calculated using the Particle Mesh Ewald (PME) summation. All the molecular dynamic simulations were carried out on the CSIR-4PI 360 TF Supercomputer.

### Western blot

A 24-well plate was seeded at ×10^4^ cells per well, 24 h prior to treatment such that it attains a ∼60% cofluency. The LNA modified antimiR-27a was transfected at 100 nM with Lipofectamine 2000 reagent (Invitrogen) according to manufacturer's instructions. The cells were incubated for 4 h at 37 °C in a CO_2_ incubator. Post transfection, the OPTIMEM medium was replaced by DMEM growth media. At the same time, cells were treated with neomycin–bisbenzimidazole conjugates and incubated for 48 h. The cells were washed with 1X PBS, post 48 h and lysed with RIPA Lysis and Extraction Buffer (Thermo Scientific). The pellet was harvested and total cell lysate was transferred to a fresh tube. A BCA protein Assay Reagent Kit (Pierce) was used to measure protein concentration in the cellular lysate. Samples containing equal amounts of protein (40 μg) were loaded and separated on 12% SDS-PAGE and transferred to a nitrocellulose membrane. The blots were then probed with blocking reagent (5% BSA) for 2 h and incubated with primary antibodies overnight at 4 °C, specific for prohibitin (PHB) protein (1 : 500, Abcam) and β-tubulin (1 : 2000, CST). Subsequently, the blots were washed thrice with 1X TBS supplemented with 0.1% Tween-20 for 15 min each. The blots were next incubated in secondary antibody conjugated with alkaline phosphatase (1 : 10 000) and developed using BCIP-NBT solution (SIGMA).

### Live cell imaging

MCF-7 cells (3 × 10^3^ cells per well) were grown overnight on an 8 well glass chamber, 0.7 cm^2^ per well (Thermo Scientific™ Nunc™ Lab-Tek™). Next day, cells were independently treated with Hoechst 33258 alone and one representative compound 5 at 5 μM. The cells were incubated for 48 h in humidified conditions having 5% CO_2_. The cells were treated with CellMask deep red stain (Invitrogen) for 30 minutes at 1 : 1000 dilutions. At the endpoint, the cells were washed with 1X PBS, submerged in 200 μl DMEM (without phenol red, Invitrogen) and subjected to live cell imaging in LEICA laser scanning multiphoton confocal microscope. The cells were imaged at 60× magnification in bright field, blue channel (Ex/Em-405/460 nm), far red channel (Ex/Em-649/666 nm) and images were overlaid.

### FACS-mediated cell cycle analysis

For cell cycle analysis, MCF-cells were seeded in 24 well plates (2 × 10^4^ cells per well) and treated with either neomycin–bisbenzimidazole compounds (**1**, **3–5**) at 5 μM, or with parent compounds neomycin at 5 and 20 μM, Hoechst at 5 μM and antimiR at 100 nM. The cells were incubated for 48 h at 37 °C. Following treatment, cells were trypsinized, centrifuged and washed twice with 1X PBS and fixed in 70% ethanol overnight at –20 °C. The pellet was resuspended in 200 μl of 4 mM sodium citrate buffer containing 0.1% Triton X-100. Then 20 μl of 1 mg ml^–1^ RNAse solution was added and incubated for 2 h at 37 °C. Following incubation 30 μl of 50 μg ml^–1^ propidium iodide (BD Biosciences) was added and the cells kept at room temperature in the dark for 30 min. Cells were analysed on BD Accuri C6 flow cytometer and propidium iodide (PI) fluorescence was collected through a FL2 filter (585/40 nm bandpass filter). A minimum of 10 000 events were recorded on a dot plot of FL2-A *vs.* FL2-H. Following singlet discrimination and exclusion of cell aggregates, data was analysed on BD Accuri software. The cells treated with antimiR and neomycin stained with PI was compared with untreated cells stained with PI. The cells treated with neomycin–bisbenzimidazole compounds (having Hoechst) and stained with PI were compared with Hoechst treated cells stained with PI.

## Supplementary Material

Supplementary information
