# Supplementary material for: Potent inhibition of miR-27a by neomycin–bisbenzimidazole conjugates
Source: Chem Sci. 2015 Jul 9;6(10):5837–46. doi: 10.1039/c5sc01969a (PMC5947510; doi:10.1039/c5sc01969a)
Supplement: Supplementary file 1 [file SC-006-C5SC01969A-s001.pdf]

Electronic Supplementary Material (ESI) for Chemical Science.
This journal is © The Royal Society of Chemistry 2015

Potent inhibition of miR-27a by Neomycin-bisbenzimidazole conjugates

Smita Nahar, [a,b] **Nihar Ranjan,**[c] **Arjun Ray,** [a,b] **Dev P. Arya** [c] **, and Souvik Maiti***[a,b,d]

[a]*Academy of Scientific and Innovative Research (AcSIR), Anusandhan Bhawan,2 Rafi Marg, New Delhi-110001, India*

[b]*CSIR- Institute of Genomics and Integrative Biology, Mathura Road, Delhi-110020,India. E-mail: souvik@igib.res.in; Fax: +91-11-2766-7471; Tel: +91-11-2766-6156*

[c]*Department of Chemistry, Clemson University, Clemson, SC, United States 29634*

[d]*CSIR-National Chemical Laboratory, Dr. Homi Bhabha Road, Pune, 411008, India*
**correspondence to souvik@igib.res.in*

Table of Contents

Experimental Procedures ………3-25

Fig S1: Structures of neomycin-bisbenzimidazole conjugates **1-7**. ………..26

Fig S2: Structures of neomycin-monobenzimidazole conjugates **8-12**, neomycin and Hoechst 33258. ………..27

Fig S3: Relative Cell Viability of conjugates **1-12** ………..28

Fig S4a: Modelling of hsa-pre-mir-27a using mc-fold | mc-sym pipeline ……….. 29

Fig S4b: A view of bisbenzimidazole stacking with base pairs ………..30

Fig S5: RMSD plots of pre-mir-27a from the MD simulation for the best-docked poses of molecules with pre- mir-27a. ……….30

Fig S6: Distance plot between the centre of masses of the docked molecules and pre-mir-27a. ……….31

Fig S7: Western Blot to detect Prohibitin levels ……….32

Fig S8: Representative histogram plots of FACS mediated cell cycle analysis ……….33-34

^{1}H NMR, ^{13}C NMR, IR, and MALDI-TOF spectra of all new compounds for the synthesis of conjugates **8-12**. ……….34-86

References ……….87

Experimental procedures

***Synthesis of Neomycin-bisbenzimidazole conjugates* (1-7)**

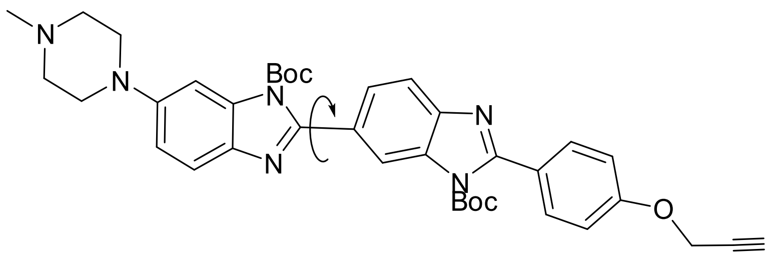

Synthesis of ***1b****.*

To a solution of **1a** (40.0 mg, 86.3 μmol) in dry THF (20.0 mL), NaH was added (50.0 mg, 2.00 mmol) at 0 °C. After 15 min stirring, $(Boc)_2O$ (100 mg, excess) was added to the mixture and it was allowed to slowly warm to room temperature . After 5h, the reaction mixture was concentrated under the stream of nitrogen. The crude product was purified by column chromatography on silica gel (5 micron mesh size) using dichloromethane-methanol (0-20 % methanol in dichloromethane) as eluent to obtain the desired product which eluted as two rotamers which moved distinctly during the TLC analysis. The rotamer I (R_f = 0.68 in dichloromethane:methanol 8.5:1.5 *v/v*) was obtained in 32 % yield and rotamer II (R_f = 0.59 in dichloromethane:methanol 8.5:1.5 *v/v*) was obtained in 19 % yield: ^1H NMR (500 MHz, methanol-d_4)(Rotamer I) δ 8.38 (d, J = 1.65 Hz ,1H), 7.84 (d, J = 8.38 Hz, 1H), 7.70-7.64 (5H), 7.21-7.18 (m, 3H), 4.88 (d, J = 2.24 Hz, 2H), 3.33 (peak masked by residual MeOH), 3.04 (t, J = 1.96 Hz, 1H), 2.76 (t, J = 4.78 Hz 4H), 2.45 (s, 3H), 1.47-1.40 (18H); MS (MALDI–TOF) *m/z* calcd for $C_{38}H_{42}N_6O$, 662.32, found 662.48 [M^+]; (Rotamer II) δ 8.40 (d, J = 1.32 Hz, 1H), 7.99 (d, J = 9.04 Hz ,1H), 7.84 (d, J = 8.60 Hz, 1H) , 7.72 (dd, J_1 = 8.2 Hz, J_2 = 2.13 Hz , 1H), 7.70-7.67 (m, 2H), 7.29- 7.18 (m, 4H), 4.88 (d, J = 2.39 Hz, 2H), 3.33 (peak masked by residual MeOH), 3.04 (t, J

= 2.39 Hz, 1H), 2.78 (t, *J* = 4.51 Hz, 4H), 2.46 (s, 3H), 1.47-1.42 (18H); MS (MALDI–TOF) *m/z* calcd. for $C_{38}H_{42}N_6O$, 662.32 found 662.41 [M$^+$].

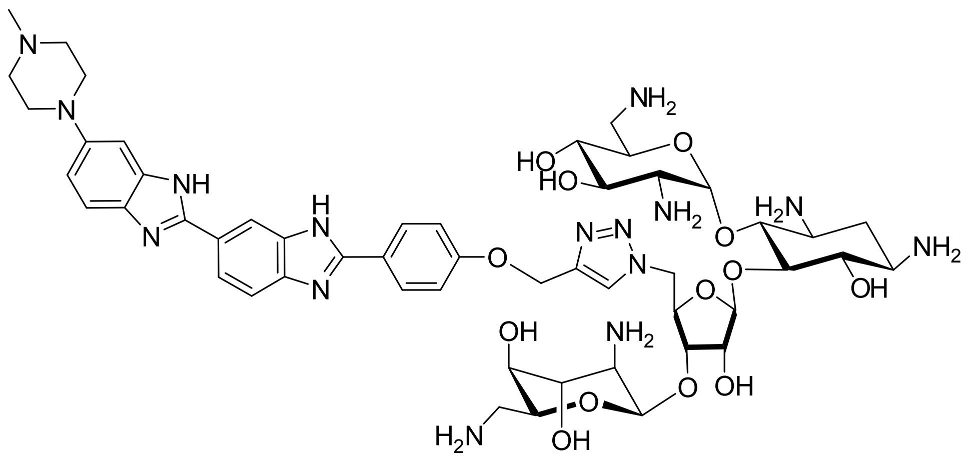

Synthesis of **1.**

To a solution of **1b** (rotamer I, R_f = 0.65 in dichloromethane-methanol *v/v*, 14.3 mg, 21.6 μmol) in ethanol (1.00 mL), a solution of Boc protected neomycin-azide (26.8 mg, 21.6 μmol) in ethanol (1.00 mL) was added followed by addition of a freshly made copper(I) catalyst made by adding aqueous solutions of $CuSO_4$ (1.40 mg, 9.00 μmol) and sodium ascorbate (3.40 mg, 18.0 μmol). The reaction mixture was stirred for 10h at room temperature under the darkness. TLC on silica gel indicated formation of the product. Volatiles were evaporated under reduced pressure. The crude product was purified using column chromatography on silica gel using dichloromethane-methanol (0-10 % methanol in dichloromethane) as eluent. This yielded Boc protected **1** (26.3 mg, R_f = 0.59 in dichloromethane: methanol 8.5:1.5, *v/v*). ^{1}H NMR (500 MHz, methanol-d_4) δ 8.36 (br, 1H), 7.83 (d, *J* = 8.98 Hz, 1H), 7.71-7.56 (m, 5H), 7.29 (d, *J* = 8.88 Hz, 2H), 7.21 (d, *J* = 8.98 Hz, 1H), 7.12 (s, 1H), 6.68-6.58 (br, 2H), 5.45-5.36 (m, 2H), 5.17 (s, br, 1H), 4.98 (2H), 4.74-4.29 (m, 3H), 4.20-3.88 (m, 2H), 3.79-3.37 (m, 15H), some proton signals are masked with residual MeOH from NMR solvent, 2.89 (m, 5H), 2.54 (s, 3H), 2.45-2.41 (m, 1H), 2.07-2.01 (m, 1H), 1.49-1.28 (m, 72H); MS(MALDI-TOF) *m/z* calcd. for $C_{91}H_{135}N_{15}NaO_{29}$ 1924.94, found 1925.33 [M+H]$^+$.

Boc protected **1** (8.7 mg, 4.57 μmol) was dissolved in dichloromethane (2.0 mL) followed by addition of trifluoroacetic acid (0.1 mL). The mixture was stirred for 4h at room temperature. Water (2.0 mL) was added to it. The mixture was washed with dichloromethane (3 × 1 mL). The aqueous layer was

lyophilized to afford the desired compound as pale yellow solid (5.9 mg, 38 % cumulative yield for two steps): UV λ_{max} = 338 nm in water; ^{1}H NMR (500 MHz, D_2O) δ 8.24 (s, 1H), 8.19 (s, 1H), 8.02-7.96 (m, 3H), 7.88 (d, J = 8.55 Hz, 1H), 7.65 (d, J = 8.54 Hz. 1H), 7.29-7.11 (m, 4H), 6.02 (d, J = 3.83 Hz, 1H), 5.35-5.34 (d, J = 3.34 Hz, 1H), 5.29 (s, br, 2H), 5.21 (s, br, 1H), 4.83-4.79 (dd, J_1 = 3.19Hz, J_2 = 2.71 Hz, 1H), 4.50 (m, 1H), 4.38 (t, J = 4.63 Hz, 1H), 4.21 (t, J = 4.63 Hz, 1H), 4.12 (t, J = 5.79 Hz, 1H), 4.02 (t, J = 8.68 Hz, 1H), 3.94 (t, J = 9.24 Hz, 1H), 3.90-3.77 (m, 5H), 3.74 (s, br, 1H), 3.62-3.42 (m ,7H), 3.39-3.08 (m, 10H), 2.88 (s, 3H), 2.38 (m, 1H), 1.82 (m, 1H) (some proton signals are overlapped with the NMR solvent peak) ; MS (MALDI-TOF) *m/z* calcd. for $C_{51}H_{71}N_{15}O_{13}$ 1101.53, found 1102.27 $[M+H]^+$; HPLC (t_R = 5.07 min, purity = 97.2%, elution method-100% H_2O (containing 0.1% TFA) to 100% acetonitrile in 15 minutes on a Supelcosil LC18 column at a flow rate of 2.0 mL/minute).

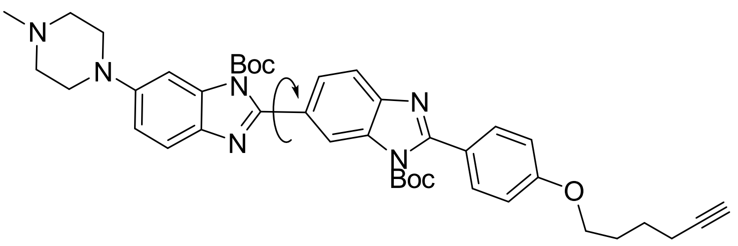

*Synthesis of **2b**.*

To a solution of **2a** (60.0 mg, 0.12 mmol) in dry THF (20 mL) NaH was added (97.0 mg, 3.88 mmol) at 0 °C. After 30 min stirring, $(Boc)_2O$ (0.1 g, 0.45 mmol) was added to the solution and the mixture was allowed to slowly warm to room temperature . After overnight stirring, the reaction mixture was concentrated under the stream of nitrogen and mixed with some silica powder (5 micron mesh size). The reaction mixture was purified using column chromatography on silica gel (5 micron mesh size) using dichloromethane-methanol (0-10 % methanol in dichloromethane) as eluent to obtain the desired product which eluted as two different rotamers (rotamer I 35.6 mg, 43 %; rotamer II 27.1 mg, 32 %) and a mixture of rotamer I and rotamer II (10 mg, 12 %): R_f = (0.53 for rotamer I and 0.46 for rotamer II respectively in dichloromethane:methanol 9:1 *v/v*); ^{1}H NMR (500 MHz, methanol-d_4) (rotamer I) δ 8.37-8.20 (two sets of doublets, 8.37 (d, J = 1.41 Hz) and 8.21 (d, J = 8.59 Hz), 1H), 7.97-7.82 (two sets of doublets, 7.97 (d, J = 1.46 Hz) and 7.83 (d, J = 8.34 Hz), 1H), 7.72-7.61 (m, 5H), 7.20 (dd, J_1

= 2.10 Hz , J_2 = 8.59 Hz, 1H), 7.11 (dd, J_1 = 3.07 Hz, J_2 = 8.59 Hz, 2H), 4.13 (t, J = 6.3 Hz, 2H), 3.34 (some peaks masked by the NMR solvent peak), 2.73 (t, J = 4.71 Hz, 4H), 2.98 (s, 3H) , 2.32-2.27 (m, 3H), 2.00- 1.94 (m, 2H), 1.78-1.72 (m, 2H), 1.48-1.40 (18H); ^{13}C NMR (75 MHz, methanol-d_4) δ 160.8, 155.7, 152.5, 150.0, 148.5, 142.5, 135.7, 134.7, 133.2, 130.7, 128.7, 125.8, 123.4, 119.8, 119.0, 118.3, 115.6, 113.5, 101.4, 86.0. 85.4, 83.2, 68.4, 67.3, 54.6, 49.4, 44.6, 29.6, 27.9, 26.3, 24.8, 17.4 ; MS (MALDI-TOF) *m/z* calcd for $C_{41}H_{48}N_6O_5$ 704.36 found 704.31 $[M]^+$. (rotamer II) δ 8.39-8.21 (two sets of doublets, 8.39 (d, J = 1.40 Hz) and 8.22 (d, J = 8.54 Hz), 1H) 7.99 (d, J = 8.89 Hz, 1H), 7.84 (d, J = 8.20 Hz, 1H) , 7.72 (dd, J_1 = 1.47 Hz, Hz, J_2 = 8.29 Hz, 1H), 7.68-7.65 (m, 2H), 7.14-7.11 (m, 2H), 7.29- 7.22 (m, 2H), 4.14 (d, J = 6.30 Hz, 2H), 3.33 (peak masked by residual MeOH), 2.73 (t, J = 4.58 Hz, 4H), 2.42 (s, 3H), 2.33-2.27 (m, 3H), 2.01-1.95 (m , 2H), 1.79-1.72 (m, 2H), 1.49-1.43 (18H); MS (MALDI-TOF) *m/z* calcd for $C_{41}H_{48}N_6O_5$ 704.36 found 704.39 $[M]^+$.

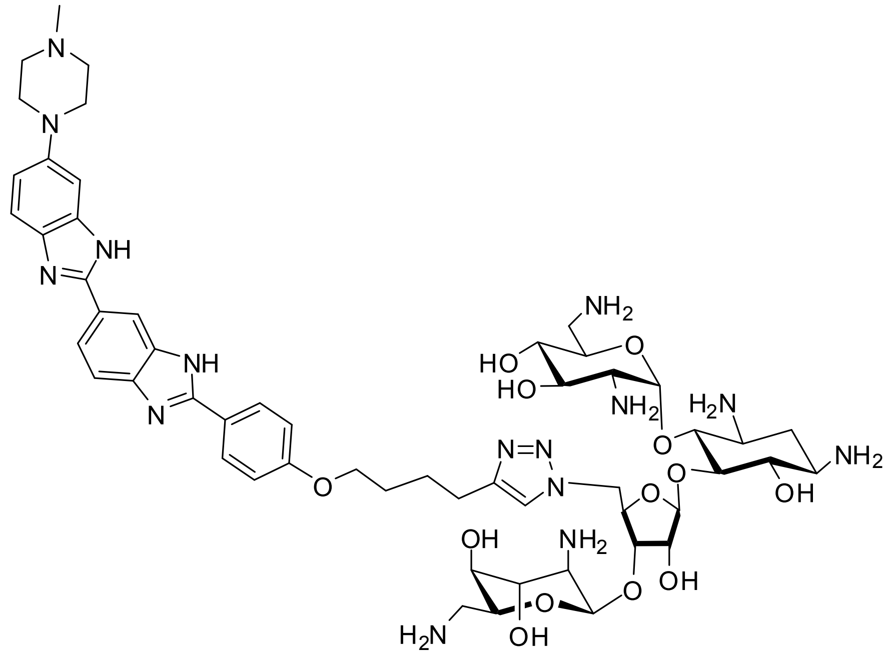

Synthesis of ***2****.*

To a solution of **2b** (rotamer I, R_f = 0.53 in dichloromethane:methanol 9:1 *v/v*, 26.6 mg, 37.7 μmol) in ethanol (3.00 mL), a solution of Boc protected neomycin-azide (47.0 mg, 37.7 μmol) in ethanol (1.00 mL) was added followed by by addition of a freshly made copper(I) catalyst made by adding aqueous solutions of $CuSO_4$ (1.6 mg, 10.0 μmol) and sodium ascorbate (4.0 mg, 20.0 μmol). The reaction

mixture was stirred for 12h at room temperature. TLC on silica gel indicated formation of the product. Solvents were evaporated under reduced pressure. The crude mixture was purified by column chromatography on silica gel (5 micron, mesh size) using dichloromethane-methanol (0-12 % methanol in dichloromethane) as eluent. This yielded diBoc protected DPA 166 (35.4 mg, $R_f = 0.40$ in dichloromethane: methanol 9:1 *v/v*): ^1H NMR (500 MHz, methanol-d_4) δ 8.36 (br, 1H), 8.02-7.81 (m, 2H), 7.70-7.57 (m, 4H), 7.20-7.11 (m, 4H), 6.72-6.58 (br, 2H), 5.44 (s, br, 1H), 5.19 (s, br, 1H), 5.17 (s, br, 1H), 4.63-4.16 (m, 6H), 4.10-3.47 (m, 14H), 3.34 (some proton signals are masked with residual MeOH from NMR solvent), 3.29-3.19 (m, 3H), 2.87 (t, *J* = 6.19 Hz, 2H), 2.76 (s, br, 4H), 2.45 (s, 3H), 1.96-1.90 (m, br, 4H), 1.49-1.30 (m, 72H); MS(MALDI-TOF) *m/z* calcd. for $C_{94}H_{141}N_{15}NaO_{29}$ 1968.94, found 1973.85 $[M+5H]^+$.

The boc protected conjugate **2** was then dissolved in dichloromethane (3.0 mL) followed by addition of trifluoroacetic acid (0.4 mL). The mixture was stirred for 6h at room temperature. Water (2.0 mL) was added to it. It was washed with dichloromethane (3 × 1 mL). The aqueous layer was lyophilized to afford the desired compound as pale yellow solid (32.3 mg, 40 % for two steps): UV λ_{max} = 338 nm in water; ^1H NMR (500 MHz, D_2O) δ 8.23 (s, br, 1H), 8.01-7.89 (m, 4H), 7.83 (s, br, 1H), 7.65 (d, *J* = 9.21 Hz, 1H), 7.28 (d, *J* = 9.21 Hz, 1H), 7.25 (s, br, 1H), 7.12 (d, *J* = 8.76 Hz, 2H), 5.94 (d, *J* = 3.22 Hz, 1H), 5.34 (d, *J* = 3.71 Hz, 1H), 5.21 (s, br, 1H), 4.64-4.60 (peaks overlapping with HDO signal), 4.51-4.46 (m, 1H), 4.35 (t, *J* = 4.41 Hz, 1H), 4.22 (t, *J* = 4.21 Hz, 1H), 4.13 (t, *J* = 2.37 Hz, 1H), 4.09- 4.03 (m, 2H), 4.01- 3.91 (m, 2H), 3.88-3.78 (m, 4H), 3.74 (s, br, 1H), 3.68 (t, *J* = 3.27 Hz, 1H), 3.60-3.56 (3H), 3.52-3.05 (m ,14H), 2.89 (s, 3H), 2.42-2.36 (m, 1H), 1.83-1.72(m, 5H); MS (MALDI-TOF) *m/z* calcd for $C_{54}H_{77}N_{15}O_{13}$ 1143.58 , found 1144.32 $[M]^+$; HPLC (t_R = 11.7 min, purity = 97.2%, elution method- 100% H_2O (containing 0.1% TFA) to 100% acetonitrile in 25 minutes on a Supelcosil LC18 column at a flow rate of 1.2 mL/minute).

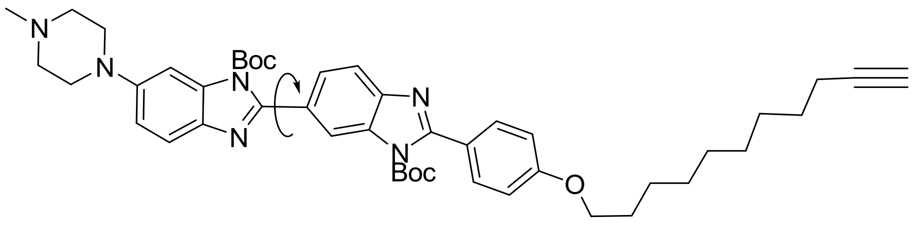

Synthesis of **3b**.

To a solution of **3a** (0.10 g, 0.17 mmol) in dry THF (40.0 mL) NaH was added (0.10 g, 4.16 mmol) at 0 °C. After 30 min stirring, $(Boc)_2O$ (0.20 g, 0.91 mmol) was added to the solution and the mixture was allowed to slowly warm to room temperature . After overnight stirring, the reaction mixture was concentrated under the stream of nitrogen and mixed with small amount of silica powder (5 micron mesh size). The crude product was purified by column chromatography on silica gel (5 micron mesh size) using dichloromethane-ethanol (0-15 % methanol in dichloromethane) as eluent to obtain the desired product which eluted as two different rotamers (rotamer I 33.7 mg, 25% and rotamer II 42.0 mg, 31 %) and a mixture of rotamer I and rotamer II (41.4 mg, 31%) : R_f = (0.38 for rotamer I and 0.23 for rotamer II respectively in dichloromethane:ethanol 9:1 *v/v*); ^{1}H NMR (300 MHz, methanol-d_4) (rotamer I) δ 8.36-8.19 (two sets of doublets, 8.36 (d, *J* = 1.1 Hz) and 8.21 (d, *J* = 8.48 Hz), 1H), 7.96-7.81 (two sets of doublets, 7.86 (d, *J* = 1.1 Hz) and 7.81 (d, *J* = 8.28 Hz), 1H), 7.72-7.60 (m, 5H), 7.20 (dd, J_1 = 2.30 Hz , J_2 = 8.90 Hz, 1H), 7.11 (dd, J_1 = 2.01 Hz, J_2 = 8.79 Hz, 2H), 4.10 (t, *J* = 6.26 Hz, 2H), 3.34 (some peaks masked by residual MeOH peak from the NMR solvent), 2.72 (t, *J* = 4.71 Hz, 4H), 2.42 (s, 3H) , 2.21-2.16 (m, 2H), 1.89- 1.79 (m, 2H), 1.58-1.37 (m, 30H); ^{13}C NMR (75 MHz, methanol-d_4) δ 160.9, 155.7, 152.6, 150.0, 148.5, 142.5, 141.2, 138.7 135.7, 134.7, 130.7, 128.7, 125.8, 123.6, 119.0, 118.2, 115.7, 113.8, 101.4, 86.0. 85.4, 83.7, 67.9, 67.8, 54.6, 49.5, 44.6, 33.4, 29.2, 29.1, 29.0, 28.8, 28.7 (two peaks), 28.3 (two peaks), 26.4, 25.7; MS (MALDI-TOF) *m/z* calcd for $C_{46}H_{58}N_6O_5$ 774.44 found 774.03 $[M]^+$. (rotamer II) ^{1}H NMR (300 MHz, methanol-d_4) δ 8.38-8.19 (two sets of doublets, 8.38 (d, *J* = 1.1 Hz) and 8.21 (d, *J* = 8.47 Hz), 1H) 7.99-7.82 (m, 2H), 7.73-7.63 (m, 3H), 7.28-7.20 (m, 2H), 7.09 (dd, J_1 = 1.92 Hz , J_2 = 8.68, 2H), 4.09 (t, *J* = 6.20 Hz, 2H), 3.33 (peak masked by residual MeOH), 2.72 (t, *J* = 4.74 Hz, 4H), 2.41 (s, 3H), 2.20-2.15 (m, 2H), 1.88-1.79 (m , 2H), 1.55-1.30 (30H); ^{13}C NMR (75 MHz, methanol-d_4) δ 161.0, 155.5, 154.0. 149.4, 142.7, 141.2, 138.7, 134.6, 133.2, 130.7, 128.4, 125.9, 123.3, 120.0, 118.2, 116.3, 114.8, 113.3, 105.4, 86.0, 85.5, 83.6, 67.9, 67.8, 54.6, 49.5, 44.6, 33.4, 29.2, 29.1, 29.0, 28.8, 28.7, 28.3, 26.4, 26.3, 25.7,17.6; MS (MALDI-TOF) *m/z* calcd for $C_{46}H_{58}N_6O_5$ 774.44 found 774.03.

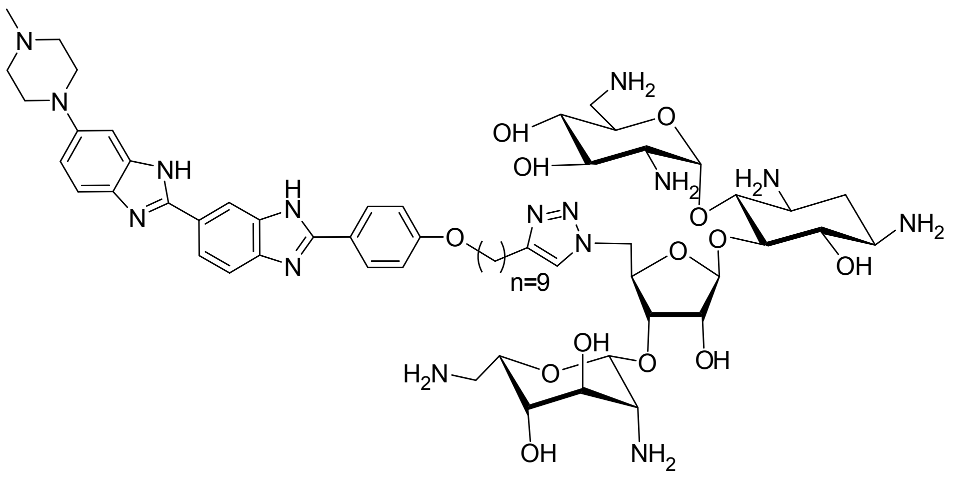

Synthesis of **3**.

To a solution of **3b** (rotamer I 28.7 mg, 37.0 μmol) in ethanol (3.50 mL), a solution of Boc protected neomycin-azide (46.0 mg, 37.0 μmol) in ethanol (1.00 mL) was added followed by addition of a freshly made copper(I) catalyst made by adding aqueous solutions of $CuSO_4$ (1.60 mg, 10.0 μmol) and sodium ascorbate (3.96 mg, 20.0 μmol). The reaction mixture was stirred for 42h at room temperature. TLC on silica gel indicated formation of the product. Solvents were evaporated under reduced pressure. The crude mixture was purified by column chromatography on silica gel (5 micron, mesh size) using dichloromethane-ethanol (0-12 % ethanol in dichloromethane) as eluent. This yielded Boc protected **3** (35.2 mg, R_f = 0.45 in dichloromethane: ethanol 9:1 *v/v*). The starting material alkyne was also recovered (11.2 mg). The Boc protected conjugate **3** was then dissolved in dichloromethane (3.0 mL) followed by addition of trifluoroacetic acid (0.6 mL). The mixture was stirred for 2h at room temperature. Water (4.0 mL) was added and it was washed with dichloromethane (3 × 1 mL). The aqueous layer was lyophilized to afford the desired compound as pale yellow solid (24 mg, 30 % cumulative yield for two steps): UV λ_{max} = 339 nm in water; ^{1}H NMR (500 MHz, D_2O) δ 8.14 (br, 1H), 7.98-7.77 (m, 6H), 7.61 (d, *J* = 8.83 Hz, 1H), 7.24-7.06 (br, 3H), 5.94 (d, *J* = 3.08 Hz, 1H), 5.34-5.33 (br, 2H), 5.20 (s, br, 1H), 4.66 (peaks overlapping with HDO signal), 4.50-4.46 (m, 2H), 4.36 (t, *J* = 5.23 Hz, 2H), 4.21 (t, *J* = 5.23 Hz, 2H), 4.12 (t, *J* = 3.35 Hz, 1H), 4.04- 3.94 (m, 4H), 3.87-3.66 (m, 6H), , 3.61-3.55 (3H), 3.50 (br , 2H), 3.45- 3.08 (m, 16H), 2.88 (s, 3H), 2.86 (br, 1H), 2.62-2.58 (m, 2H), 2.40-2.34 (m, 1H), 1.87-1.79 (m, 1H), 1.68-1.16(m, 18H); MS (MALDI-TOF) *m/z* calcd for $C_{59}H_{87}N_{15}O_{13}$ 1214.41, found 1214.38 $[M]^+$; HPLC (t_R = 10.1 min, purity = 97.7%, elution

method- 100% H_2O (containing 0.1% TFA) to 100% acetonitrile in 15 minutes on a Supelcosil LC18 column at a flow rate of 2.0 mL/minute).

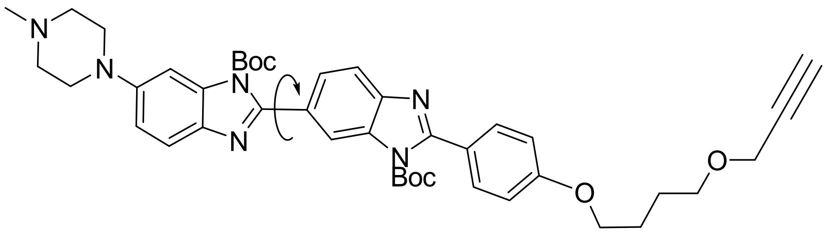

Synthesis of **4b.**

To a solution of **4a** (0.11 g, 0.20 mmol) in dry THF (60.0 mL), NaH was added (0.05 g, 2.08 mmol) at 0 °C. After stirring for 30 min on ice, $(Boc)_2O$ (0.20 g, 0.90 mmol) was added to the solution and the mixture was allowed to slowly warm to room temperature . The stirring was continued overnight following which the reaction mixture was concentrated under the stream of nitrogen. The reaction mixture was mixed with small amount of silica (5 micron mesh size). The crude product was purified by column chromatography on silica gel (5 micron mesh size) using dichloromethane-methanol (0-12 % methanol in dichloromethane) as eluent to obtain the desired product which eluted as two rotamers (rotamer I, 25.2 mg, 17 % and rotamer II, 18.1 mg, 19 %) and a mixture of rotamer I and rotamer II (64.8 mg, 42%)} : R_f = (0.61 for rotamer I and 0.54 for rotamer II respectively in dichloromethane:ethanol 8.5:1.5 *v/v*); ^{1}H NMR (500 MHz, methanol-d_4) (rotamer I) δ 8.37-8.20 (two sets of doublets, 8.37 (d, *J* = 1.43 Hz) and 8.20 (d, *J* = 8.61 Hz), 1H), 7.97-7.83 (two sets of doublets, 7.97 (d, *J* = 1.46 Hz) and 7.84 (d, *J* = 8.37 Hz), 1H), 7.72-7.62 (m, 5H), 7.21 (dd, J_1 = 8.86 Hz , J_2 = 2.40 Hz, 1H), 7.11 (dd, J_1 = 8.85 Hz, J_2 = 1.19 Hz, 2H), 4.19 (d, *J* = 2.39 Hz, 2H), 4.15 (t, *J* = 6.22 Hz, 2H), 3.64 (t, *J* = 6.45 Hz, 2H), 3.34 (some peaks masked by residual MeOH peak from the NMR solvent), 2.87 (t, *J* = 2.39 Hz, 1H), 2.80 (br, 4H) 2.47 (s, 3H) , 1.95- 1.91 (m, 2H), 1.84-1.79 (m, 2H) 1.48-1.36 (18H); MS (MALDI-TOF) *m/z* calcd for $C_{42}H_{50}N_6O_6$ 734.37, found 734.20 $[M]^+$; (rotamer II) ^{1}H NMR (500 MHz, methanol-d_4) δ 8.39-8.20 (two sets of doublets, 8.39 (d, *J* = 1.42 Hz) and 8.21 (d, *J* = 8.52 Hz), 1H) 7.99-7.83 (two sets of doublets 7.98 (d, *J* = 8.98 Hz), 7.84 (d, *J* = 8.15 Hz) 2H) , 7.72 (dd, J_1 = 8.39 Hz, Hz, J_2 = 1.53 Hz, 1H), 7.66-7.64 (m, 2H), 7.28-7.20 (m, 2H), 7.12-7.10 (dd, J_1 = 8.75 Hz, J_2 = 1.78 Hz, 2H), 4.19 (d, *J* = 2.25 Hz, 2H), 4.13 (d, *J* = 6.27 Hz, 2H), 3.65-3.62 (t, *J* =

6.27 Hz, 2H), 3.29 (br, 4H), 2.87 (t, J = 2.24 Hz, 1H), 2.69 (t, J = 4.73 Hz, 4H), 2.39 (s, 3H), 1.95-1.90 (m, 2H), 1.84-1.98 (m, 2H), 1.48-1.40 (18H); MS (MALDI-TOF) *m/z* calcd for $C_{42}H_{50}N_6O_6$ 734.37 found 734.17 $[M]^+$.

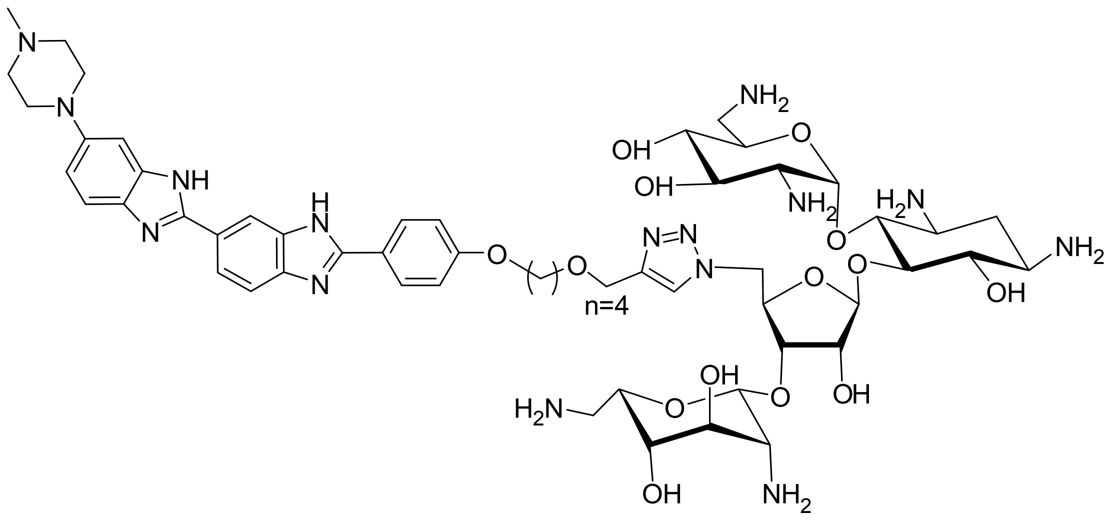

Synthesis of **4**.

To a solution of **4b** (rotamer I, 25.2 mg, 34.0 μmol) in ethanol (3.00 mL), a solution of Boc protected neomycin-azide (42.1 mg, 34.1 μmol) in ethanol (1.00 mL) was added followed by addition of a freshly made copper(I) catalyst made by adding aqueous solutions of $CuSO_4$ (1.60 mg, 10.0 μmol) and sodium ascorbate (3.90 mg, 20.0 μmol) in water (1.00 mL). The reaction mixture was stirred for 24h at room temperature. TLC on silica gel indicated formation of the product. Solvents were evaporated under reduced pressure. The crude product was purified by column chromatography on silica gel (5 micron, mesh size) using dichloromethane-ethanol (0-12 % ethanol in dichloromethane) as eluent. This yielded Boc protected **4** (39.5 mg, R_f = 0.46 in dichloromethane: methanol 9:1 *v/v*). This product (34.5 mg) was then dissolved in dichloromethane (3.00 mL) followed by addition of trifluoroacetic acid (0.60 mL). The mixture was stirred overnight at room temperature. Water (3.00 mL) was added to it which was followed by washing with dichloromethane (3 × 1 mL). The aqueous layer was lyophilized to afford the desired compound as yellow solid (25.2 mg, 39 % for two steps): UV λ_{max} = 337 nm in water; ^1H NMR (500 MHz, D_2O) δ 8.11 (s, br, 1H), 8.04 (s, 1H), 7.94-7.83 (m, 4H), 7.58 (d, J = 9.00 Hz, 1H), 7.22 (dd, J_1 = 9.15 Hz, J_2 = 1.60 Hz, 1H), 7.16 (br, 1H), 7.05 (d, J = 8.5 Hz, 2H), 5.94 (d, J = 3.78 Hz, 1H), 5.32 (d, J = 3.19 Hz, 1H), 5.21 (d, J = 1.30 Hz, 1H), 4.58 (s, br, 2H), 4.47-4.44 (m, 1H), 4.35 (t, J = 4.94 Hz, 1H), 4.21 (t, J = 5.08 Hz, 1H), 4.12 (t, J = 2.90 Hz, 1H), 4.00-3.90 (m, 4H),

3.87-3.73 (m, 6H), 3.58-3.05 (m , 18H), 2.86 (s, 3H), 2.40-2.36 (m, 1H), 1.75-1.62 (m, 4H) (some protons signals are masked by the residual HDO peak from the NMR solvent); MS (MALDI-TOF) *m/z* calcd for $C_{55}H_{79}N_{15}O_{14}$ 1173.59 , found 1175.01[M+H]$^+$; HPLC (t_R = 5.96 min, purity = 98.3%, elution method- 100% H_2O (containing 0.1% TFA) to 100% acetonitrile in 15 minutes on a Supelcosil LC18 column at a flow rate of 2.0 mL/minute).

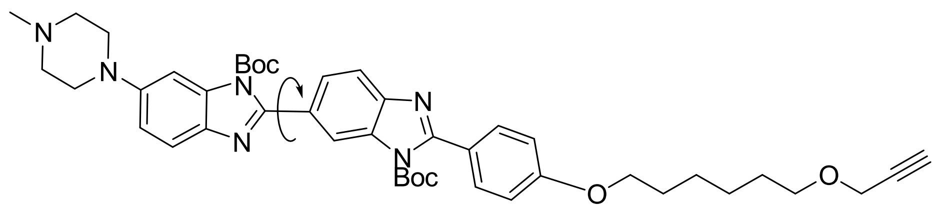

Synthesis of **5b.**

To a solution of **5a** (0.10 g, 0.18 mmol) in dry THF (50.0 mL), NaH was added (12.0 mg, 0.50 mmol) at 0 ^{o}C. After stirring for 15 min, $(Boc)_2O$ (0.23 g, 1.08 mmol) was added to the solution and the mixture was allowed to slowly warm to room temperature . After overnight stirring, the reaction mixture was concentrated under stream of nitrogen. A small amount of silica powder (5 micron mesh size) was added into it. The crude product was purified by column chromatography on silica gel using dichloromethane-ethanol (0-15% ethanol in dichloromethane) as eluent to obtain the desired product which eluted as two different rotamers (rotamer I 38 mg, 28% and rotamer II 46.6 mg, 34%) : R_f = (0.71 for rotamer I and 0.50 for rotamer II respectively in dichloromethane:ethanol 8:2 *v/v*); ^{1}H NMR (500 MHz, methanol-d_4) (rotamer I) δ 8.37-8.20 (set of two doublets 8.36 (d, *J* = 1.03 Hz), 8.21(d, *J* = 8.54 Hz), 1H), 7.98-7.83 (two sets of doublets 7.97 (d, *J* = 1.38 Hz), 7.84 (d, *J* = 8.10 Hz), 1H}, 7.71-7.61 (m, 5H), 7.19 (dd, J_1 = 8.89 Hz , J_2 = 2.12 Hz, 1H), 7.10 (dd, J_1 = 8.73 Hz, J_2 = 2.28 Hz, 2H), 4.16 (d, *J* = 2.28 Hz, 2H), 4.11 (t, *J* = 6.30 Hz, 2H), 3.56 (t, *J* = 6.45 Hz, 2H), 2.85 (t, *J* = 2.21 Hz, 1H), 2.71 (t, *J* = 4.72 Hz, 4H), 2.41 (s, 3H) , 1.88- 1.83 (m, 2H), 1.68-1.62 (m, 2H), 1.59-1.53 (m, 2H), 1.51-1.39 (m, 20H) (some proton signals are masked by the NMR solvent peak); ^{13}C NMR (75 MHz, methanol-d_4) δ 160.9, 155.7, 152.5, 150.1, 148.4, 148.2, 142.5, 135.7, 134.7, 133.2, 130.7, 128.7, 125.8, 123.3, 119.0, 118.3, 115.6, 113.9, 101.4, 86.0, 85.4, 79.4, 74.1, 69.5, 67.8, 57.3, 54.6, 49.5, 44.6, 29.0, 28.8, 26.4, 26.3, 25.5 (two peaks), 25.4; MS (MALDI-TOF) *m/z* calcd for $C_{44}H_{54}N_6O_6$ 762.41.09 found 762.23 [M]$^+$. (rotamer II) ^{1}H NMR (500 MHz, methanol-d_4) δ 8.39-8.21 (two sets of

doublets, 8.39 (d, J = 1.1 Hz) and 8.21 (d, J = 8.94 Hz), 1H), (two sets of doublets 7.98 (d, J = 8.72 Hz, 1H) and 7.84 (d, J = 8.04 Hz) , 2H), 7.72 (dd, J_1 = 1.56 Hz, Hz, J_2 = 8.05 Hz, 1H), 7.65 (m, 2H), 7.28-7.20 (m, 2H), 7.10 (m, 2H), 4.16 (d, J = 1.75 Hz, 2H), 4.10 (t, J = 6.26 Hz, 2H), 3.56 (t, J = 6.26 Hz, 2H), 3.29 (br, 4H), 2.85 (t, J = 2.46 Hz, 1H), 2.70 (t, J = 4.47 Hz, 4H), 2.40 (s, 3H), 1.88-1.82 (m, 2H), 1.68-1.62 (m , 2H), 1.59-1.53 (m, 2H), 1.51-1.39 (m, 20H); ^{13}C NMR (125 MHz, methanol-d$_4$) δ 160.9, 155.8, 154.0, 149.5, 148.2, 142.7, 141.2, 134.6, 130.6, 128.4, 127.9, 126.0, 123.4, 120.4, 118.3, 115.8, 114.8, 114.0, 105.4, 86.0, 85.5, 79.4, 74.1, 69.5, 67.8, 63.3, 57.3, 54.6, 49.6, 44.6, 28.7, 26.4 (two peaks), 25.5, 25.4, 23.8; MS MALDI-TOF *m/z* calcd for $C_{44}H_{54}N_6O_6$ 762.41.09 found 762.51 $[M]^+$.

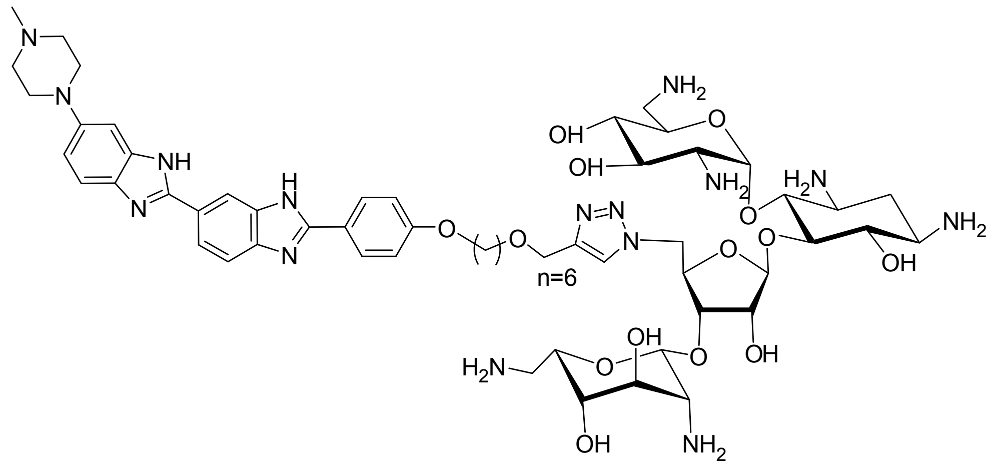

Synthesis of **5**.

To a solution of **5b** (rotamer I, 27.9 mg, 36.5 μmol) in ethanol (3.00 mL), a solution of Boc protected neomycin-azide (45.3 mg, 36.5 μmol) in ethanol (1.00 mL) was added followed by addition of a freshly made copper(I) catalyst made by adding aqueous solutions of $CuSO_4$ (1.6 mg, 10.0 μmol) and sodium ascorbate (3.9 mg, 20.0 μmol). The reaction mixture was stirred for 24h at room temperature. TLC on silica gel indicated formation of the product. Volatiles were evaporated under reduced pressure. The crude product was purified by column chromatography on silica gel (5 micron, mesh

size) using dichloromethane-ethanol as eluent. This yielded Boc protected **5** (49.1 mg, R_f = 0.33 in dichloromethane: methanol 9:1 *v/v*). This product (46.5 mg) was then dissolved in dichloromethane (2.00 mL) followed by addition of trifluoroacetic acid (0.60 mL). The mixture was stirred overnight at room temperature. Water (3.0 mL) was added to it followed which was followed by washing with dichloromethane (3 × 1 mL). The aqueous layer was lyophilized to afford the desired compound as yellow solid (32 mg, 40 % cumulative yield for two steps): UV λ_{max} = 338 nm in water; ^{1}H NMR (500 MHz, D_2O) δ 8.17 (s, br, 1H), 8.03 (s, 1H), 7.96-7.85 (m, 4H), 7.62 (d, *J* = 9.06 Hz, 1H), 7.27 (d, *J* = 9.54, 1H), 7.23 (s, br, 1H), 7.07 (d, *J* = 7.6 Hz, 2H), 5.94 (d, *J* = 3.82 Hz, 1H), 5.32 (d, *J* = 2.86 Hz, 1H), 5.21 (s, br, 1H), 4.56 (s, br, 2H), 4.49-4.44 (m, 1H), 4.34 (t, *J* = 3.34 Hz, 1H), 4.21 (t, *J* = 3.81 Hz, 1H), 4.12 (t, *J* = 3.10 Hz,1H), 4.00-3.90 (m, 4H), 3.88-3.72 (m, 6H), 3.63-3.07 (m, 18H) 2.88 (s, 3H), 2.40-2.37 (m, 1H), 1.82-1.64 (m, 3H), 1.57-1.52 (m, 2H), 1.41-1.27 (m, 4H) (some proton signals are masked by the NMR solvent peak); MS (MALDI-TOF) *m/z* calcd for $C_{57}H_{83}N_{15}O_{14}$ 1201.62, found 1202.69; HPLC (t_R = 7.1 min, purity = 99.8%, elution method- 100% H_2O (containing 0.1% TFA) to 100% acetonitrile in 15 minutes on a Supelcosil LC18 column at a flow rate of 2.0 mL/minute).

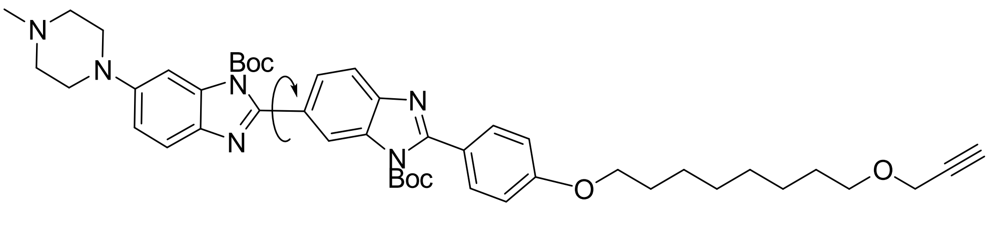

Synthesis of **6b.**

To a solution of **6a** (0.10 g, 0.16 mmol) in dry THF (50.0 mL), NaH was added (12.0 mg, 0.50 mmol) at 0 °C. After 15 min stirring, $(Boc)_2O$ (0.11 g, 0.50) was added to the solution and the mixture was allowed to slowly warm to room temperature . After 5h, the reaction mixture was concentrated under the stream of nitrogen gas. Small amount of silica powder (5 micron mesh size) was added to it. The crude product was purified by column chromatography using dichloromethane-methanol (0-15% ethanol in dichloromethane) as eluent to obtain the desired product which eluted as two different rotamers (rotamer I 37 mg, 28% and a mixture of rotamerI and II 74 mg, 56 %): R_f = (0.73 for rotamer I and 0.47 for rotamer II respectively in dichloromethane:ethanol 8:2 *v/v*); ^{1}H NMR (500

MHz, methanol-d_4) (rotamer I) δ 8.37-8.21 {set of two doublets 8.37 (d, *J* = 1.27 Hz), 8.21(d, *J* = 8.62 Hz), 1H}, 7.98-7.83 {two sets of doublets 7.97 (d, *J* = 1.42 Hz), 7.84 (d, *J* = 8.37 Hz), 1H}, 7.72-7.61 (m, 5H), 7.20 (dd, J_1 = 8.49 Hz , J_2 = 2.21 Hz, 1H), 7.11 (dd, J_1 = 8.96 Hz, J_2 = 1.98 Hz, 2H), 4.15 (d, *J* = 2.21 Hz, 2H), 4.10 (t, *J* = 6.17 Hz, 2H), 3.54 (t, *J* = 6.51 Hz, 2H), 2.85 (t, *J* = 2.21 Hz, 1H), 2.72-2.70 (t, *J* = 4.65 Hz, 4H), 2.41 (s, 3H) , 1.88- 1.82 (m, 2H), 1.64-1.59 (m, 2H), 1.57-1.51 (m, 2H)1.48-1.36 (m, 22H); MS (MALDI-TOF) *m/z* calcd for $C_{46}H_{58}N_6O_6$ 790.44 found 790.58 $[M]^+$.

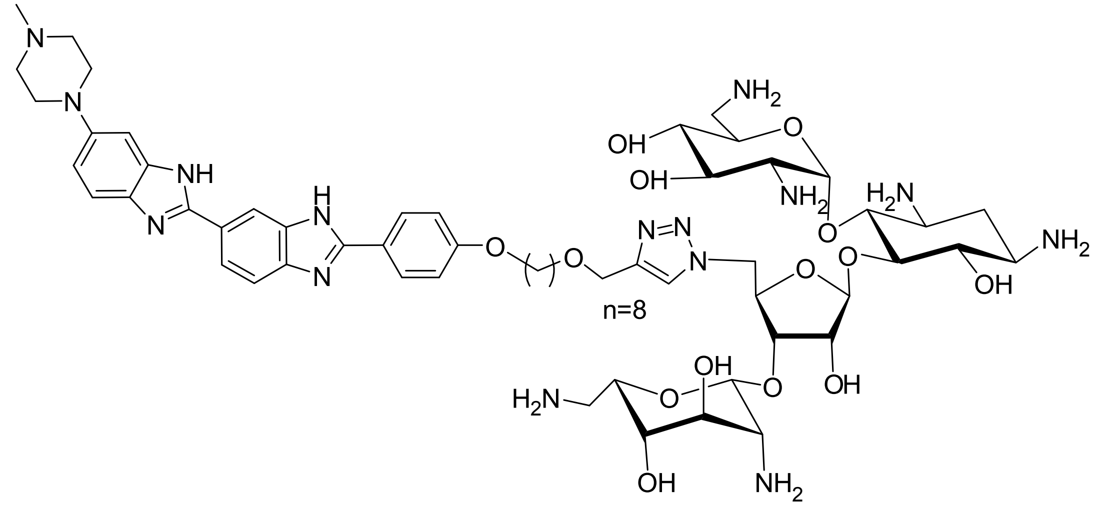

Synthesis of **6**.

To a solution of **6b** (rotamer I, 27.0 mg, 34.1 μmol) in ethanol (3.00 mL), a solution of Boc protected neomycin-azide (42.3 mg, 34.1 μmol) in ethanol (1.00 mL) was added followed by addition of a freshly made copper(I) catalyst made by adding aqueous solutions of $CuSO_4$ (1.6 mg, 10.0 μmol) and sodium ascorbate (3.9 mg, 20.0 μmol). The reaction mixture was stirred for 24h at room temperature. TLC on silica gel indicated formation of the product. Volatiles were evaporated under reduced pressure. The crude product was purified by column chromatography on silica gel (5 micron, mesh size) using dichloromethane-ethanol (0-12 % ethanol in dichloromethane) as eluent. This yielded Boc protected **6** (49.1 mg, R_f = 0.33 in dichloromethane: methanol 9:1 *v/v*). This product (46.5 mg) was then dissolved in dichloromethane (2.00 mL) followed by addition of trifluoroacetic acid (0.60 mL). The mixture was stirred overnight at room temperature. Water (3.0 mL) was added to it which was followed by washing with dichloromethane (3 × 1 mL). The aqueous layer was lyophilized to afford the desired compound as yellow solid (35 mg, 48 % for two steps): UV λ_{max} = 338 nm in water; ^{1}H NMR (500 MHz, D_2O) δ 8.18 (s, br, 1H), 8.03 (s, 1H), 7.97-7.86 (m, 4H), 7.64 (d, *J* = 9.15 Hz, 1H),

7.28 (d, $J = 9.15$, 1H), 7.24 (s, br, 1H), 7.07 (d, $J = 7.8$ Hz, 2H), 5.94 (d, $J = 4.20$ Hz, 1H), 5.32 (d, $J = 2.94$ Hz, 1H), 5.21 (d, $J = 1.20$ Hz, 1H), 4.55 (s, br, 2H), 4.48 (m, 1H), 4.35-4.34 (t, $J = 4.86$ Hz, 1H), 4.22-4.20 (t, $J = 4.86$ Hz, 1H), 4.13-4.11 (t, $J = 3.10$ Hz,1H), 4.00-3.88 (m, 4H), 3.88-3.77 (m, 4H), 3.74 (s, br, 2H), 3.60-3.42 (m, 9H), 3.38-3.09 (m, 9H) 2.88 (s, 3H), 2.40-2.37 (m, 1H), 1.82-1.74 (m, 1H), 1.71-1.63 (m, 2H), 1.53-1.48 (m, 2H), 1.36-1.20 (m, 8H) (some proton signals are masked by the NMR solvent peak); MS (MALDI-TOF) *m/z* calcd for $C_{59}H_{87}N_{15}O_{14}$ 1229.65 , found 1230.83 $[M+H]^+$; HPLC ($t_R = 9.1$ min, purity = 99.2%, elution method- 100% H_2O (containing 0.1% TFA) to 100% acetonitrile in 15 minutes on a Supelcosil LC18 column at a flow rate of 2.0 mL/minute).

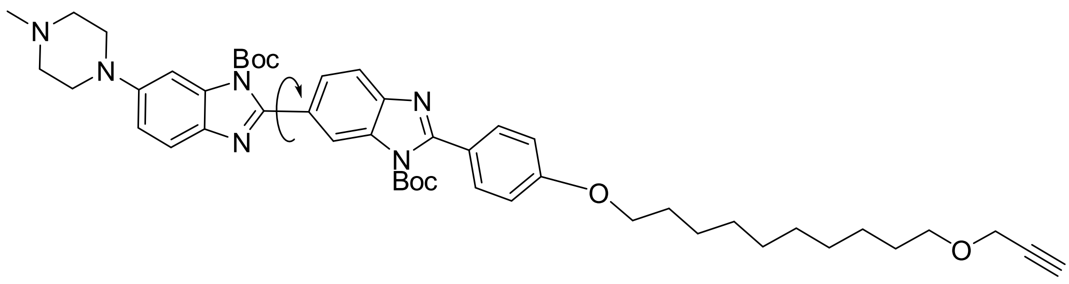

Synthesis of **7b**.

To a solution of **7a** (0.10 g, 0.16 mmol) in dry THF (40.0 mL), NaH was added (0.10 g, 4.16 mmol) at 0 ^oC. After 15 min stirring, $(Boc)_2O$ (0.20 g, 0.91 mmol) was added to the solution and the mixture was allowed to slowly warm to room temperature . After 5h, the reaction mixture was concentrated under the stream of nitrogen gas and mixed with small amount of silica powder (5 micron mesh size). The crude mixture was purified by column chromatography on silica gel (5 micron mesh size) using dichloromethane-ethanol (0-15% ethanol in dichloromethane) as eluent to obtain the desired product which eluted as two different rotamers (rotamer I, 49.3 mg, 37 % and rotamer II 41 mg, 31 %) and a mixture of rotamer I and rotamer II (33 mg, 25%): R_f = (0.28 for rotamer I and 0.14 for rotamer II respectively in dichloromethane:ethanol 9:1 *v/v*); ^{1}H NMR (300 MHz, methanol-d_4) (rotamer I) δ 8.36-8.18 (two sets of doublets, 8.36 (d, $J = 1.0$ Hz) and 8.21 (d, $J = 8.95$ Hz), 1H), 7.96-7.81 (two sets of doublets, 7.86 (d, $J = 1.0$ Hz) and 7.81 (d, $J = 8.30$ Hz), 1H), 7.71-7.59 (m, 5H), 7.18 (dd, $J_1 = 2.16$

Hz , J_2 = 8.79 Hz, 1H), 7.09 (dd, J_1 = 1.99 Hz, J_2 = 8.80 Hz, 2H), 4.13 (d, 2.49 Hz, 2H), 4.09 (t, J = 6.31 Hz, 2H), 3.52 (5, J = 6.64 Hz, 2H), 3.34 (some peaks masked by residual MeOH peak from the NMR solvent), 2.82, (t, J = 2.49 Hz, 1H), 2.71 (t, J = 4.64 Hz, 4H), 2.40 (s, 3H) , 1.86-1.79 (m, 2H), 1.60- 1.30 (m, 32H); ^{13}C NMR (125 MHz, methanol-d_4) δ 160.9, 155.4, 152.4, 150.0, 148.5, 148.1, 142.5, 135.7, 134.7, 133.3, 130.7, 128.7, 126.0, 123.4, 119.8, 119.1, 118.3, 115.7, 114.0, 101.6, 86.0. 85.5, 79.4, 74.0, 69.6, 67.9, 57.3, 54.6, 49.5, 44.6, 29.2, 29.0 (two peaks), 28.8, 26.4(two peaks), 25.8 (two peaks), 25.7, 17.6; MS (MALDI-TOF) *m/z* calcd for $C_{48}H_{62}N_6O_6$ 818.47 found 818.16 $[M]^+$. (rotamer II) ^1H NMR (300 MHz, methanol-d_4) δ 8.38-8.19 (two sets of doublets, 8.38 (d, J = 1.2 Hz, 1H) and 8.20 (d, J = 8.84 Hz), 1H) 7.99-7.82 (m, 2H), 7.73-7.62 (m, 3H), 7.28-7.19 (m, 2H), 7.09 (dd, J_1 = 2.05 Hz , J_2 = 8.81, 2H), 4.14 (d, J = 2.47 Hz, 2H) 4.09 (t, J = 6.26 Hz, 2H), 3.52 (t, J = 6.51 Hz, 2H), 3.29(t, J = 4.35 Hz, 4H), 2.82 (t, J = 2.47 Hz, 1H), 2.69 (t, J = 4.85 Hz, 4H), 2.39 (s, 3H), 1.86-1.78 (m , 2H), 1.61-1.33 (32H); ^{13}C NMR (125 MHz, methanol-d_4) δ 161.0, 155.8, 153.9, 149.5, 148.2, 142.6, 141.2, 134.6, 133.3, 130.7, 128.4, 126.0, 123.4, 120.0, 118.3, 116.3, 115.8, 114.8, 114.0, 105.4, 86.0, 85.5, 83.6, 79.4, 74.0, 69.6, 67.9, 57.3, 54.6, 49.6, 44.6, 29.3, 29.2, 29.1, 29.0 (two peaks), 28.8, 26.4 (two peaks), 25.7 two peaks); MS (MALDI-TOF) *m/z* $C_{48}H_{62}N_6O_6$ 818.47 found 818.16 $[M]^+$.

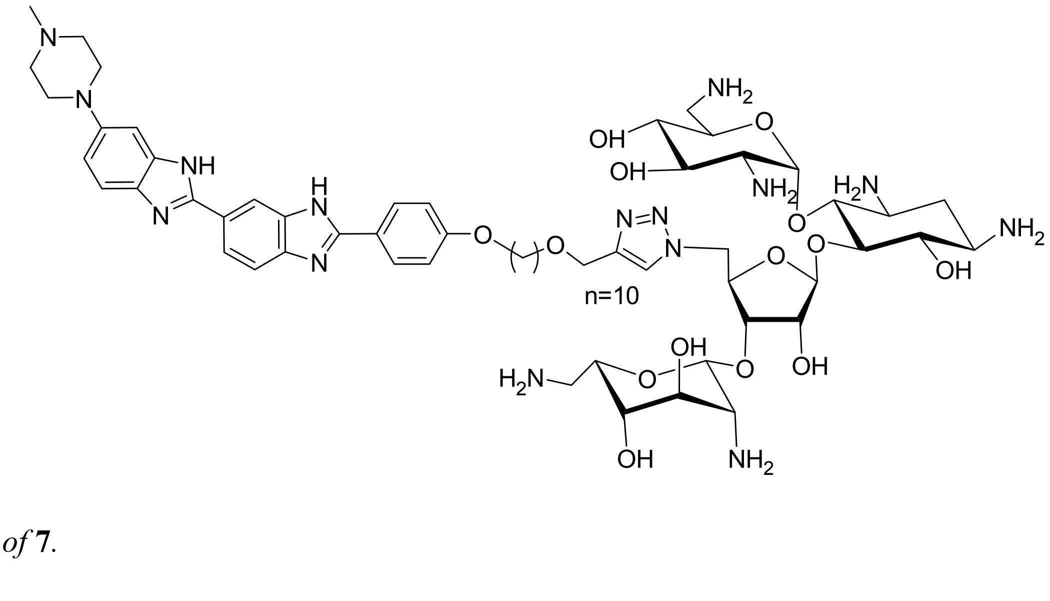

Synthesis of **7**.

To a solution of **7b** (rotamer I, 39.2 mg, 47.9 μmol) in ethanol (3.00 mL), a solution of Boc protected neomycin-azide (60.0 mg, 47.9 μmol) in ethanol (1.00 mL) was added followed by addition of a

freshly made copper(I) catalyst made by adding aqueous solutions of $CuSO_4$ (1.75 mg, 11.0 μmol) and sodium ascorbate (4.35 mg, 22.0 μmol) in water (0.50 mL). The reaction mixture was stirred for 24h at room temperature. TLC on silica gel indicated formation of the product. Solvents were evaporated under reduced pressure. The crude mixture was purified using column chromatography on silica gel (5 micron, mesh size) using dichloromethane-ethanol as eluent. This yielded Boc protected **7** (41.1 mg, R_f = 0.36 in dichloromethane: ethanol 9:1 *v/v*). The product was dissolved in dichloromethane (3.00 mL) followed by addition of trifluoroacetic acid (0.60 mL). The mixture was stirred for 2h at room temperature. Water (3.00 mL) was added to it which was followed by washing it with dichloromethane (3×1 mL). The aqueous layer was lyophilized to afford the desired compound as pale yellow solid (32 mg, 29 % for two steps): UV λ_{max} = 338 nm in water; ^1H NMR (500 MHz, D_2O) δ 8.16 (s, br, 1H), 8.03 (s, br, 1H), 7.93-7.85 (m, 4H), 7.65 (d, *J* = 9.55 Hz, 1H), 7.28-7.24 (m, 2H), 7.05 (s, br, 2H), 5.95 (s, br, 1H), 5.33 (s,b r, 2H), 5.21 (s, br, 1H), 4.54 (s, br, 2H), 4.47 (s, br, 1H), 4.35 (s, br, 1H), 4.22 (s, br, 1H), 4.12 (s, br, 1H), 3.99-3.74 (m, 10H), 3.61-3.09 (m, 20H), 2.88 (s, 3H), 2.40-2.37 (m, 1H), 1.80-1.77 (m, 1H), 1.66 (s, br, 2H), 1.50 (s, br, 2H), 1.37-1.29 (m, 4H), 1.23-1.21 (m, 14H); MS (MALDI-TOF) *m/z* calcd for $C_{61}H_{91}N_{15}O_{14}$ 1257.68 , found 1258.77 $[M+H]^+$; $]^+$; HPLC (t_R = 9.5 min, purity = 98.4%, elution method- 100% H_2O (containing 0.1% TFA) to 100% acetonitrile in 15 minutes on a Supelcosil LC18 column at a flow rate of 2.0 mL/minute).

Synthesis of Neomycin- monobenzimidazole conjugates **(8-12)**

The synthesis of clickable monbenzimidazole linkers (**17-21**) is outlined in scheme S1 and the synthesis of neomycin-monobenimidazole conjugates (**8-12**) is shown in schemes S2-3. The synthesis of neomycin-monobenzimidazole conjugates starts with the synthesis of linkers **17-21**. Commercially available 5- chloro, 2-nitro aniline was reacted with N-methyl piperazine under basic conditions to give compound 5-(4-methylpiperazin-1-yl)-2-nitroaniline which upon reduction gave **14**. [37] The diamine **14** was then reacted with 4-(prop-2-ynyloxy)benzaldehyde or 4-(2-azidoethoxy)benzaldehyde [38] to give alkyne (**15**) [32] or azido ended benzimidazoles (**16**) respectively suitable for applications in click chemistry. From these starting compounds, the benzimidazole linkers (**17-19**) terminating in either azido (by reacting compound **15** with excess bisazides) or alkyne ended benzimidazole derivatives (by reacting compound **16** with excess bisalkynes) were prepared under click chemistry conditions.[39] The benzimidazole linkers (**17-21**) were conjugated with appropriate Boc protected neomycin derivatives terminating in azide (**22**) (SchemeS3) or alkyne (**23**) (Scheme S2) to give Boc

protected neomycin-benzimidazole conjugates which upon treatment with acid (4N HCl) gave the desired conjugates **8-12**.

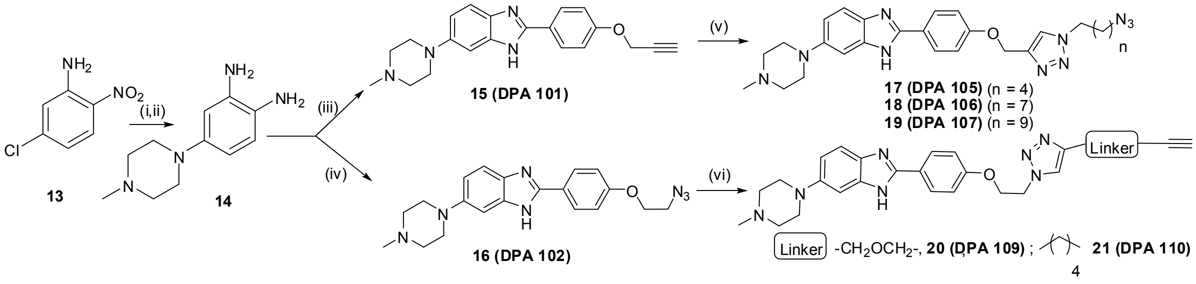

Scheme S1. Reagents and conditions (i) DMF, N-methylpiperazine, 100 °C, 5h, 60% (ii) Pd-C (10%), ethanol, H_2, 6h, qaunt. (iii) 4-(prop-2-ynyloxy) benzaldehyde, $Na_2S_2O_5$, H_2O, reflux, 12h, 65% (iv) 4-(2-azidoethoxy) benzaldehyde, $Na_2S_2O_5$, H_2O, reflux, 12h, 82% (v) sodium ascorbate, copper (II) sulfate, 50 fold excess bisazides [N_3-CH_2-$(CH_2)_n$-N_3; n = 6 (**17**); n = 8 (**18**), n = 9 (**19**)], ethanol, room temperature, overnight, 65-80 % (vi) sodium ascorbate, copper (II) sulfate, 50 fold excess bisalkynes [propargyl ether (**20**), HC-$(CH_2)_n$-CH; n = 6(**21**)] ethanol, rt, overnight, 68-75 %.

Synthesis of conjugates 8-10.

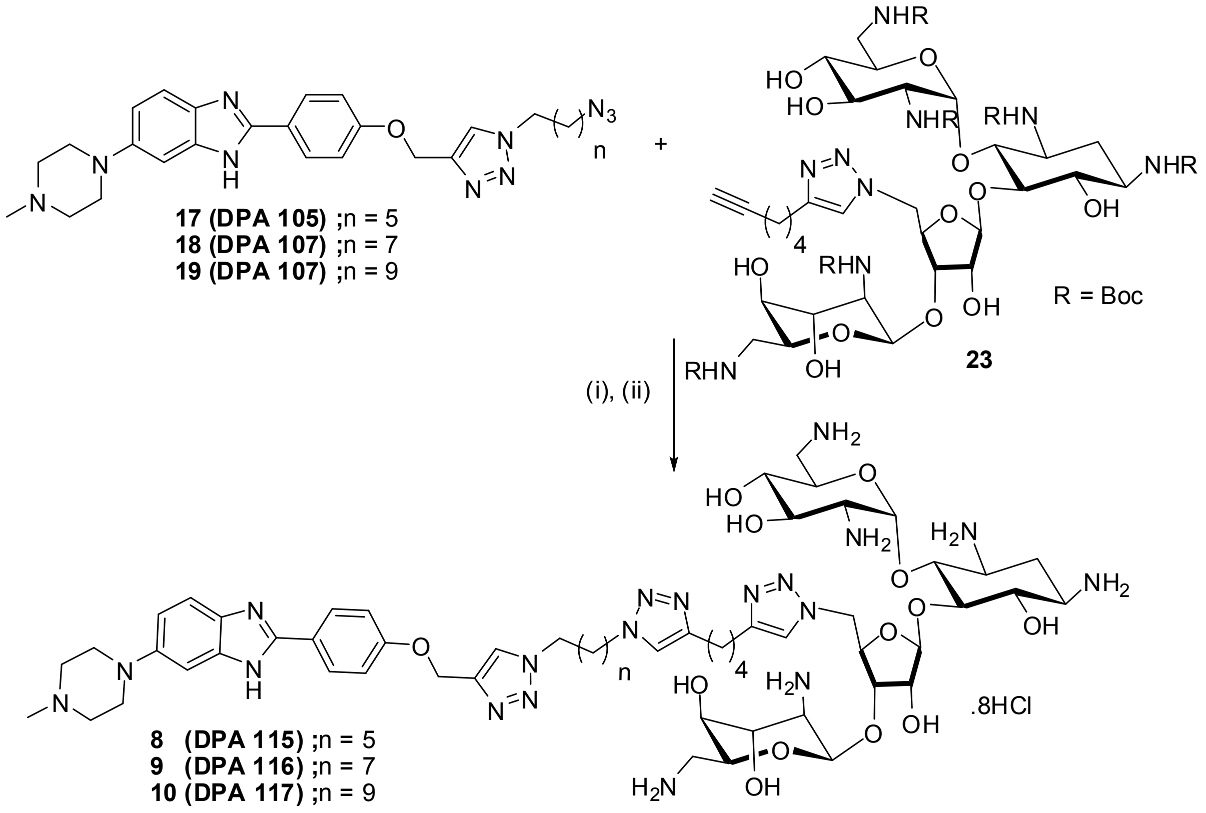

Scheme S2. (i) sodium ascorbate, copper (II) sulfate, ethanol, room temperature, overnight (ii) dichloromethane, 4M HCl in 1,4 dioxane, 52-56 % cumulative yield for two steps

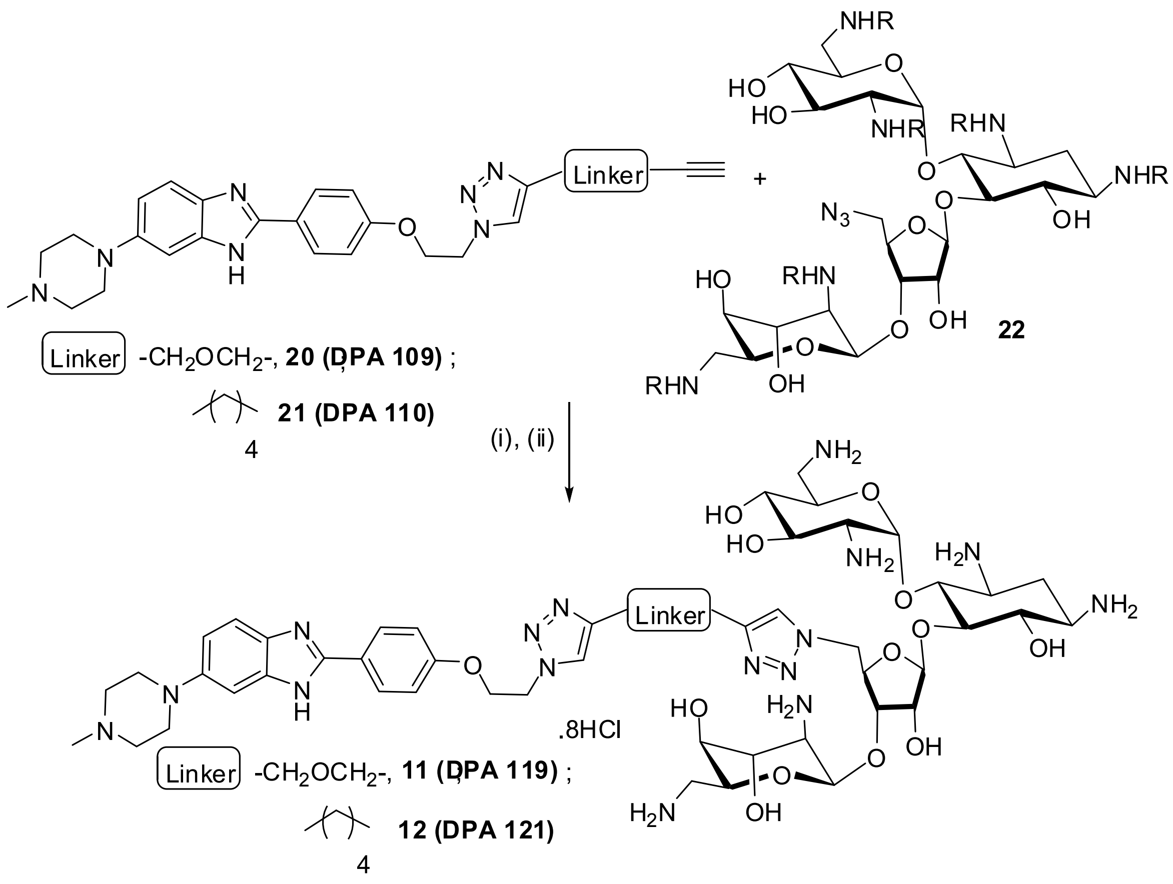

Scheme S3. A scheme for the synthesis of neomycin-monobenzimidazole conjugates **11, 12** (i) sodium ascorbate, copper (II) sulfate, ethanol, room temperature, overnight (ii) dichloromethane, 4M HCl in 1,4 dioxane, 66-70 % cumulative yield for two steps .

Synthesis of compound 15. Synthesis of compound **15** has been reported previously.[1]

Synthesis of compound 16.

To a solution of 5-(4-methylpiperazin-1-yl)-2-nitroaniline (400 mg, 1.69 mmol) in ethanol-ethyl acetate (2:1 *v/v*) mixture (15 mL), 10% Pd-C (200 mg) was added and the mixture was hydrogenated for 4h which afforded the corresponding diamine. Charcoal was filtered off. To this diamine, 4-(2-azidoethoxy) benzaldehyde [2] (413 mg, 2 .16 mmol) and sodium pyrosulfite (321 mg, 1.69 mmol) in water (0.5 mL) was added and then the contents were refluxed overnight. The volatiles were removed under reduced pressure. The crude mixture was purified on a silica gel column using dichloromethane-methanol as eluent to afford the desired compound as off white solid (0.53 g, 82 %): R_f = 0.53 (dichloromethane-methanol 8:2 v/v); IR (neat) cm^{-1} 2937, 2798, 2103, 1585, 1437, 1282, 959; ^1H NMR (500 MHz, methanol-d_4) δ 8.02 (d, J = 8.88 Hz, 2H), 7.50 (d, J = 8.77 Hz, 1H), 7.14-7.11 (m, 3H), 7.06 (dd, J_1 = 8.88 Hz, J_2 = 2.06 Hz, 1H), 4.26 (t, J = 4.76 Hz, 2H), 3.65 (t, J = 4.87 Hz, 2H), 3.25 (t, J = 4.55 Hz, 4H), 2.72 (t, J = 4.65 Hz, 4H), 2.42 (s, 3H); ^{13}C NMR (125 MHz, methanol-d_4) δ 158.5, 150.0, 146.6, 126.2, 121.3, 113.4, 113.2, 65.6, 53.2, 48.8, 48.4, 43.1; MS (MALDI-TOF) *m/z* calcd for $C_{20}H_{23}N_7O$ 377.19, found 377.07 $[M]^+$.

General scheme of synthesis for compounds 17-19.

To a solution of **15** (100 mg, 0.29 mmol) in a mixture of methanol-water (3 mL, 2:1 *v/v*), appropriate diazide (50 fold excess) was added followed by the addition of a freshly prepared copper catalyst {copper sulfate (7 mg, 0.043 mmol), sodium ascorbate (20 mg, 0.10 mmol)}. The mixture was stirred overnight at room temperature. Volatiles were removed under reduced pressure. Crude product was purified by column chromatography using dichloromethane-methanol (0-15% methanol in dichloromethane) as eluent to afford the desired compounds as pale yellow powder.

Note: The carbon spectra of benzimidazoles **17-21** do not give all the expected signals of carbons as observed previously.[3]

Compound **17**: (Yield = 65%); R_f = 0.45 (dichloromethane-methanol 8:2 *v/v*); ^1H NMR (500 MHz, methanol-d_4) δ 8.11 (s, 1H), 8.02-8.01 (d, J = 8.39 Hz, 2H), 7.51-7.50 (d, J = 8.64 Hz, 1H), 7.19-7.17 (d, J = 8.39 Hz, 2H), 7.15 (s, br, 1H), 7.07-7.05 (dd, J_1 = 8.88 Hz, J_2 = 1.97 Hz, 1H), 5.27 (s, br, 2H), 4.45-4.42 (t, J = 6.91 Hz, 2H), 3.29-3.23 (m, 6H), 2.89 (br, 4H), 2.55 (s, 3H), 1.96-1.90 (m, 2H), 1.58-1.52 (m, 2H), 1.44-1.38 (m, 2H), 1.35-1.29 (m, 2H); MS (MALDI-TOF) *m/z* calcd for $C_{27}H_{34}N_{10}O$ 514.29, found 514.17$[M]^+$.

Compound **18**: (Yield = 80%); R_f = 0.39 (dichloromethane-methanol 9:1 *v/v*); IR (neat) 2083 Cm^{-1} (N-N stretch); ^1H NMR (500 MHz, methanol-d_4) δ 8.10 (s, 1H), 8.01 (d, J = 8.4 Hz, 2H), 7.50 (d, J = 8.6 Hz, 1H), 7.30 – 6.99 (m, 4H), 5.27 (s, 2H), 4.43 (t, J = 6.9 Hz, 2H), 3.20(br, 4H), 2.76 (br 4H), 2.45 (s, 2H), 1.88 (m, 2H), 1.65 – 1.12 (m, 12H); ^{13}C NMR (75 MHz, methanol-d_4) δ 159.6, 148.0, 127.7, 124.0, 122.8, 115.1, 115.0, 61.2, 54.6, 50.8, 50.0, 44.5, 29.7, 28.6, 28.4, 28.3, 26.2, 25.9 ; MS (MALDI-TOF) *m/z* calcd for $C_{29}H_{38}N_{10}O$ 542.32, found 542.49 $[M]^+$.

Compound **19**: (Yield = 50%); IR (neat) 2091 Cm^{-1} (N-N stretch); R_f = 0.37 (dichloromethane-methanol 9:1 v/v); ^1H NMR (500 MHz, methanol-d_4) δ 8.11 (s, 1H), 8.02 (d, J = 8.0 Hz, 2H), 7.50 (d, J = 8.9 Hz, 1H), 7.23 – 7.10 (m, 3H), 7.06 (d, J = 8.7 Hz, 1H), 5.29 (s, 2H), 4.43 (t, J = 7.0 Hz, 2H), 3.24 (br, 4H), 2.76 (br, 4H), 2.44 (s, 3H), 1.96 – 1.82 (m, 2H), 1.52 (dd, J = 14.6, 7.1 Hz, 2H), 1.28 (d, J = 26.7 Hz,14H); ^{13}C NMR (125 MHz, methanol-d_4) δ 159.7, 148.1, 127.7, 123.3, 115.1, 100.0, 61.2, 54.7, 51.0, 50.2, 50.0, 44.4, 29.7, 28.9, 28.9, 28.7, 28.5, 28.4, 26.3, 25.9; MS (MALDI-TOF) *m/z* calcd for $C_{31}H_{42}N_{10}O$ 570.35, found 570.60 $[M]^+$.

General scheme of synthesis for compounds 20, 21.

To a solution of **16** (100 mg, 0.26 mmol) in a mixture of methanol-water (3 mL, 2:1 *v/v*), appropriate bisalkyne (50 fold excess) was added followed by the addition of a freshly prepared copper catalyst {copper sulfate (7 mg, 0.043 mmol), sodium ascorbate (20 mg, 0.10 mmol)} in water 1.0 mL). The mixture was stirred overnight at room temperature. Volatiles were removed under reduced pressure. Crude product was purified by column chromatography using dichloromethane-methanol (0-15% methanol in dichloromethane) as eluent to afford the desired compound as pale white powder.

Compound **20**: (Yield 68 %); R_f = 0.41 (dichloromethane-methanol 9:1 v/v); ^1HNMR (500 MHz, methanol-d_4): δ 8.12 (s, 1H), 7.98 (d, J = 8.8 Hz, 2H), 7.50 (d, J = 8.8 Hz, 1H), 7.16-7.15 (d, J = 1.9 Hz, 1H), 7.10-7.04 (m, 3H), 4.88 (t, J = 4.7 Hz, 2H), 4.70(2H), 4.50 (t, J = 5.1 Hz, 2H,), 4.20 (d, J = 2.4 Hz, 2H), 3.33-3.32 (br, 4H), 3.15-3.13 (br, 4H), 2.92-2.89 (t, J = 2.3 Hz, 1H), 2.73 (s, 3H); MS (MALDI-TOF) *m/z* calcd for $C_{26}H_{29}N_7O_2$ 471.23, found 471.35 $[M]^+$.

Compound **21:** (Yield = 75 %); R_f = 0.43 (dichloromethane-methanol 9:1 *v/v*); ^1HNMR (300 MHz, acetone-d_6): δ 8.11 (d, J = 8.8 Hz, 2H), 7.84 (s, 1H), 7.44 (d, J = 8.7 Hz, 1H), 7.09-7.05 (br, 3H), 6.95 (dd, J_1 = 8.7Hz, J_2 = 2.2Hz, 1H), 4.82 (t, J = 5.1Hz, 2H), 4.55-4.52(t, J = 5.1Hz, 2H), 3.20 3.17 (t, J = 4.4Hz, 4H), 2.73 (t, J = 1.93 Hz, 1H), 2.61 (t, J = 4.4Hz, 4H), 2.34-2.33(s,3H), 2.24-2.19 (m,2H),1.83-1.72 (m, 2H), 1.61-1.52 (m, 2H), some NMR signals overlap with the residual NMR solvent signal ; MS (MALDI-TOF) *m/z* calcd for $C_{28}H_{33}N_7O$ 483.27, found 483.47 $[M]^+$.

Synthesis of compound 23.

To a solution of Boc neomycin azide (100 mg, 0.008 mmol) in ethanol (2.0 mL), 1, 7 octadiyne (50 fold excess) was added followed by the addition of $CuSO_4$ (0.2 equivalent) and sodium ascorbate (0.4 equivalent) in water (0.5 mL). After stirring the mixture overnight, the volatiles were removed on rotary evaporator. The mixture was taken up in dichloromethane (50 mL) and washed with water (3× 20 mL). The organic layer was dried with sodium sulphate and followed by removal of volatiles on rotary evaporator to give the desired compound **23** as an inseparable mixture with Boc neomycin azide. The product stains with both KMnO4 and ninhydrin (R_f = 0.44 in dichlromethane-metahnol 9:1) (Compound **23**, where mentioned next, is a mixture together with Boc-neomycin azide)

General scheme for the synthesis of neomycin-benzimidazole conjugates 8-12.

To a solution of benzimidazole azido linkers (**17-19**) or benzimidazole alkyne linkers (**20, 21**) (34.7 μmol) in ethanol (1.0 mL), a solution of $CuSO_4$ (1.59 mg, 10 mmol) and sodium ascorbate (3.96 mg, 20 mmol) in 0.5 mL water was added followed by addition of Boc protected neomycin alkyne (**22**, for benzimidazole linkers **17-19**) or Boc protected neomycin azide (**23**, for benzimidazole linkers **20, 21**) (34.7 mmol assuming 100% alkyne in the mixture). The reaction mixture was stirred at room temperature overnight while being wrapped in aluminum foil. TLC showed formation of the product. Volatiles were evaporated and the crude product was taken up in dichloromethane and then wet loaded on a silica gel column. Elution with dichloromethane-methanol (0-18 % methanol in dichloromethane) afforded the desired Boc protected neomycin-benzimidazole conjuagtes (**8-12a**) as white solid. The Boc protected conjugate was taken up in dichloromethane (3 mL), 4M-HCl in 1, 4 dioxane (0.5 mL) was added to it. After stirring at room temperature for 30 min, precipitation of the conjugate was induced by addition of small volumes (~1-2 mL) hexanes and ether. The precipitated product was centrifuged and the supernatant was discarded. The precipitated solid was taken up in water and extracted with dichloromethane (3×1 mL). The aqueous layer was lyophilized to dryness to afford the desired neomycin conjuagtes (**8-12**) as yellowish white solid (52-68 %) overall yield for two steps.

Note. For clarity in the ^{1}H NMR spectra of compounds **8-12**, the residual proton signals from the deuterared solvent were suppressed.

Compound **8:** ^{1}H NMR (300 MHz , D_2O): δ 8.58 (1H), 8.10 (1H), 7.92-7.64 (2H), 7.58- 7.44 (1H), 7.37 (1H), 7.29- 7.00 (4H), 5.97 (1H), 5.39- 5.08 (3H), 4.55-4.37 (1H), 4.35-4.18 (1H), 4.16-3.68 (7H), 3.66-2.93 (16H), 2.91 (3H), 2.49-2.33 (1H), 1.92-1.32 (5H), 1.23-0.65 (9H); MS (MALDI-TOF) *m/z* calcd for $C_{58}H_{89}N_{19}O_{13}$ 1259.68, found 1260.99 $[M+H]^+$.

Compound **9:** ^{1}H NMR (300 MHz, D_2O): δ 8.63-6.99 (10 H), 5.82 (d, *J* = 6.5 Hz, 1H), 4.40-4.03 (7H), 4.02-3.82 (6H), 3.74-3.27 (24H), 3.15-2.90 (4H), 2.86 (s, 3H), 2.44-2.37 (m, 2H), 2.00-1.47 (10H), 1.31-0.82 (14H); MS (MALDI-TOF) *m/z* calcd for $C_{60}H_{93}N_{19}O_{13}$ 1287.72, found 1289.18 $[M+H]^+$.

Compound **10:** ^{1}H NMR (300 MHz, D_2O): δ 8.61 (1H), 8.44 (1H), 8.00 (1H), 7.83-7.72(2H), 7.69-7.62 (1H), 7.60-7.48 (1H),7.21-7.08 (4H), 5.99 (1H), 5.34 (1H), 5.12 (1H), 4.89-4.80 (1H), 4.73-4.52 (3H), 4.50-4.43(1H), 4.41-4.34 (1H), 4.32-4.20 (4H), 4.18-4.01(5H), 3.98-3.82(6H), 3.79-3.67 (4H), 3.39-3.22 (8H), 3.20-2.96 (9H), 2.97-2.81 (7H), 2.80-2.69 (2H), 2.68-2.48 (3H), 2.46-2.33(2H), 1.98 (1H), 1.95-1.74 (4H), 1.73-1.30 (8H), 1.29-1.03(9H), 1.01-0.67 (14H); MS (MALDI-TOF) *m/z* calcd for $C_{62}H_{97}N_{19}O_{13}$ 1315.75, found 1315.80 $[M]^+$.

Compound **11:** ^{1}H NMR (300MHz , D_2O,): δ 8.09 (1H), 7.90 (1H), 7.69- 7.66 (2H), 7.53-7.42 (1H), 7.22-7.08 (2H), 6.99-6.87 (2H), 5.82-5.81 (1H), 5.37-5.06 (3H), 4.60-4.31 (5H), 4.29-4.02 (6H), 3.99-3.81 (6H), 3.80-3.68 (5H), 3.65-3.22(23H), 3.20-3.01 (16H), 3.00-2.80 (6H), 2.46-2.35 (2H), 1.96-1.74 (2H), 1.16-1.03 (2H); MS (MALDI-TOF) *m/z* calcd for $C_{49}H_{74}N_{16}O_{14}$ 1110.55, found 1111.85 $[M+H]^+$.

Compound **12:** ^{1}H NMR (D_2O, 500MHz): δ 8.17 (s, 1H), 7.98 (s, 1H), 7.67(d, *J* = 8.8 Hz, 2H), 7.42 (d, , *J* = 9.7 Hz, 1H), 7.06 (d, *J* = 7.4 Hz, 2H), 6.96 (d, *J* = 8.8 Hz , 1H), 5.99 (1H), 4.55-4.44 (2H), 4.42- 4.34 (2H), 4.30-4.21 (2H), 4.16-4.05 (2H), 4.00-3.81 (4H), 3.77-3.60 (6H), 3.56-3.14 (12H), 3.14-2.95 (4H), 2.85 (3H), 2.77-2.51 (4H), 2.48-2.34 (1H), 1.86-1.80 (1H), 1.61 (4H); MS (MALDI-TOF) *m/z* calcd for $C_{51}H_{78}N_{16}O_{13}$ 1123.26, found 1123.89 $[M]^+$.

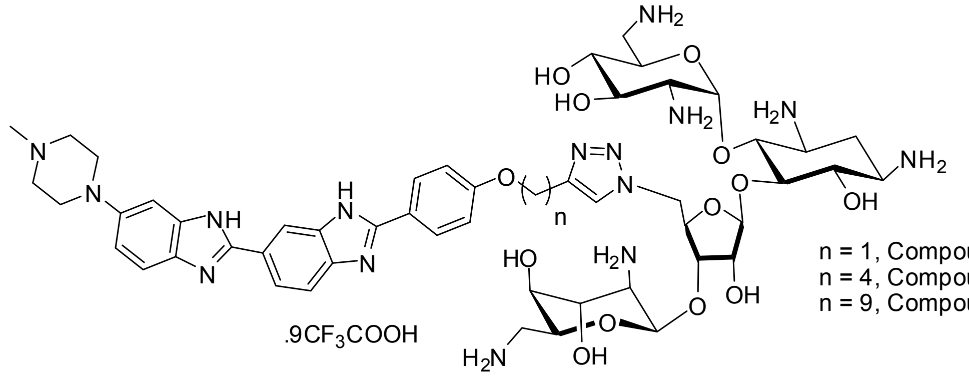

n = 1, Compound **1 (DPA 165)**
n = 4, Compound **2 (DPA 166)**
n = 9, Compound **3 (DPA 167)**

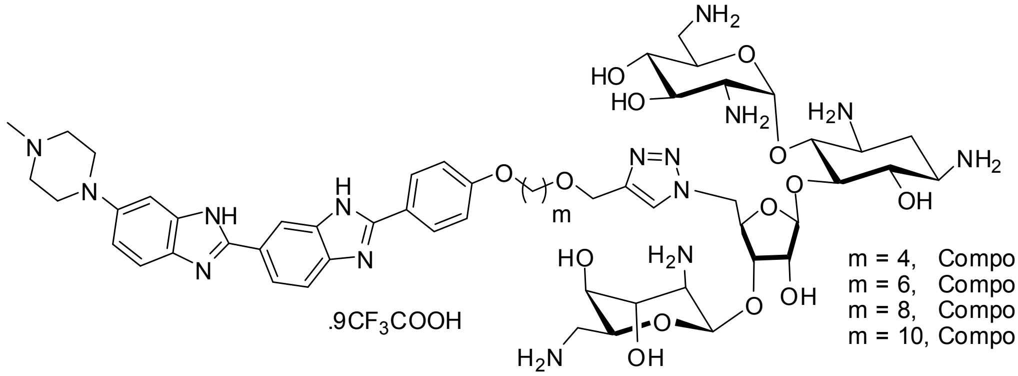

m = 4, Compound **4 (DPA 168)**
m = 6, Compound **5 (DPA 169)**
m = 8, Compound **6 (DPA 170)**
m = 10, Compound **7 (DPA 171)**

Fig **S1**: Structures of neomycin-bisbenzimidazole conjugates **1-7**.

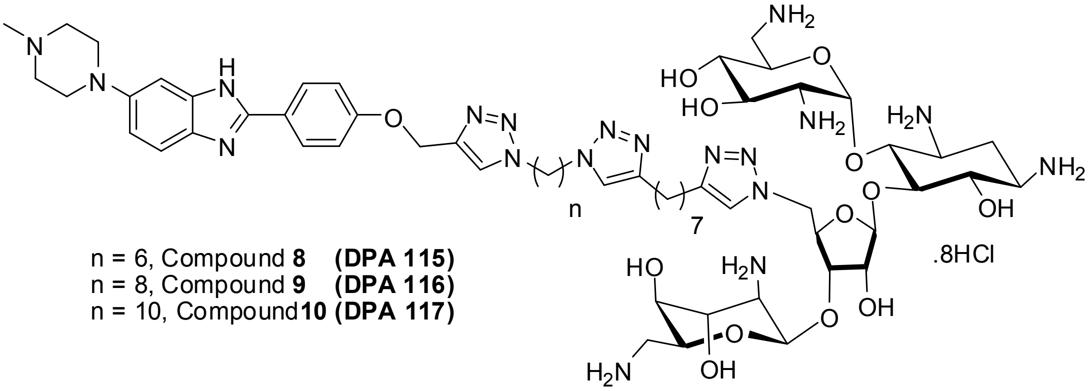

n = 6, Compound **8** **(DPA 115)**
n = 8, Compound **9** **(DPA 116)**
n = 10, Compound **10** **(DPA 117)**

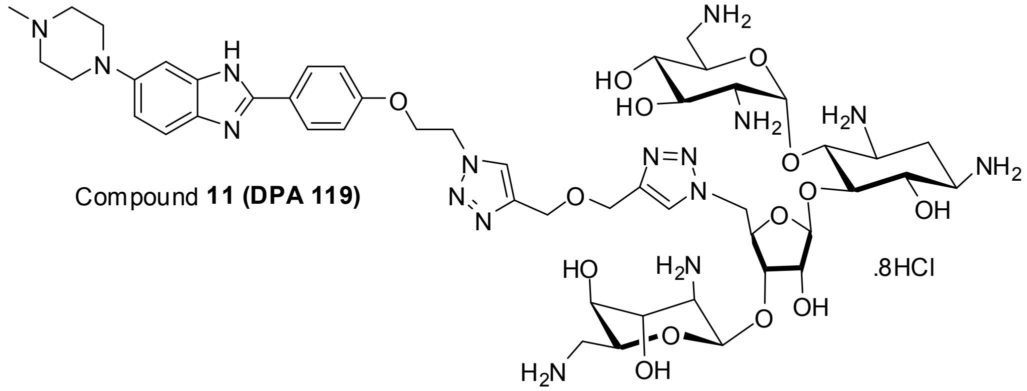

Compound **11 (DPA 119)**

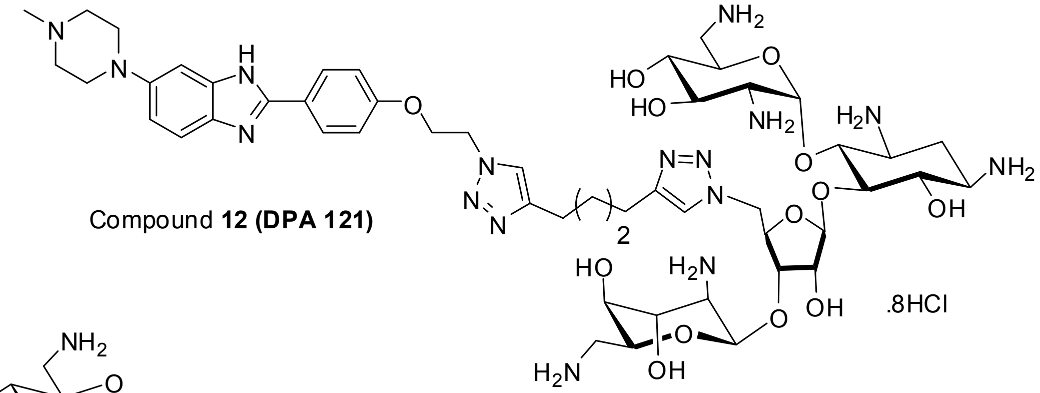

Compound **12 (DPA 121)**

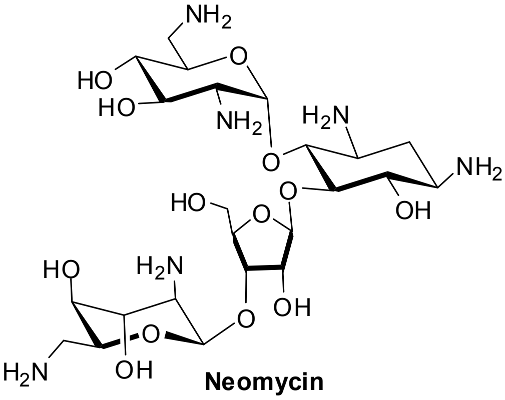

Neomycin

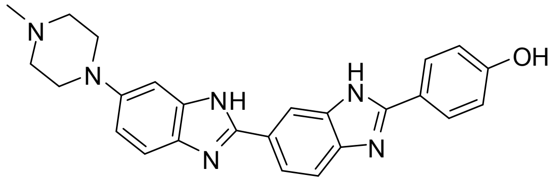

Hoechst 33258

Fig **S2**: Structures of neomycin-monobenzimidazole conjugates **8-12**, neomycin and Hoechst 33258.

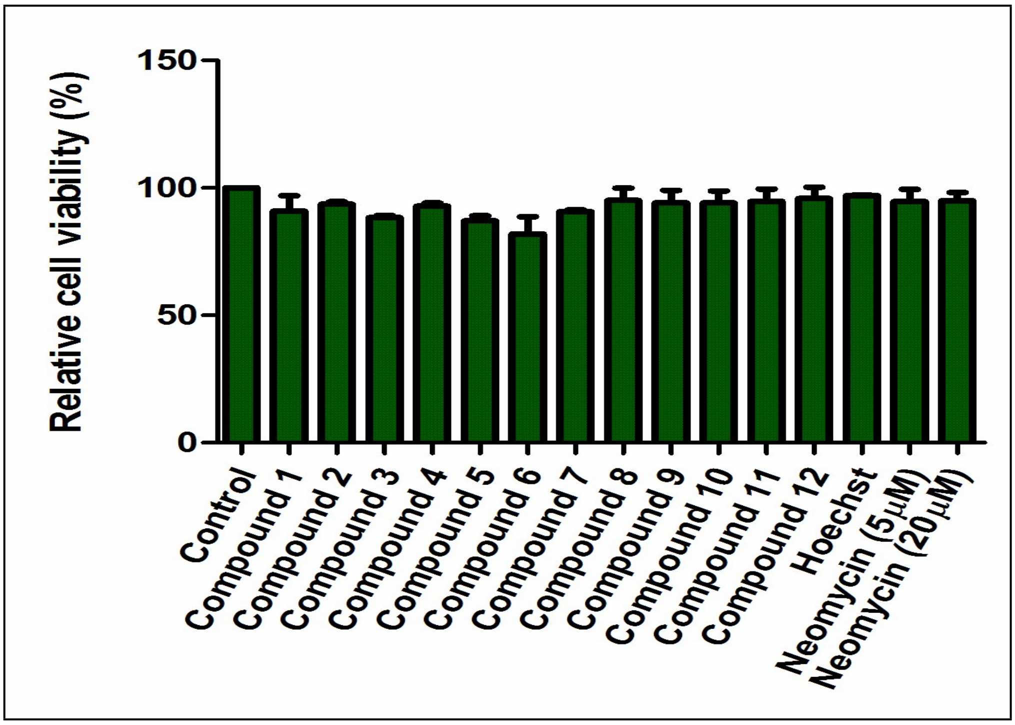

Fig S3: Effect of neomycin-mono/bisbenzimidazole conjugates on cell viability in MCF-7 cells as measured by MTT assay. 24 h exposure to the examined concentration (5 μM) of NH conjugates demonstrated no cytotoxic effect as compared to untreated cells. Parent compounds, Hoechst and Neomycin at same concentration also did not reveal any reduction in cell viability. Data are expressed as mean ± SD of three independent experiments.

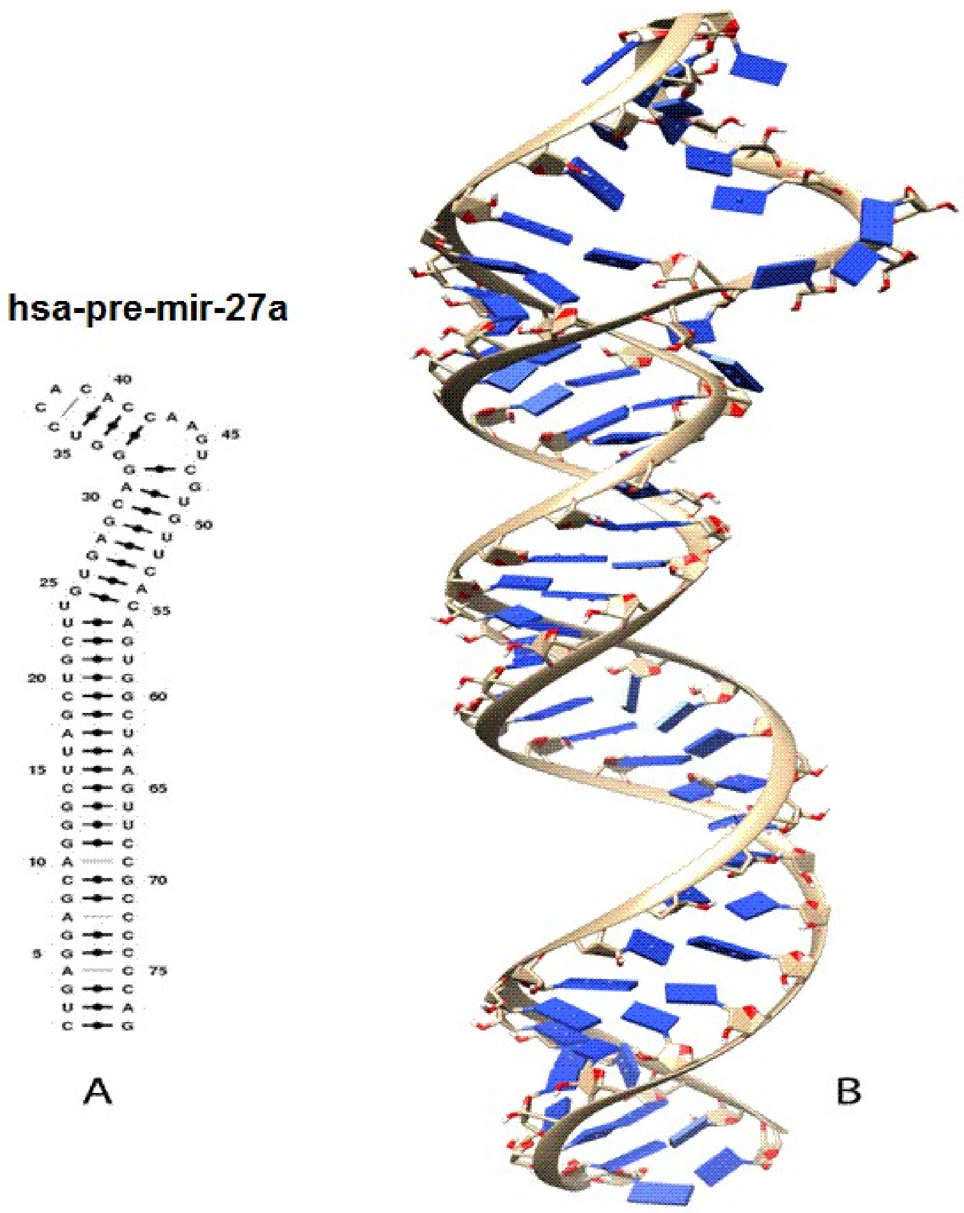

Fig S4a. Modeling of hsa-pre-mir-27a using mc-fold | mc-sym pipeline. (A) The two dimensional prediction of the microRNA. (B) The corresponding energy minimized three-dimensional predicted structure. The image was generated using Chimera.

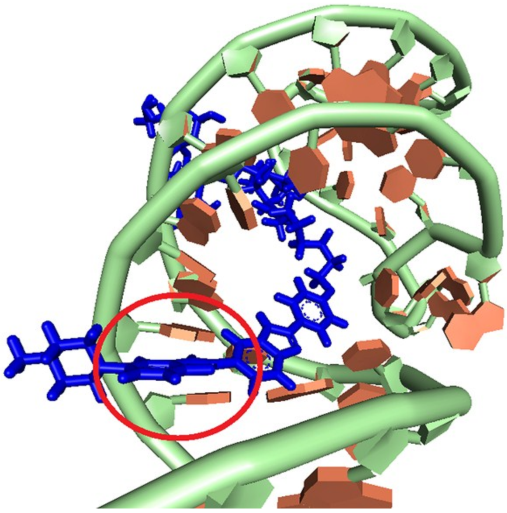

Figure S4b. A view showing the bisbenzimidazole moiety of compound **5** making stacking interactions with the bases between nucleotides 54 and 55 (circled red) of hsa- pre- mir-27a .

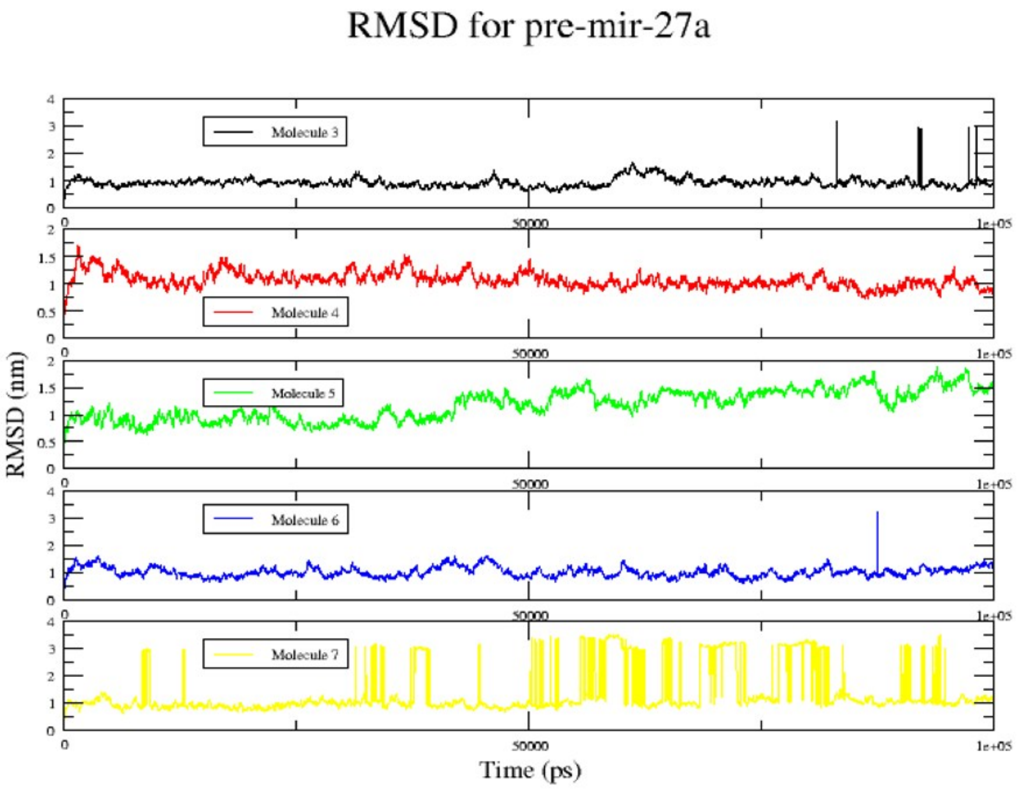

Fig S5. RMSD plots of pre-mir-27a from the MD simulation for the best-docked poses of molecules with pre- mir-27a.

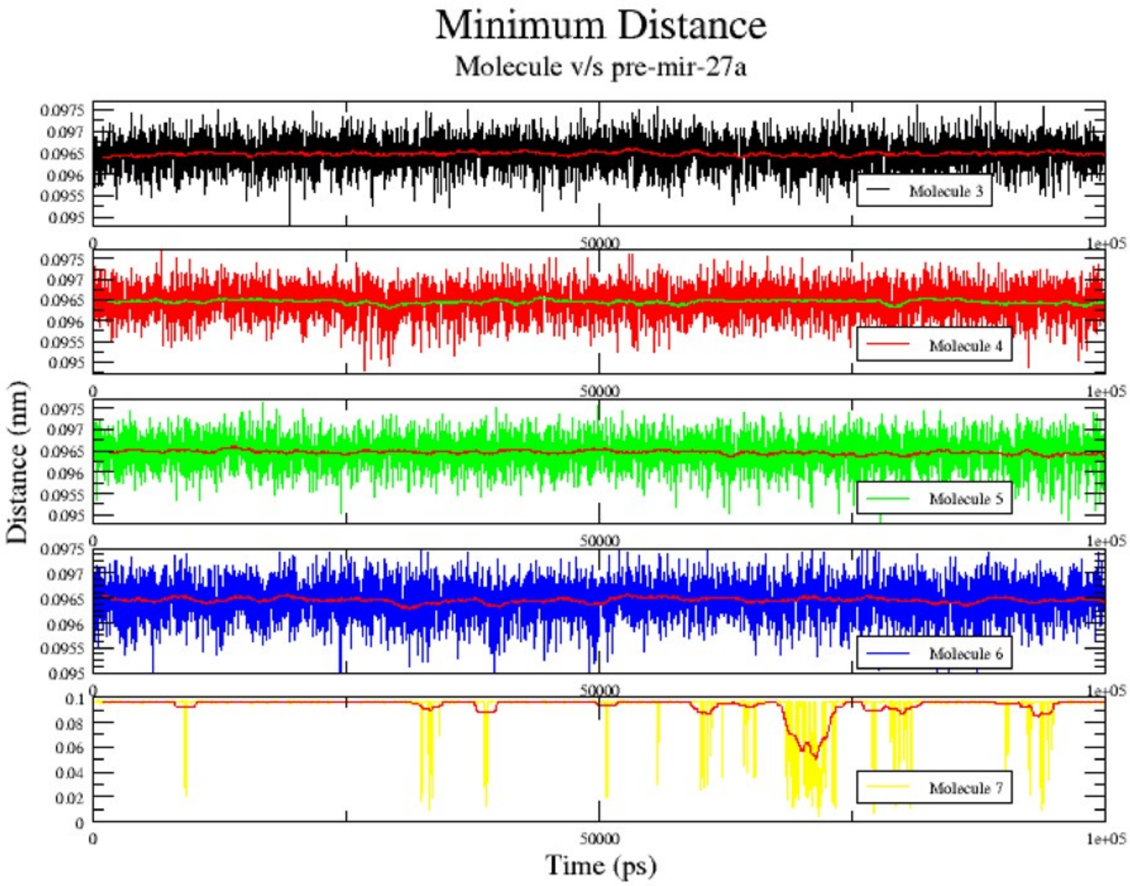

Fig S6. Distance plot between the centre of masses of the docked molecules and pre-mir-27a.

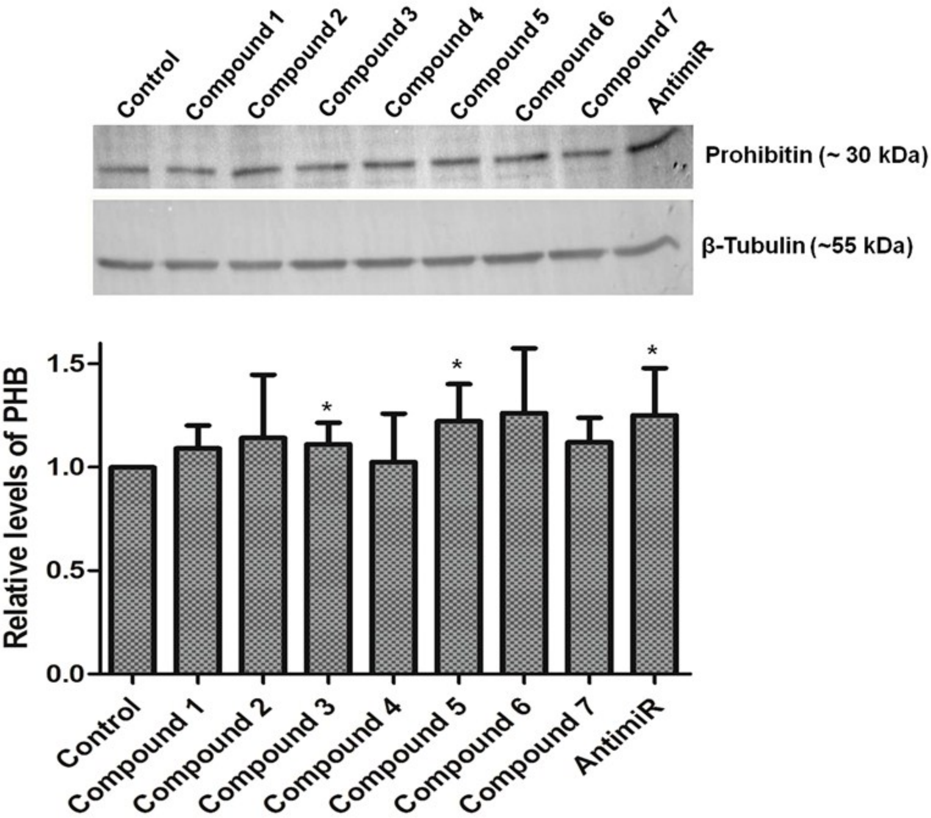

Fig S7: Western blot detection of Prohibitin levels upon treatment with NH compounds 1-7 at a final concentration of 5 μM. Compound 3, 5 and 6 showed increase in PHB levels whereas; compound 1, 2 and 4 did not procure any change. AntimiR-27a (100 nM) was used as a positive control. β-Tubulin was used as an endogenous internal control.

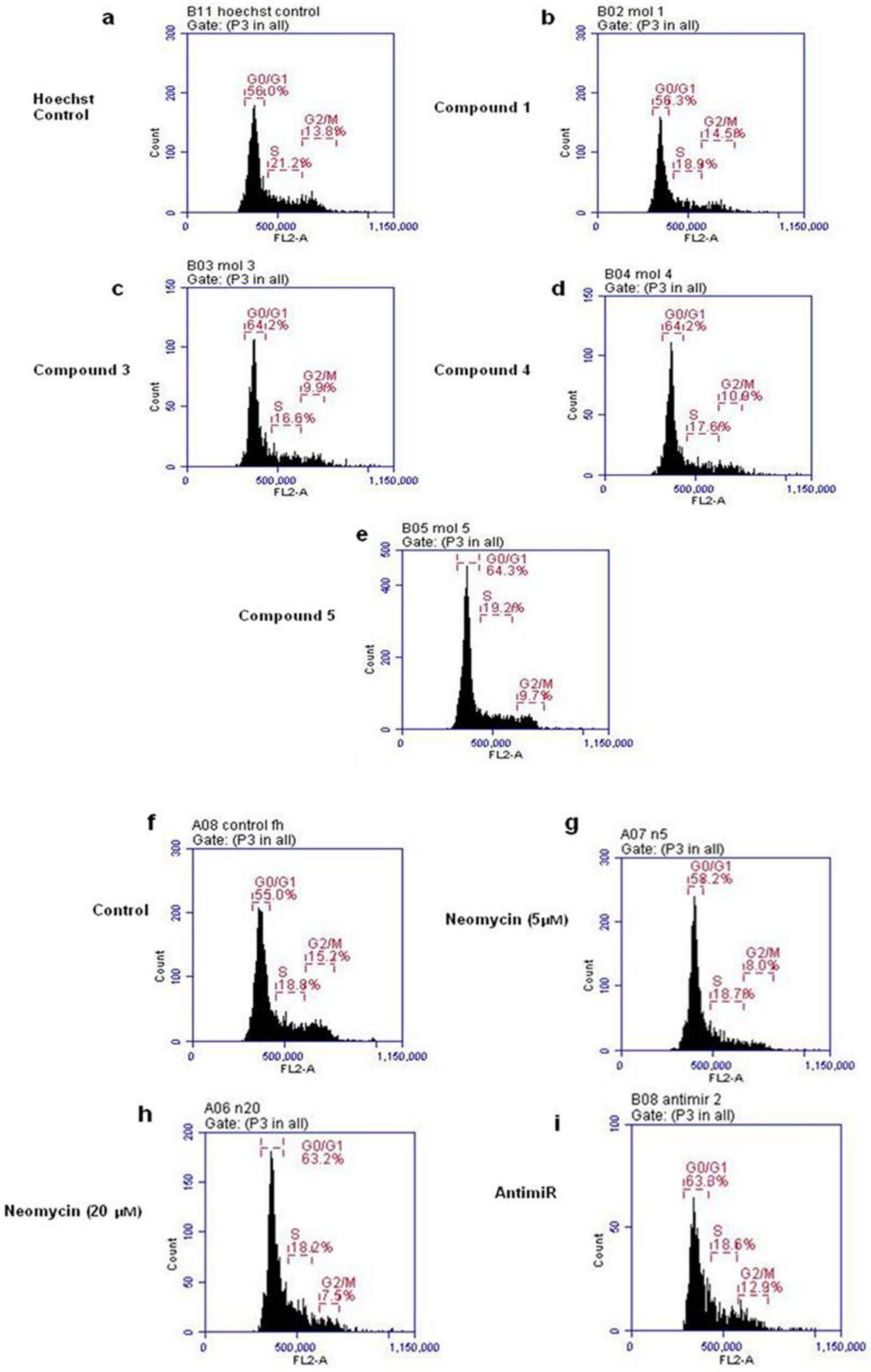

a
B11 hoechst control
Gate: (P3 in all)
Hoechst Control
G0/G1 56.0%
S 21.2%
G2/M 13.8%
b
B02 mol 1
Gate: (P3 in all)
Compound 1
G0/G1 56.3%
S 18.9%
G2/M 14.5%
c
B03 mol 3
Gate: (P3 in all)
Compound 3
G0/G1 64.2%
S 16.6%
G2/M 9.9%
d
B04 mol 4
Gate: (P3 in all)
Compound 4
G0/G1 64.2%
S 17.6%
G2/M 10.9%
e
B05 mol 5
Gate: (P3 in all)
Compound 5
G0/G1 64.3%
S 19.2%
G2/M 9.7%
f
A08 control fh
Gate: (P3 in all)
Control
G0/G1 55.0%
S 18.8%
G2/M 15.2%
g
A07 n5
Gate: (P3 in all)
Neomycin (5μM)
G0/G1 58.2%
S 18.7%
G2/M 8.0%
h
A06 n20
Gate: (P3 in all)
Neomycin (20 μM)
G0/G1 63.2%
S 18.2%
G2/M 7.5%
i
B08 antimir 2
Gate: (P3 in all)
AntimiR
G0/G1 63.8%
S 18.6%
G2/M 12.9%
Count
FL2-A
0
500,000
1,150,000

Fig S8: Representative histogram plots of FACS mediated cell cycle analysis upon treatment with compounds **1, 3 4** and **5** at a final concentration of 5 µM. Compound **3, 4** and **5** showed increase in G0/G1 levels and decrease in S phase levels whereas; compound 1 did not have significant change in G0/G1 phase as compared to the Hoechst 33258 control (a-e). The G2/M phase levels did not procure any significant changes after treatment with any compounds. The cells treated with Neomycin at 5 µM (g) 20 µM (h) and antimiR (i) were compared to untreated cells stained with Pi (f).

^{1}H NMR, ^{13}C NMR, IR, and MALDI-TOF spectra of all new compounds for the synthesis of conjugates 1-12.

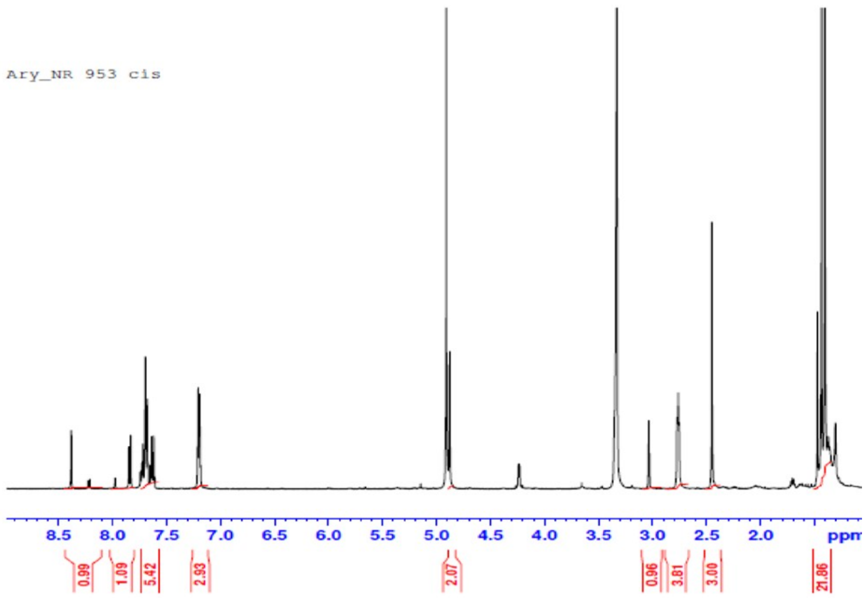

^{1}H NMR spectrum of **1b** (rotamer I)

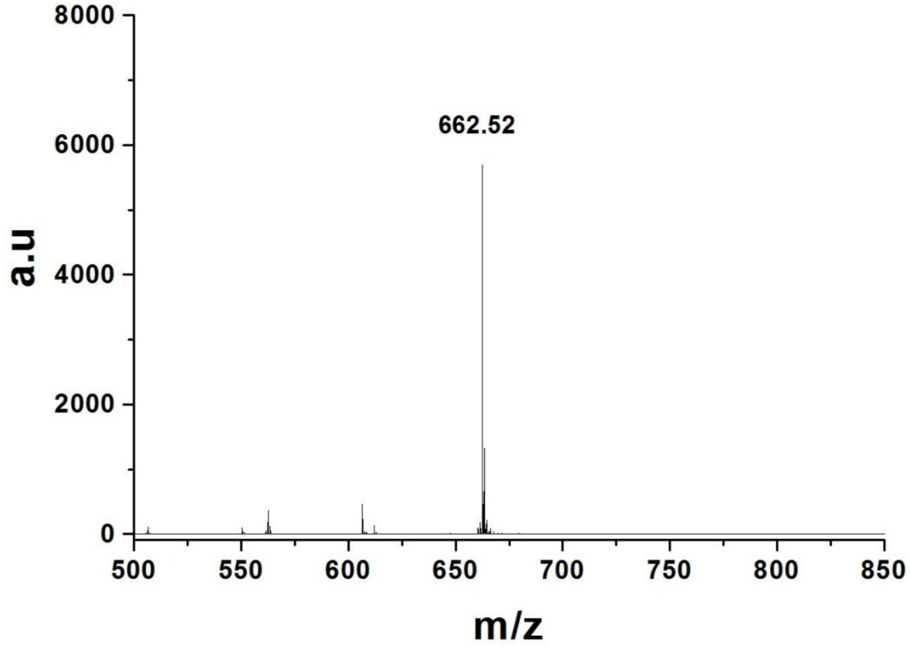

MALDI-TOF spectrum of **1b** (rotamer I)

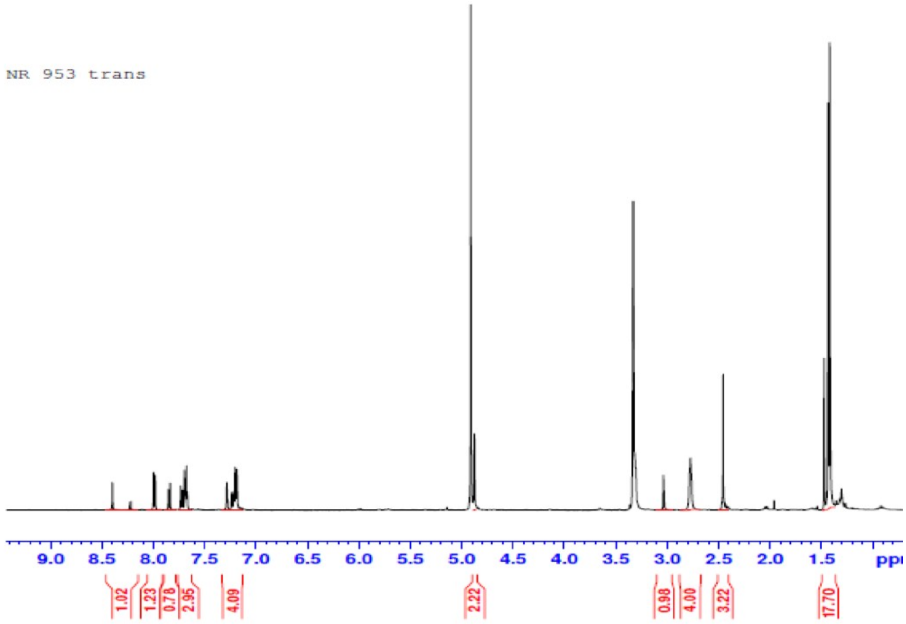

^{1}H NMR spectrum of **1b** (rotamer II)

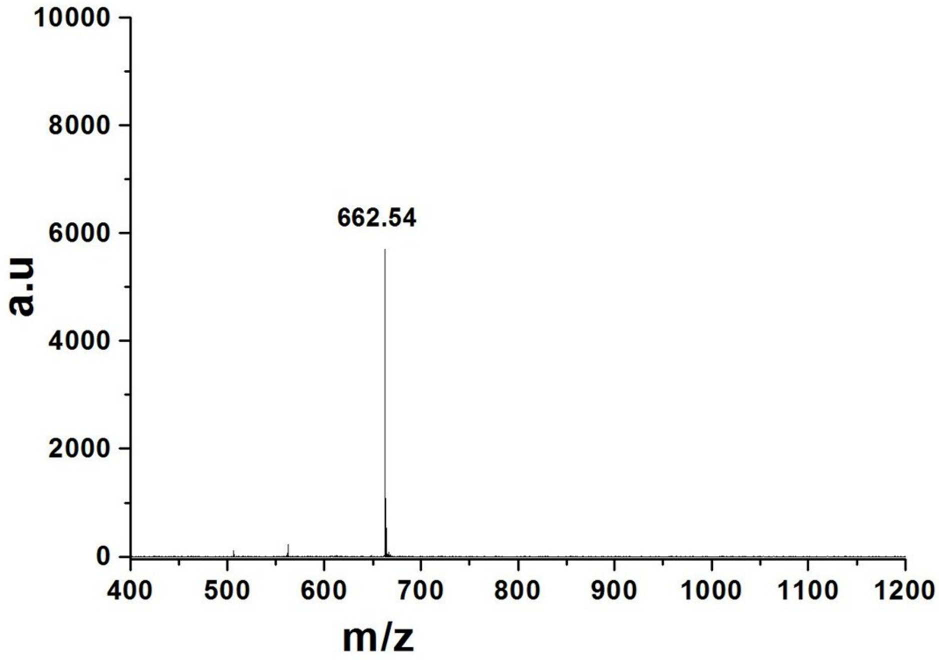

MALDI-TOF spectrum of **1b** (rotamer II)

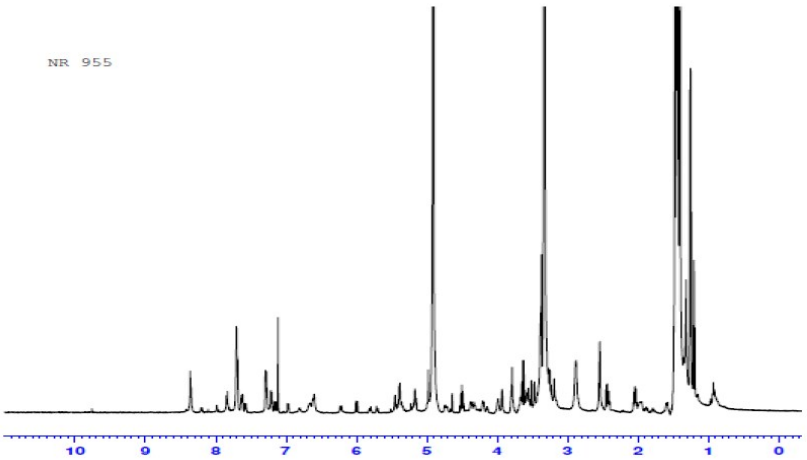

^{1}H NMR spectrum of Boc protected **1**.

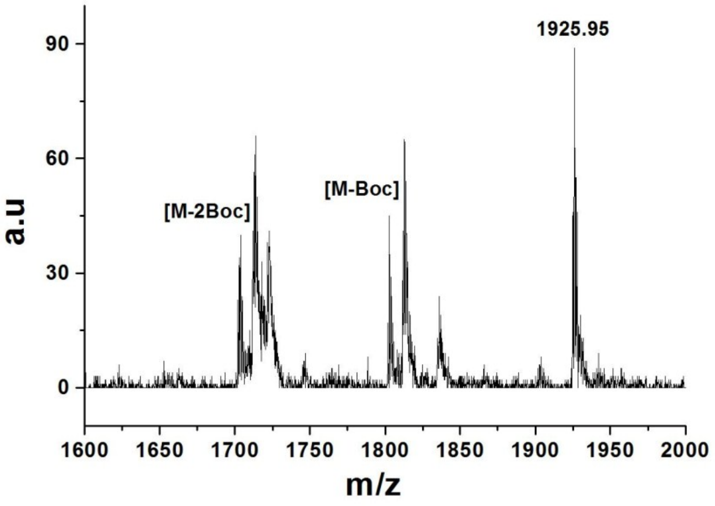

MALDI-TOF spectrum of Boc protected **1**.

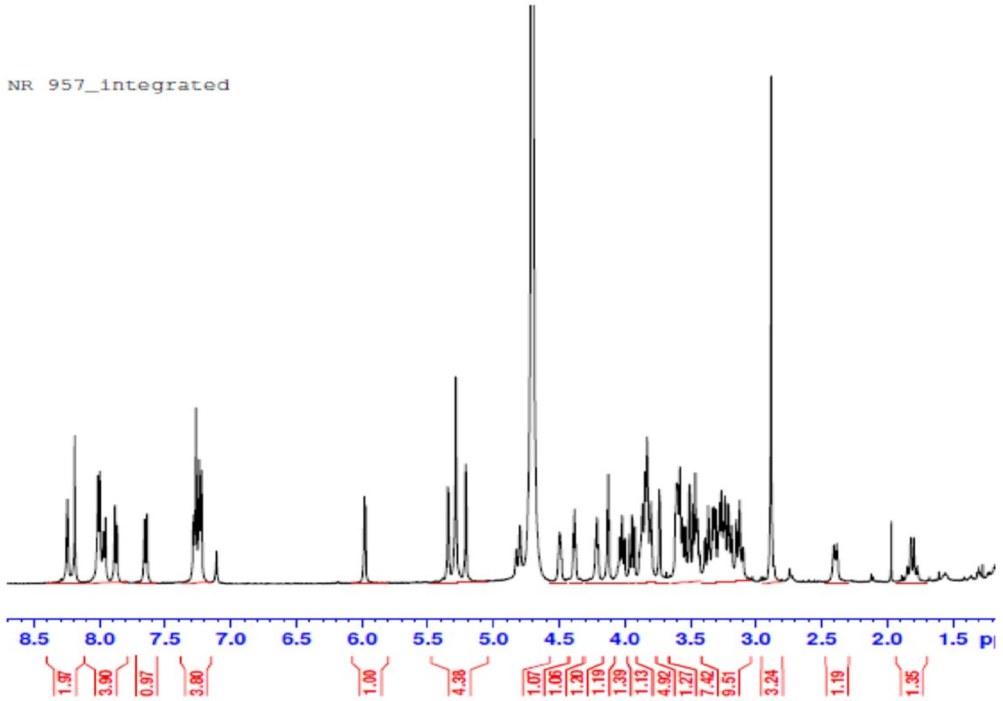

^{1}H NMR spectrum of **1**.

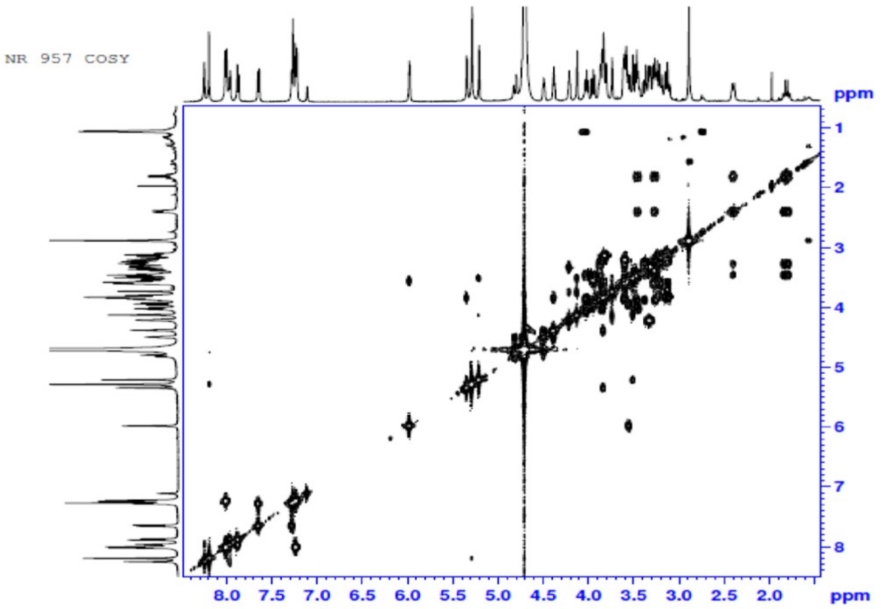

^{1}H-^{1}H COSY spectrum of **1**.

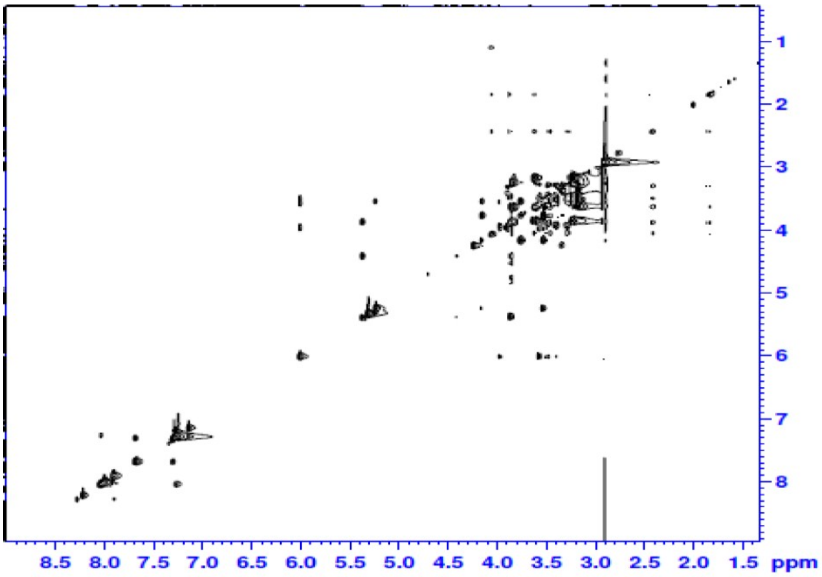

TOCSY spectrum of **1**.

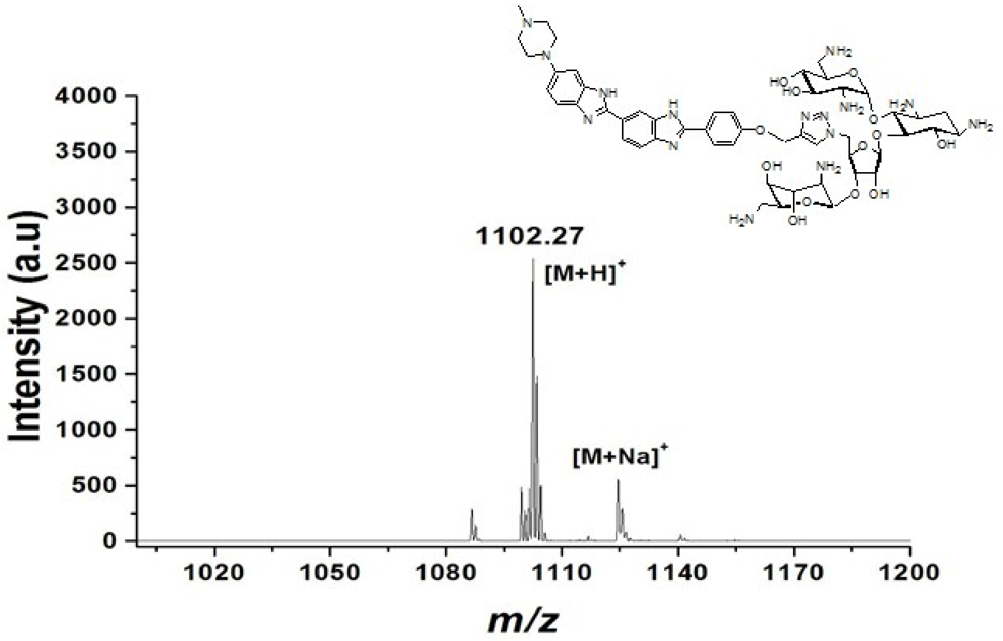

MALDI-TOF spectrum of **1**.

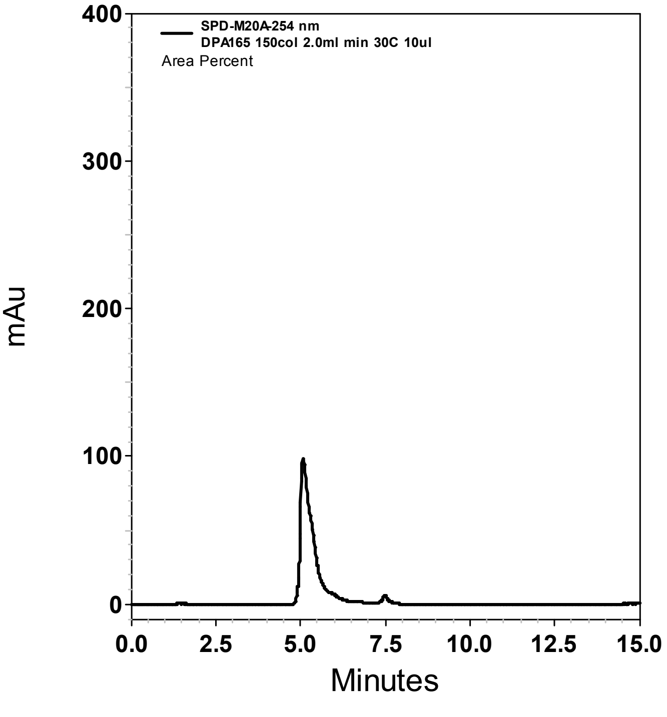

HPLC spectrum of **1**.

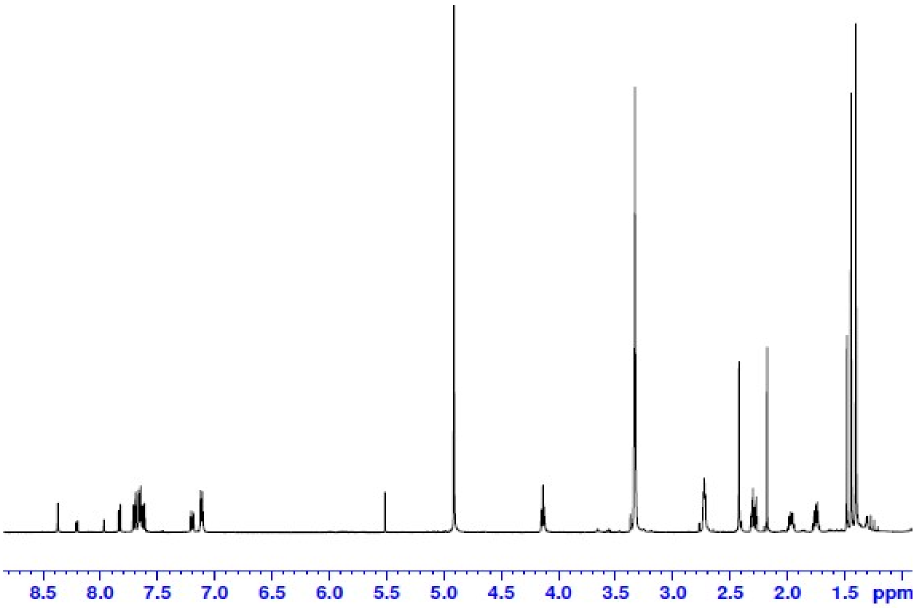

^{1}H NMR spectrum of **2b** (rotamer I)

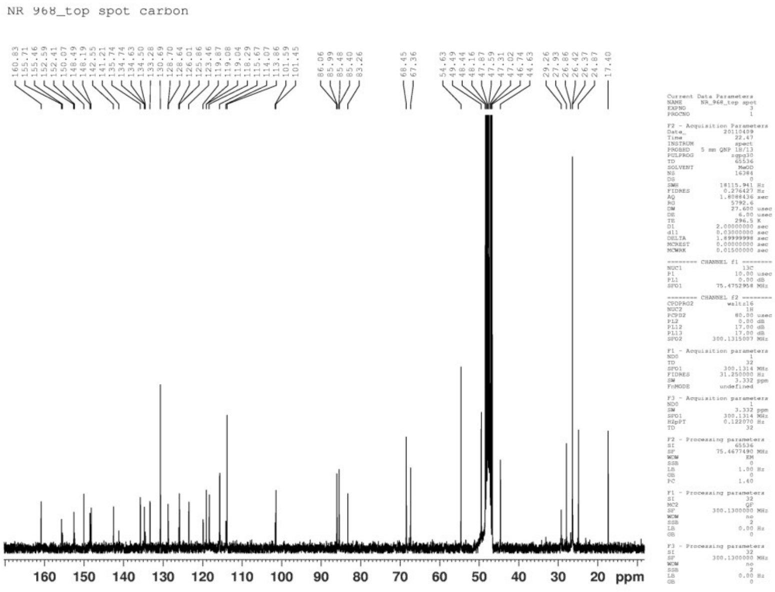

^{13}C NMR spectrum of **2b** (rotamer I).

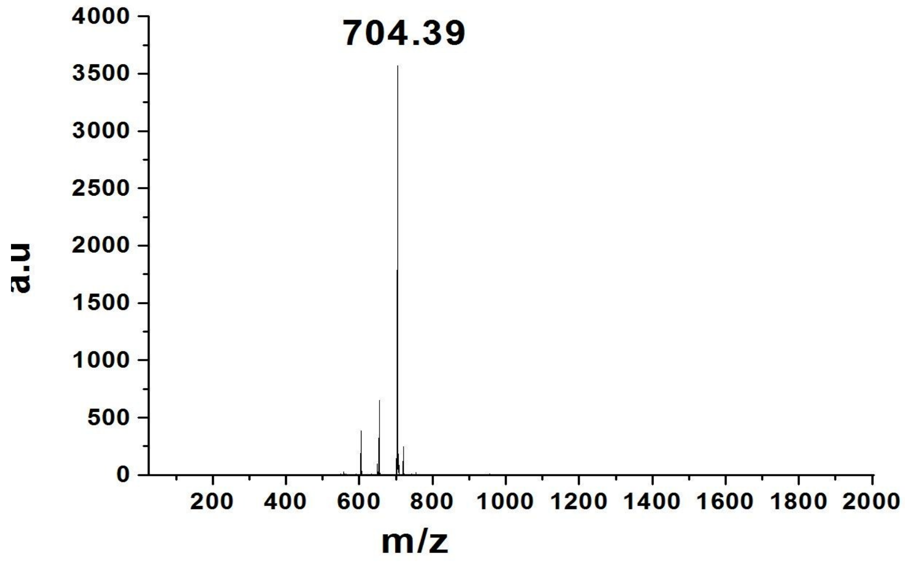

MALDI-TOF spectrum of **2b** (rotamer I)

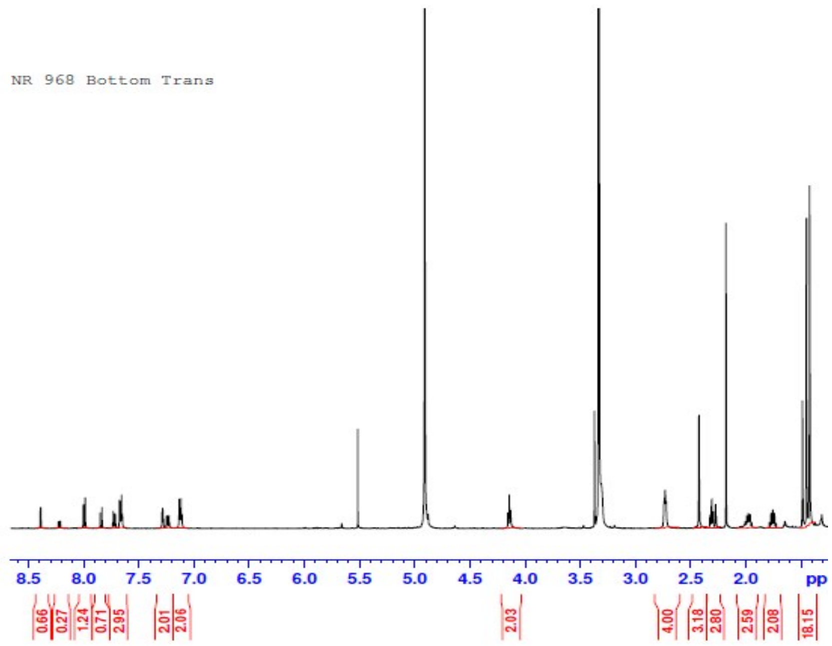

^{1}H NMR spectrum of **2b** (rotamer II)

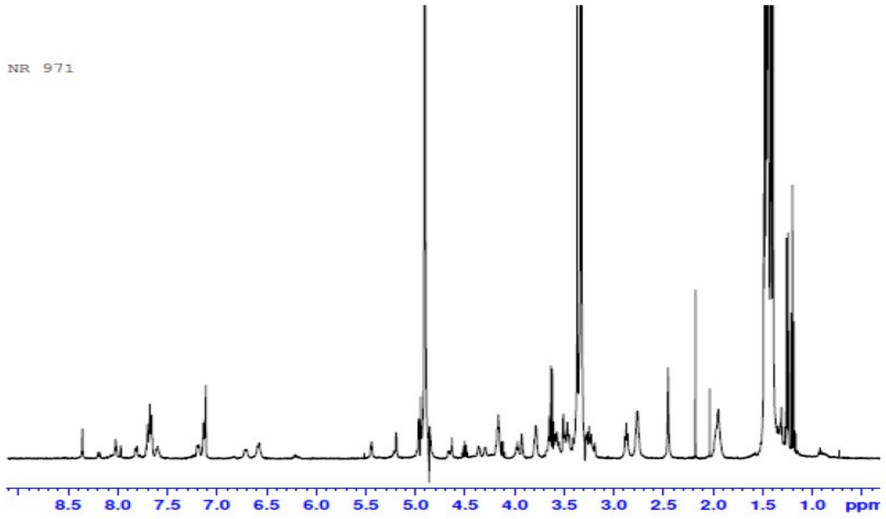

^1H NMR spectrum of Boc protected **2**.

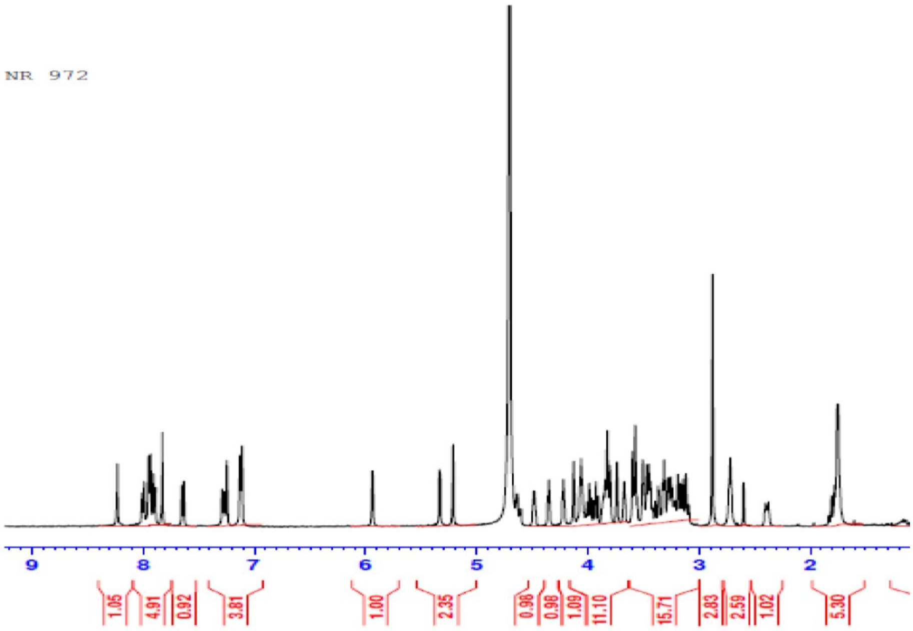

^1H NMR spectrum of **2**.

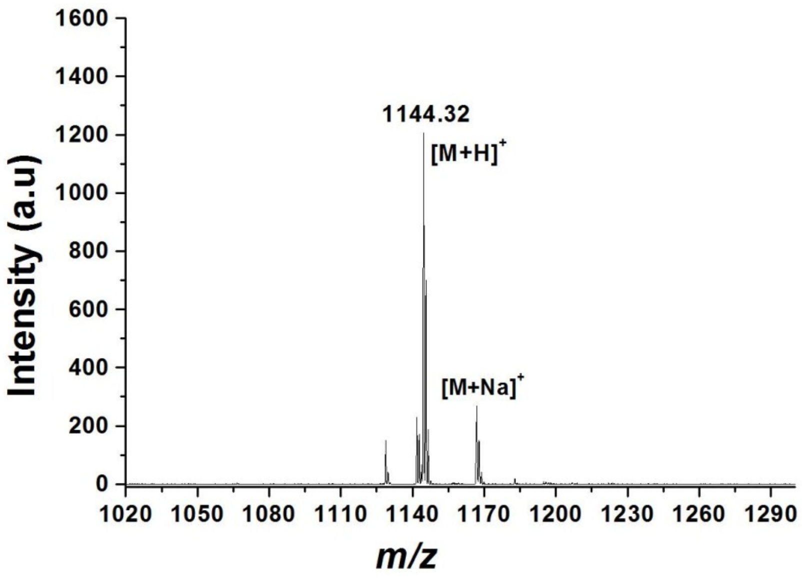

MALDI-TOF spectrum of **2**.

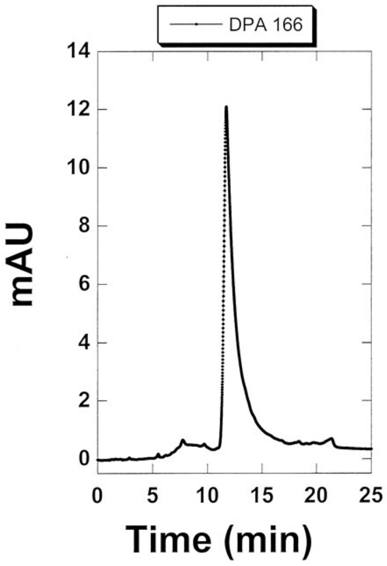

HPLC spectrum of **2**.

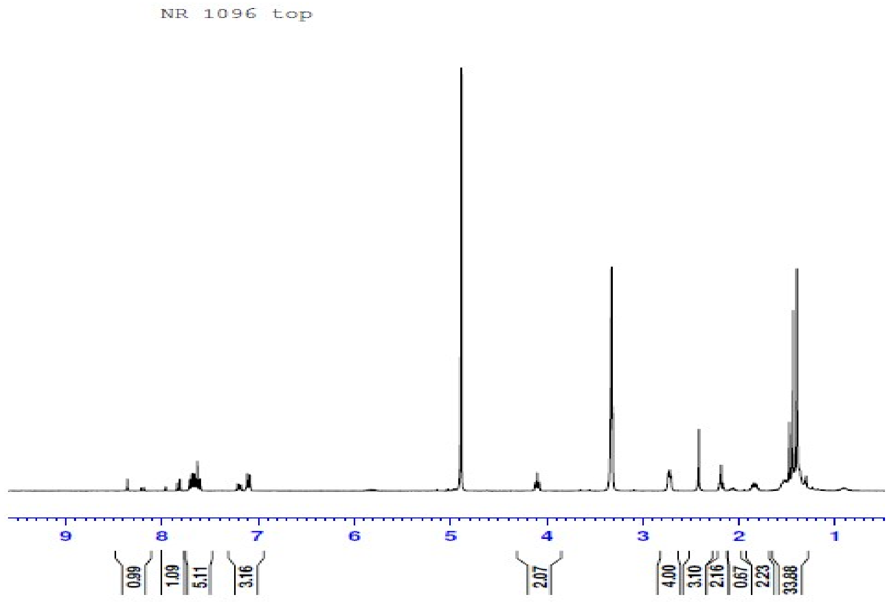

^{1}H NMR spectrum of **3b** (rotamer I).

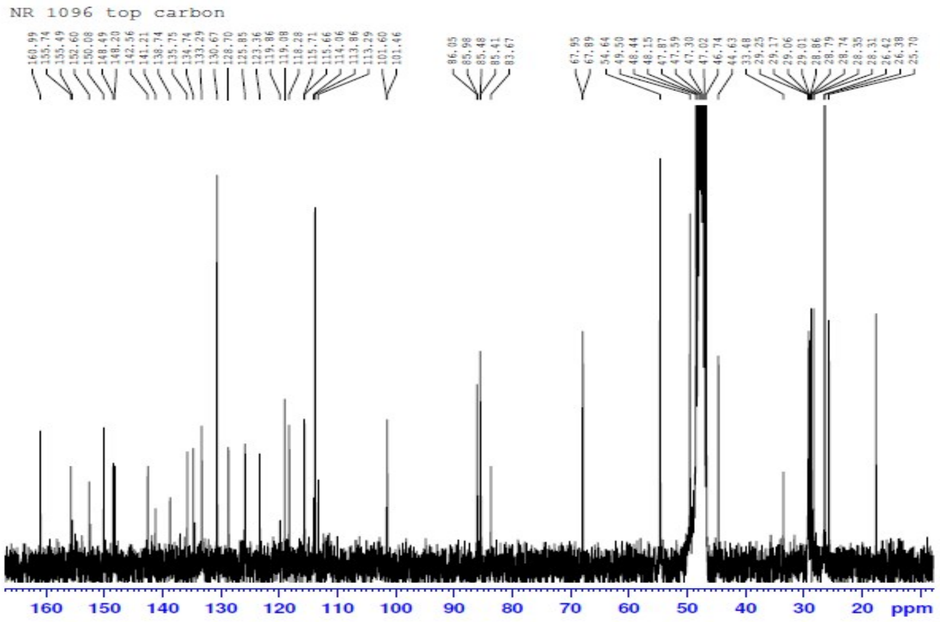

^{13}C NMR of DPA **3b** (rotamer I).

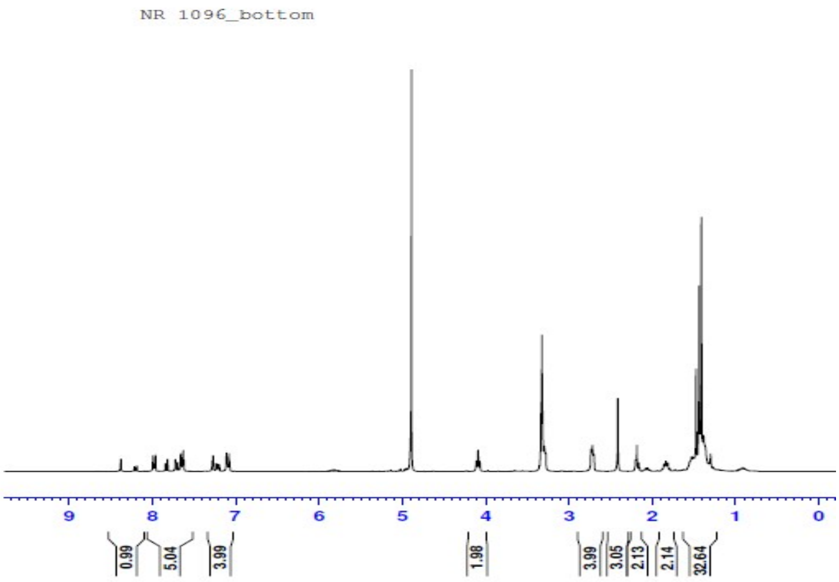

^{1}H NMR spectrum of **3b** (rotamer II).

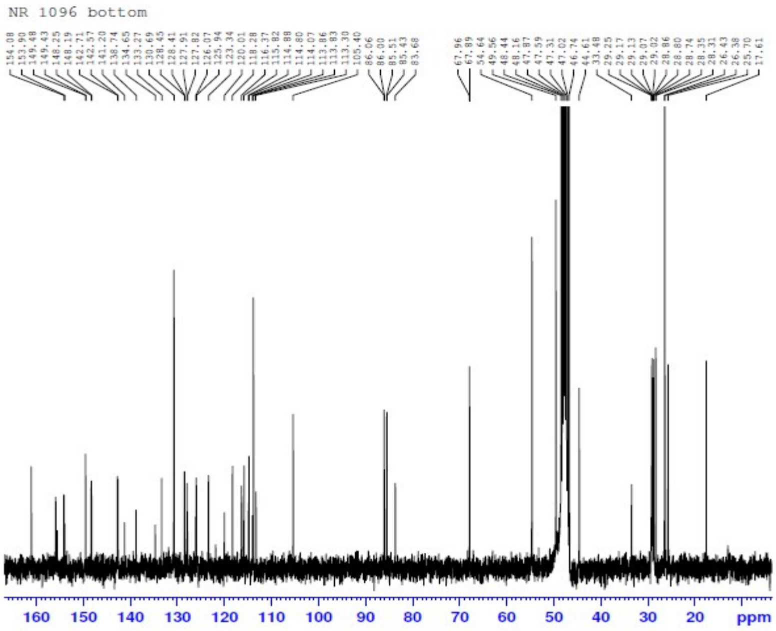

^{13}C NMR spectrum of **3b** (rotamer II).

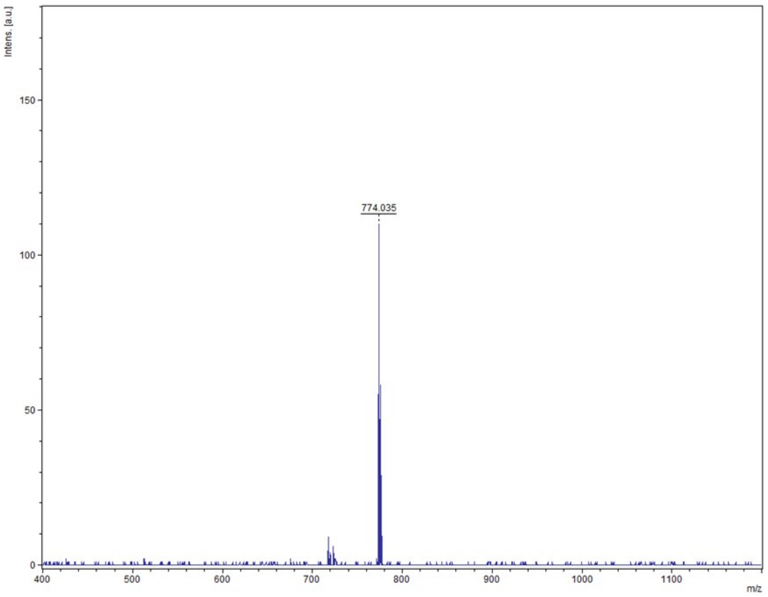

MALDI-TOF spectrum of **3b** (rotamer I)

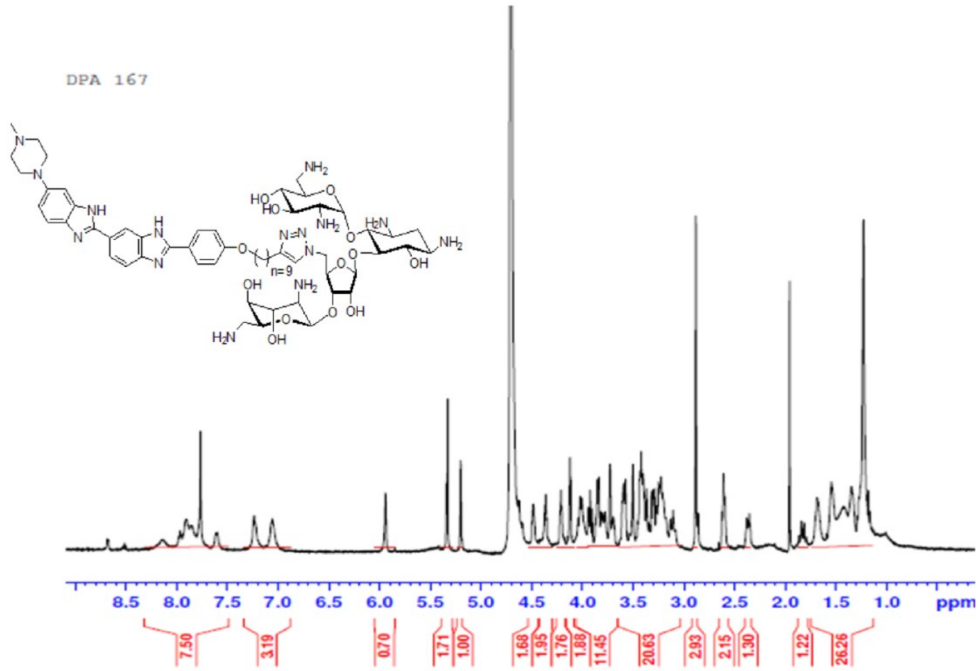

^{1}H NMR spectrum of **3**.

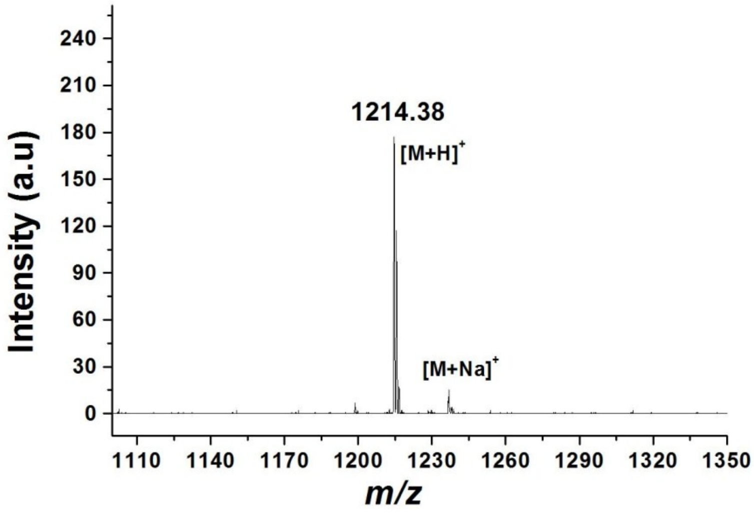

MALDI-TOF spectrum of **3**

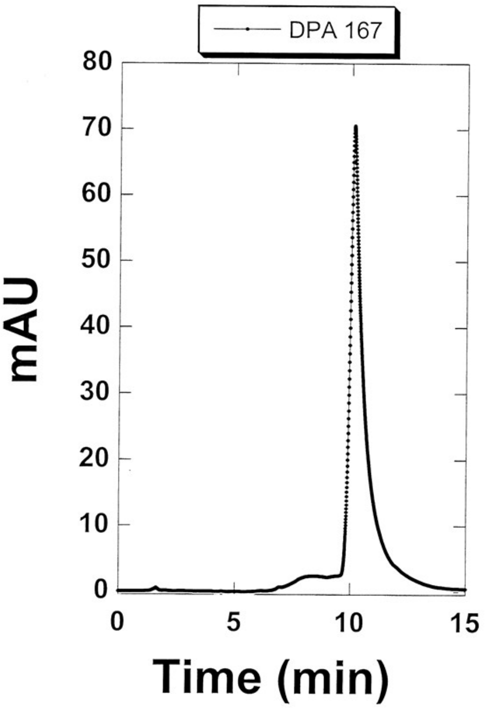

HPLC spectrum of **3**.

.

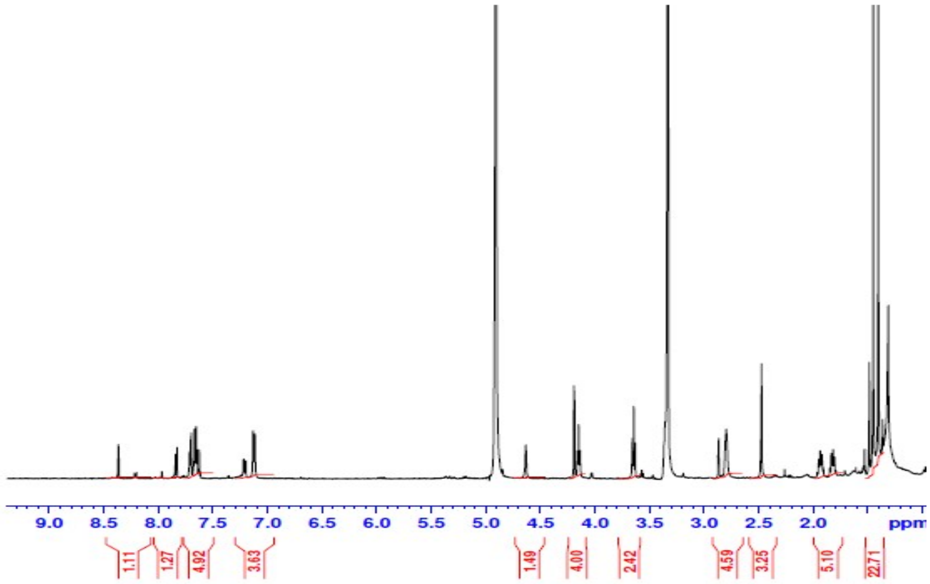

^{1}H NMR spectrum of **4b** (rotamer I).

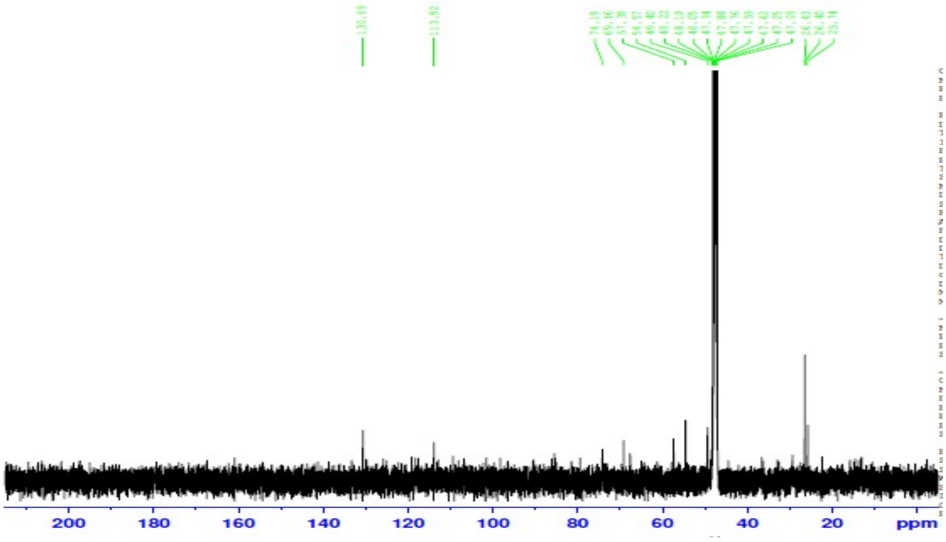

^{13}C NMR spectrum of **4b** (rotamer I).

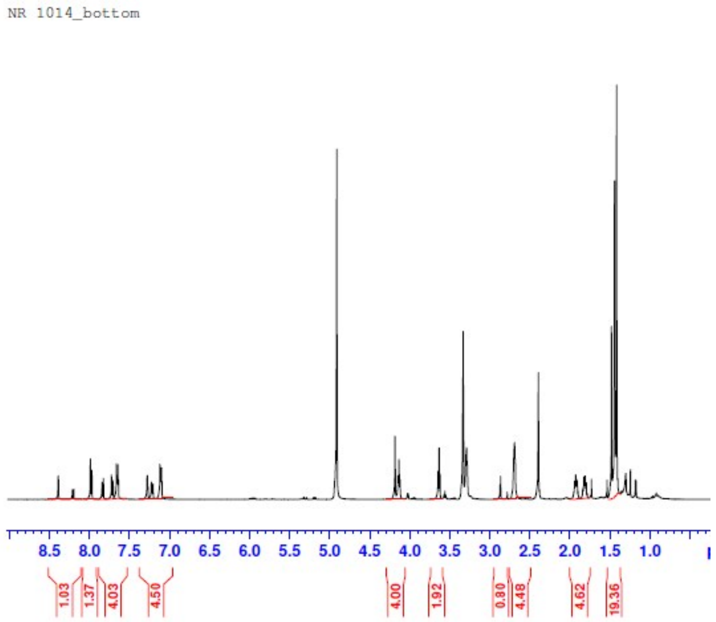

^{1}H NMR spectrum of **4b** (rotamer II).

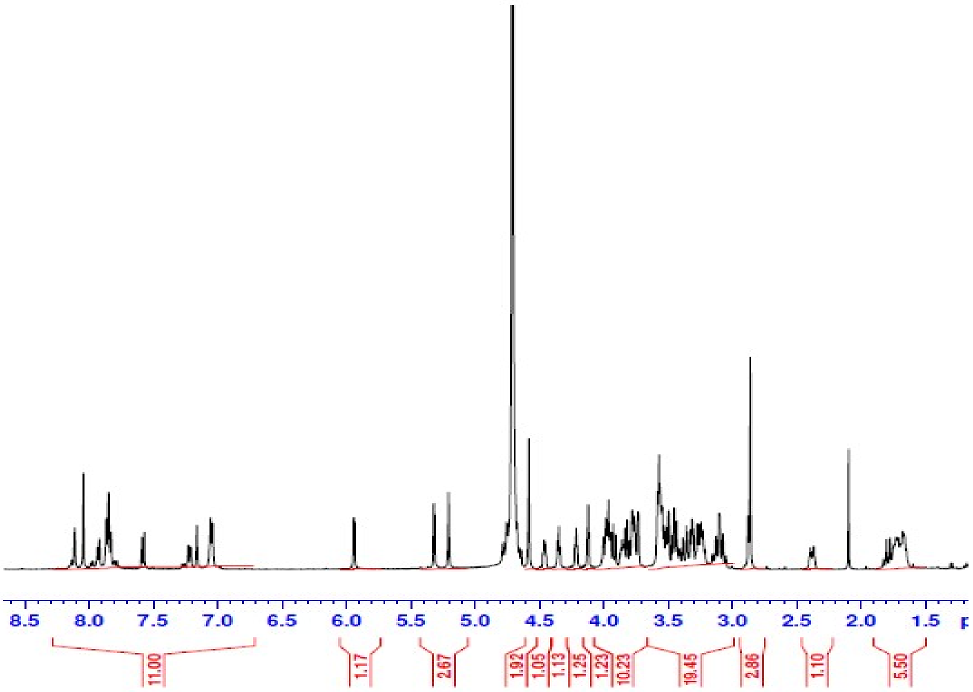

^{1}H NMR spectrum of **4**.

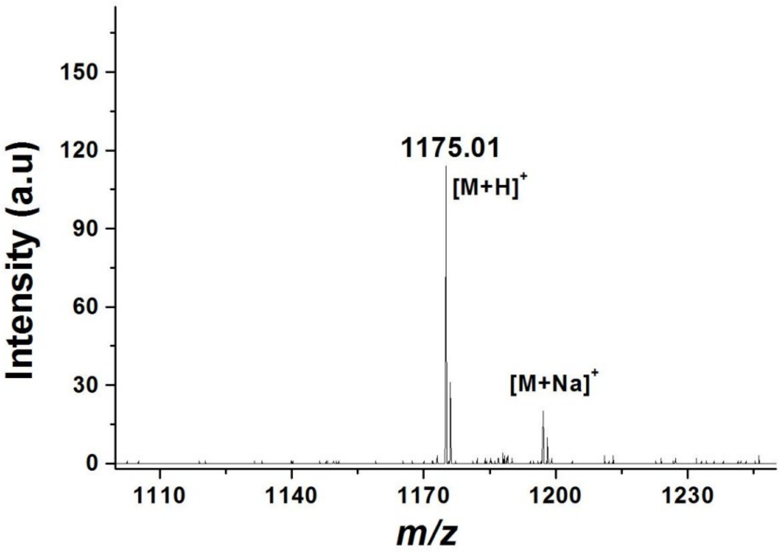

MALDI-TOF spectrum of **4**.

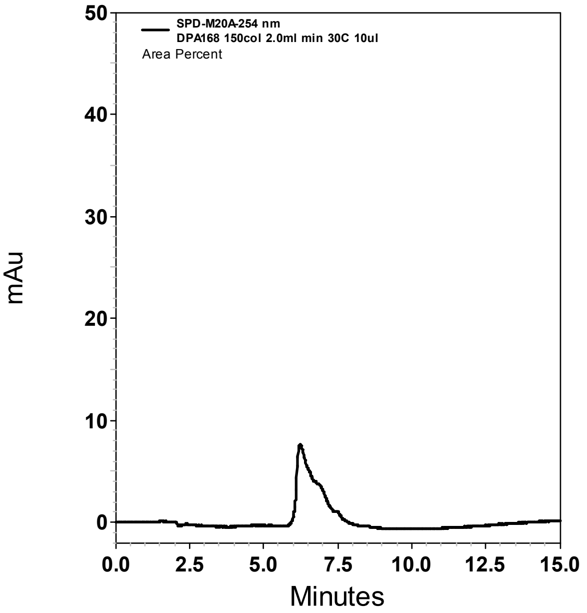

HPLC spectrum of **4**.

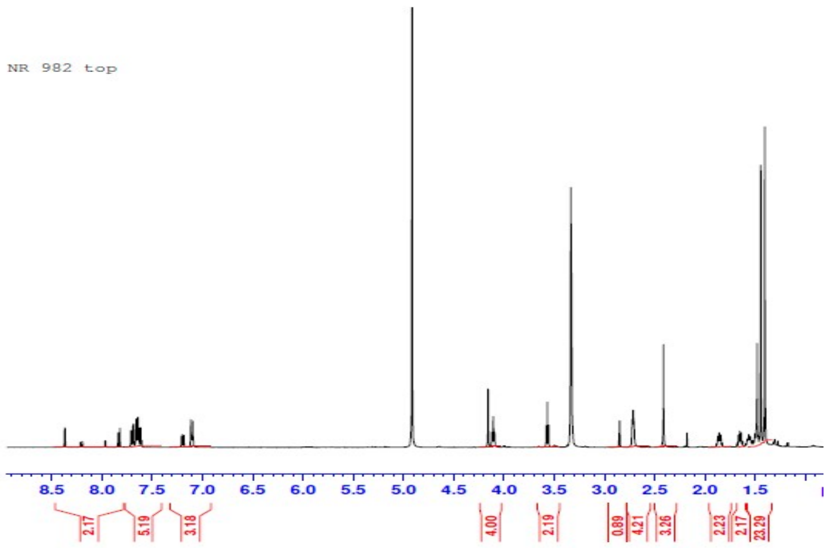

^{1}H NMR spectrum of **5b** (rotamer I).

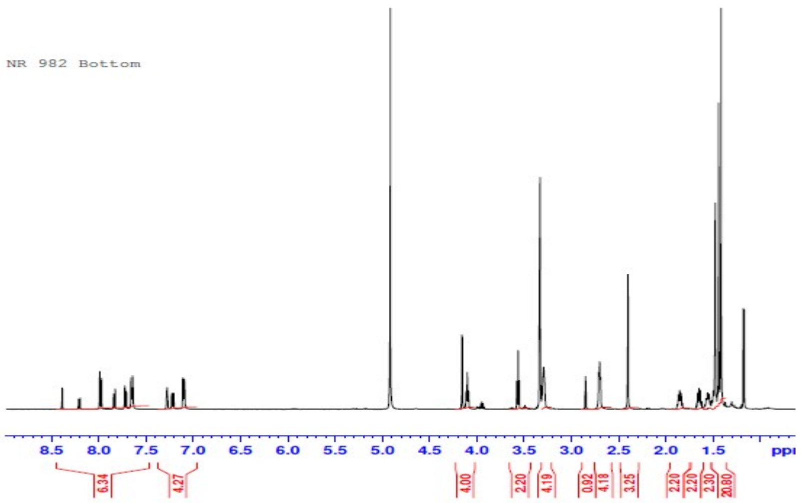

^{1}H NMR spectrum of **5b** (rotamer II).

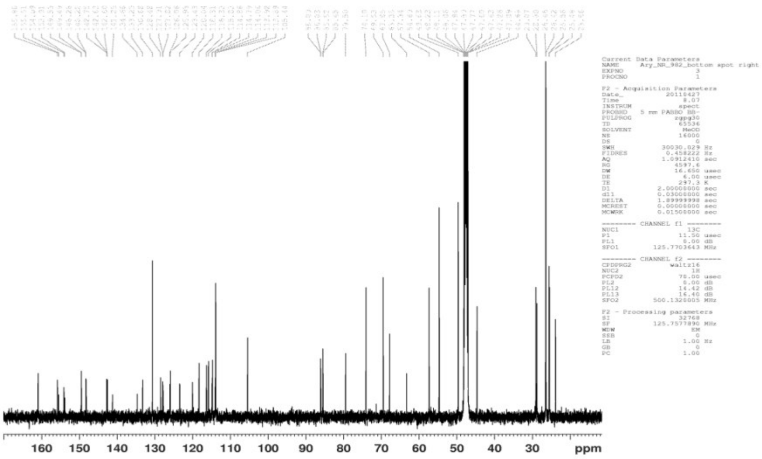

^{13}C NMR spectrum of **5b** (rotamer II).

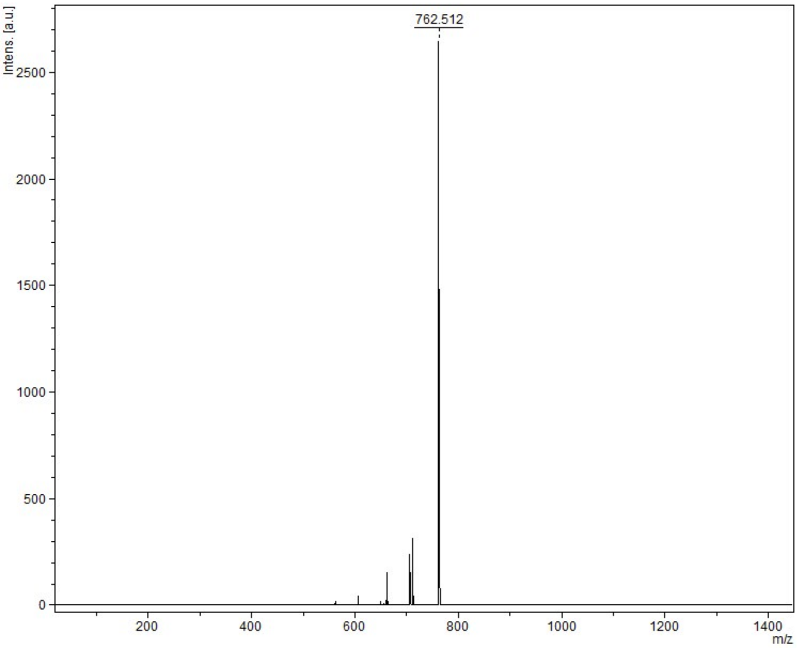

MALDI-TOF spectrum of **5b** (rotamer I).

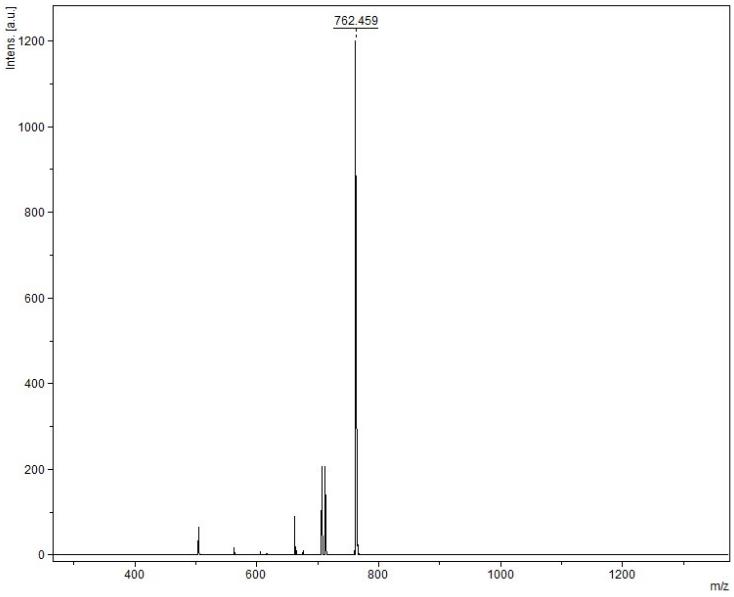

MALDI-TOF spectrum of **5b** (rotamer II).

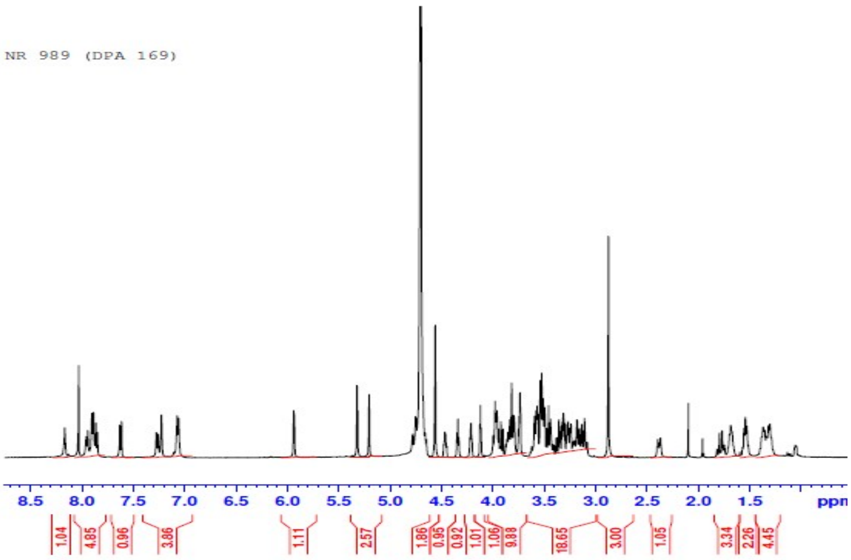

^{1}H NMR spectrum of **5**.

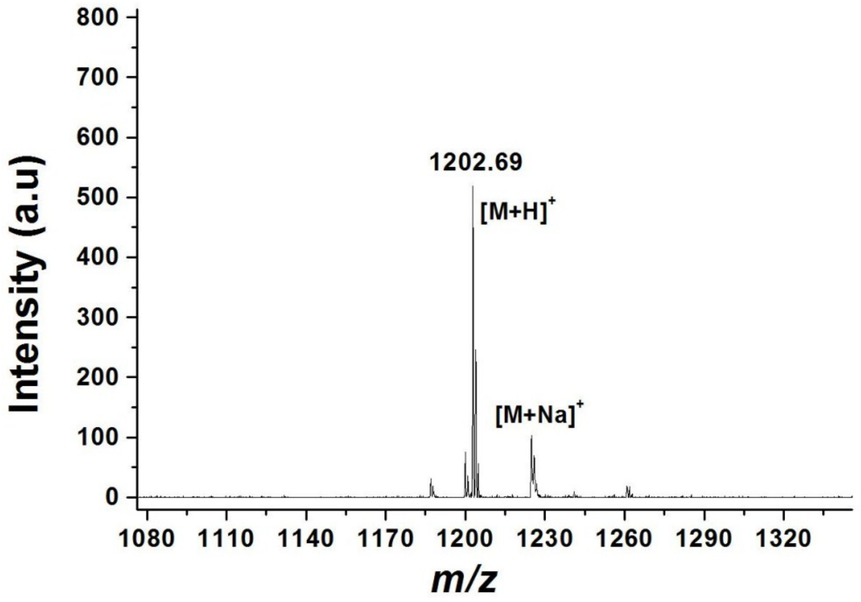

MALDI-TOF spectrum of **5**.

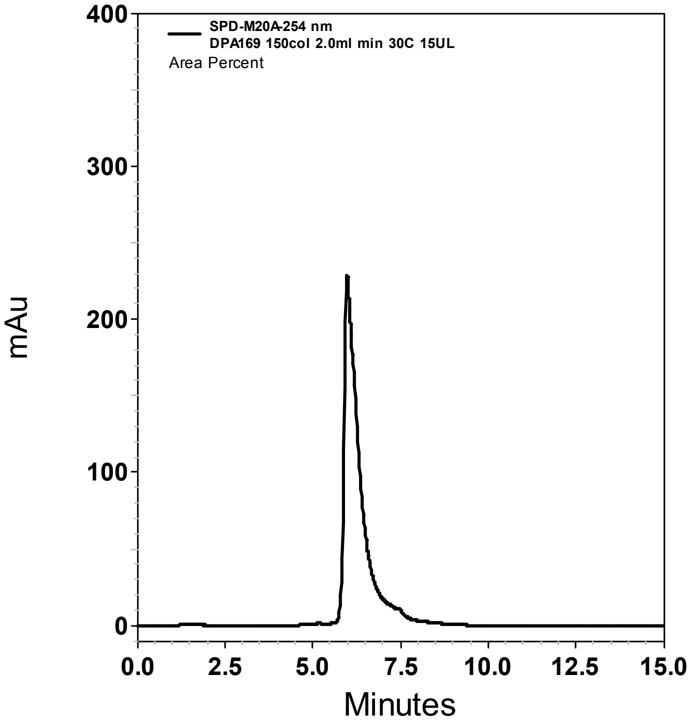

HPLC spectrum of **5**.

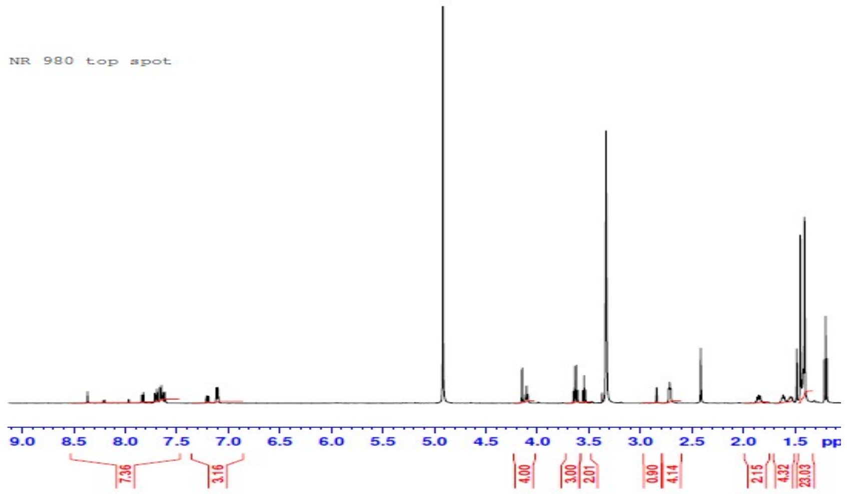

^{1}H NMR of **6b** (rotamer I).

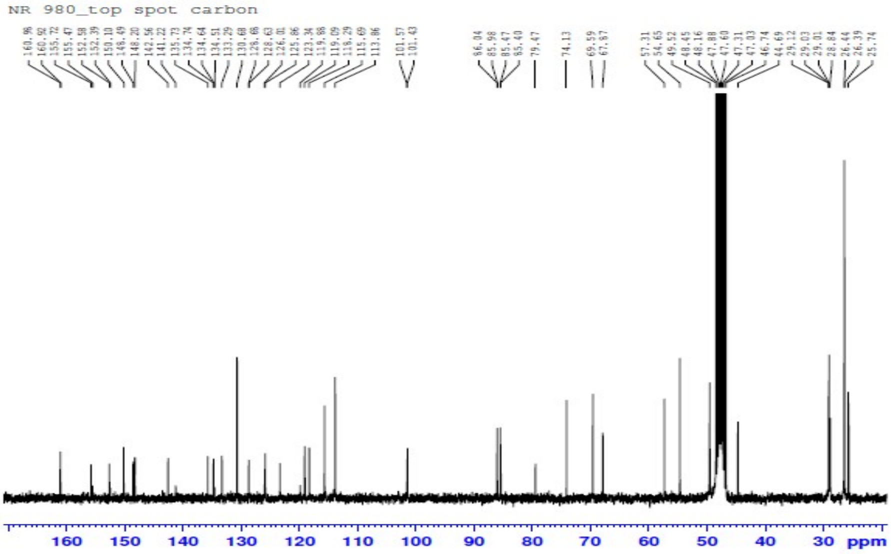

^{13}C NMR spectrum of **6b** (rotamer I).

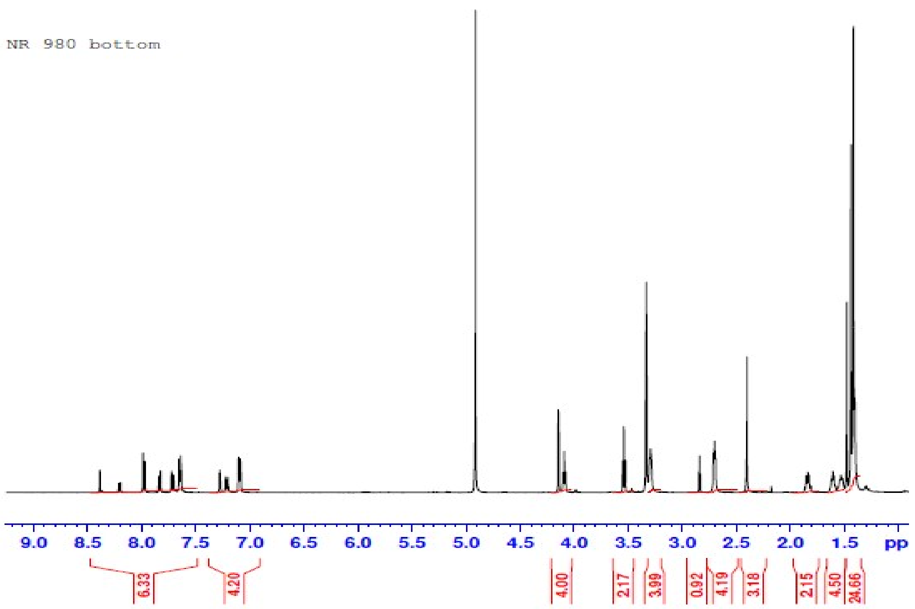

^{1}H NMR spectrum of **6b** (rotamer II).

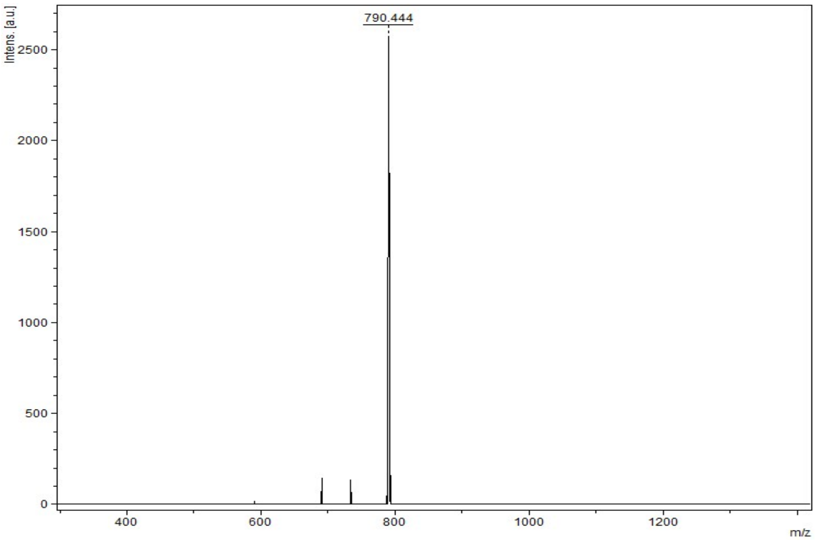

MALDI-TOF spectrum of **6b** (rotamer I).

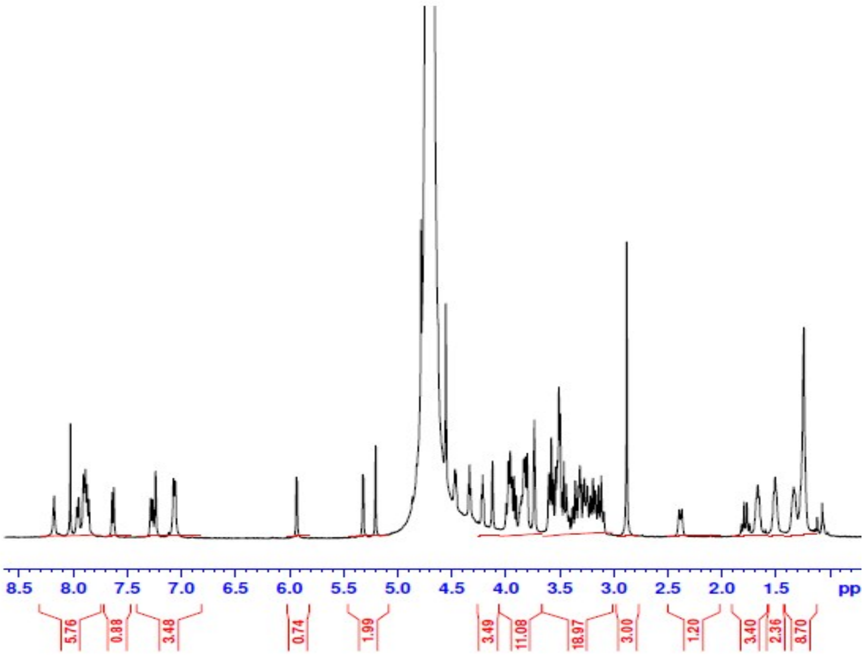

^{1}H NMR spectrum of **6**.

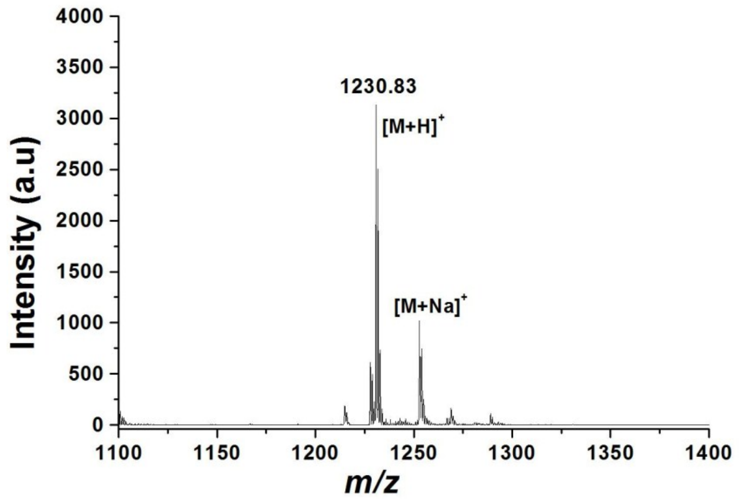

MALDI-TOF spectrum of **6**.

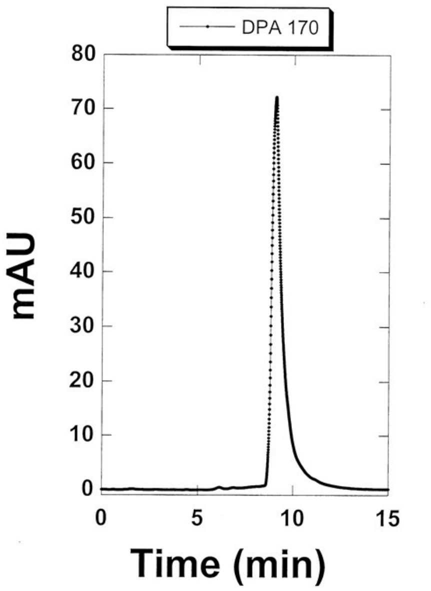

HPLC spectrum of **6**.

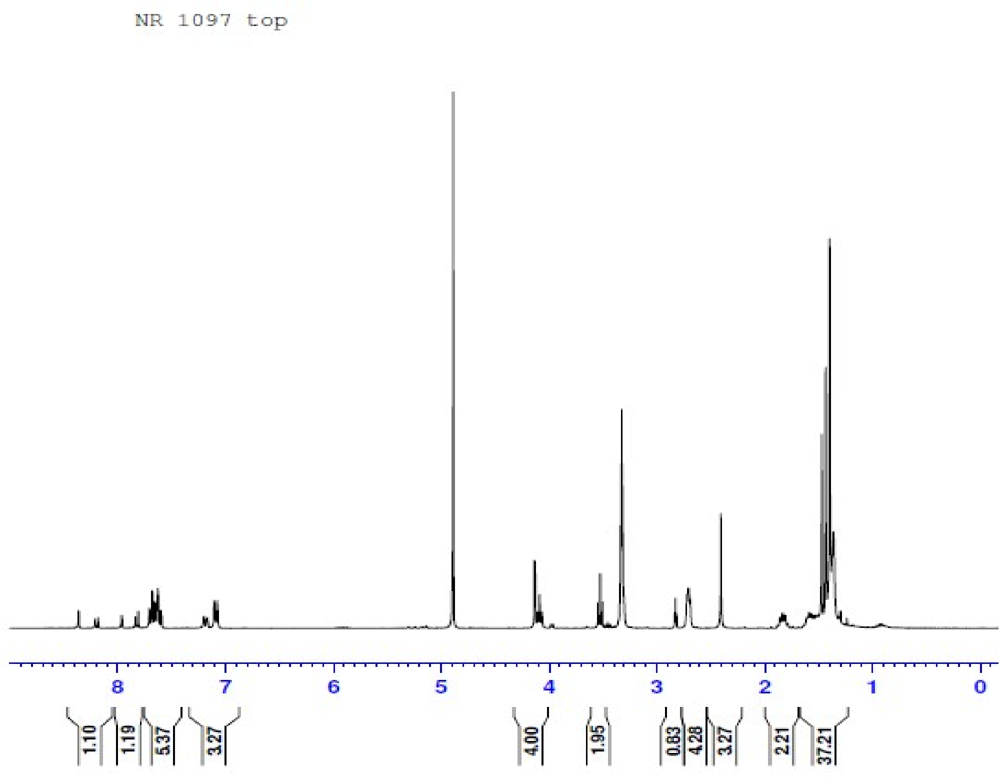

^{1}H NMR sprectrum of **7b** (rotamer I)

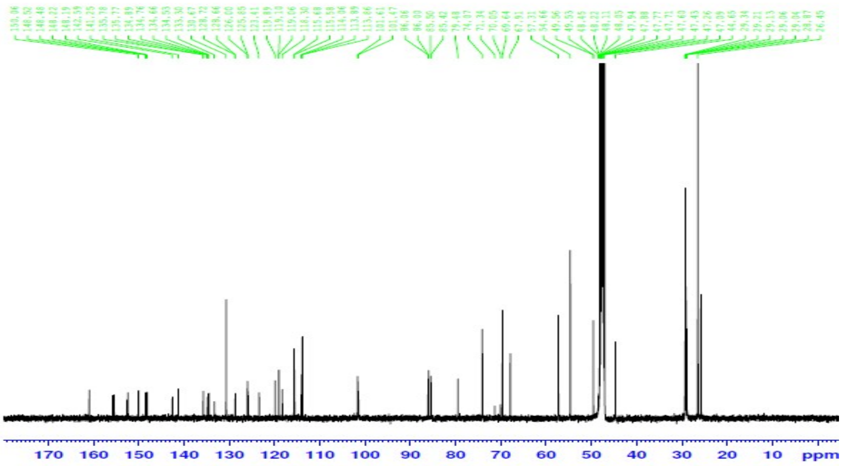

^{13}C NMR spectrum of **7b** (rotamer I)

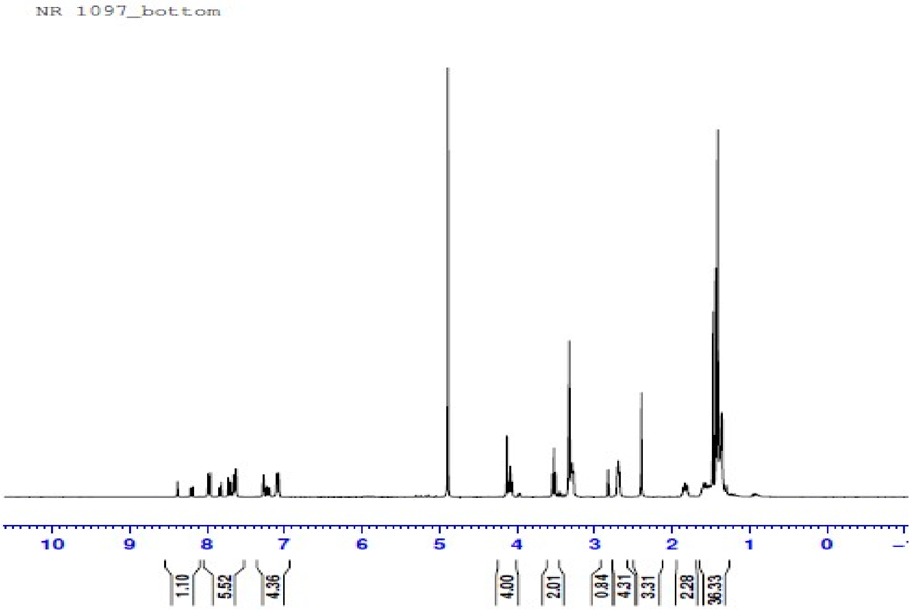

^{1}H NMR spectrum of **7b** (rotamer II).

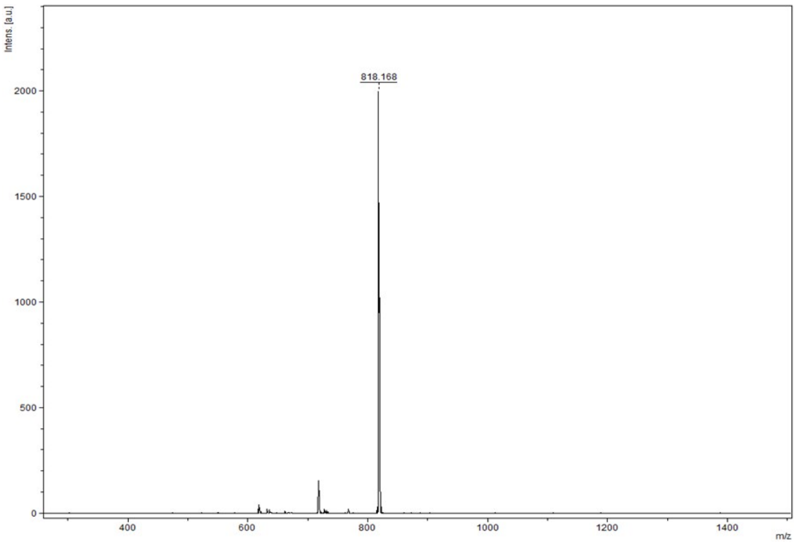

MALDI-TOF spectrum of **7b** (rotamer I).

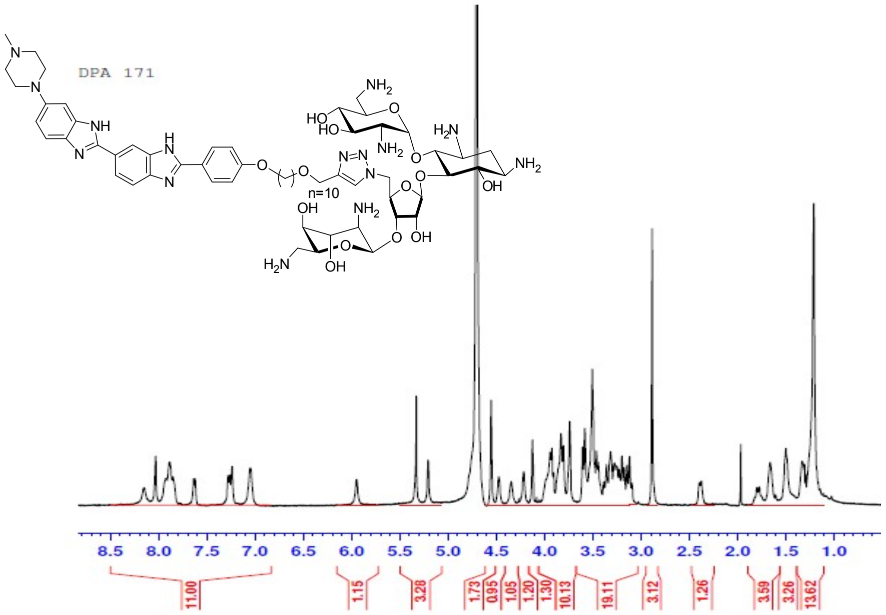

^{1}H NMR sprectrum of **7**.

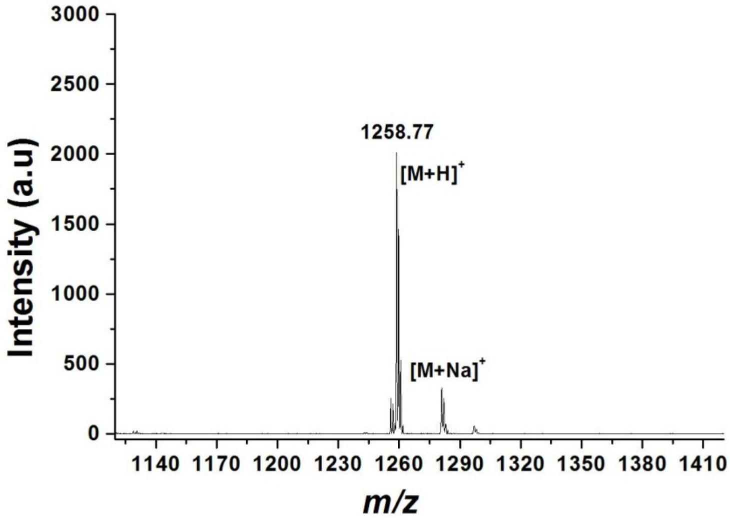

MALDI-TOF spectrum of **7**.

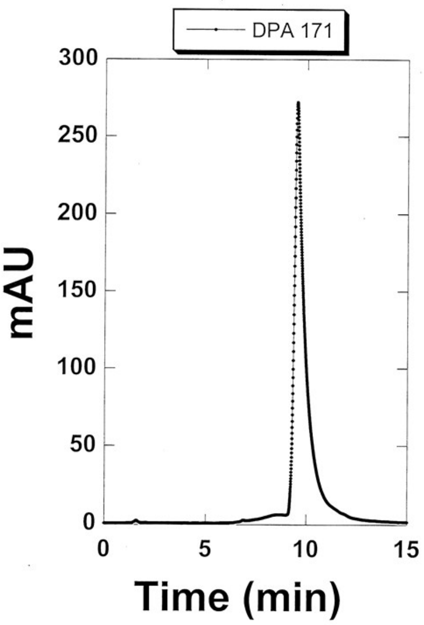

HPLC spectrum of **7**.

<u>HRMS analysis report of compound **1-7**</u>

Elemental Composition Report

Single Mass Analysis

Tolerance = 5.0 PPM / DBE: min = -1.5, max = 150.0
Element prediction: Off
Number of isotope peaks used for i-FIT = 2

Monoisotopic Mass, Even Electron Ions
95 formula(e) evaluated with 1 results within limits (up to 10 best isotopic matches for each mass)
Elements Used:
C: 0-100 H: 0-150 N: 14-16 O: 12-14

Minimum:				-1.5				
Maximum:		5.0	5.0	150.0				
Mass	Calc. Mass	mDa	PPM	DBE	i-FIT	Norm	Conf(%)	Formula
1100.5270	1100.5278	-0.8	-0.7	24.5	218.2	n/a	n/a	C51 H70 N15 O13

HRMS report of compound **1**.

Elemental Composition Report

Single Mass Analysis

Tolerance = 5.0 PPM / DBE: min = -1.5, max = 150.0
Element prediction: Off
Number of isotope peaks used for i-FIT = 2

Monoisotopic Mass, Even Electron Ions
99 formula(e) evaluated with 1 results within limits (up to 10 best isotopic matches for each mass)
Elements Used:
C: 0-100 H: 0-150 N: 14-16 O: 12-14

Minimum:				-1.5				
Maximum:		5.0	5.0	150.0				
Mass	Calc. Mass	mDa	PPM	DBE	i-FIT	Norm	Conf(%)	Formula
1144.5872	1144.5904	-3.2	-2.8	23.5	230.1	n/a	n/a	C54 H78 N15 O13

HRMS report of compound **2**.

Elemental Composition Report

Single Mass Analysis
Tolerance = 5.0 PPM / DBE: min = -1.5, max = 150.0
Element prediction: Off
Number of isotope peaks used for i-FIT = 2

Monoisotopic Mass, Even Electron Ions
107 formula(e) evaluated with 1 results within limits (up to 10 best isotopic matches for each mass)
Elements Used:
C: 0-100 H: 0-150 N: 14-16 O: 12-14

Minimum:				-1.5				
Maximum:		5.0	5.0	150.0				
Mass	Calc. Mass	mDa	PPM	DBE	i-FIT	Norm	Conf(%)	Formula
1212.6531	1212.6530	0.1	0.1	24.5	172.5	n/a	n/a	C59 H86 N15 O13

HRMS report of compound **3**.

Elemental Composition Report

Single Mass Analysis
Tolerance = 5.0 PPM / DBE: min = -1.5, max = 150.0
Element prediction: Off
Number of isotope peaks used for i-FIT = 2

Monoisotopic Mass, Even Electron Ions
102 formula(e) evaluated with 1 results within limits (up to 10 best isotopic matches for each mass)
Elements Used:
C: 0-100 H: 0-150 N: 14-16 O: 12-14

Minimum:				-1.5				
Maximum:		5.0	5.0	150.0				
Mass	Calc. Mass	mDa	PPM	DBE	i-FIT	Norm	Conf(%)	Formula
1172.5850	1172.5853	-0.3	-0.3	24.5	190.5	n/a	n/a	C55 H78 N15 O14

HRMS report of compound **4**.

Elemental Composition Report

Single Mass Analysis

Tolerance = 5.0 PPM / DBE: min = -1.5, max = 150.0
Element prediction: Off
Number of isotope peaks used for i-FIT = 2

Monoisotopic Mass, Even Electron Ions
105 formula(e) evaluated with 1 results within limits (up to 10 best isotopic matches for each mass)
Elements Used:
C: 0-100 H: 0-150 N: 14-16 O: 12-14

Mass	Calc. Mass	mDa	PPM	DBE	i-FIT	Norm	Conf(%)	Formula
Minimum:				-1.5				
Maximum:		5.0	5.0	150.0				
1200.6156	1200.6166	-1.0	-0.8	24.5	236.0	n/a	n/a	C57 H82 N15 O14

HRMS report of compound **5**.

Elemental Composition Report

Single Mass Analysis

Tolerance = 5.0 PPM / DBE: min = -1.5, max = 150.0
Element prediction: Off
Number of isotope peaks used for i-FIT = 2

Monoisotopic Mass, Even Electron Ions
107 formula(e) evaluated with 1 results within limits (up to 10 best isotopic matches for each mass)
Elements Used:
C: 0-100 H: 0-150 N: 14-16 O: 12-14

Mass	Calc. Mass	mDa	PPM	DBE	i-FIT	Norm	Conf(%)	Formula
Minimum:				-1.5				
Maximum:		5.0	5.0	150.0				
1230.6615	1230.6635	-2.0	-1.6	23.5	117.8	n/a	n/a	C59 H88 N15 O14

HRMS report of compound **6**.

Elemental Composition Report

Single Mass Analysis

Tolerance = 5.0 PPM / DBE: min = -1.5, max = 150.0
Element prediction: Off
Number of isotope peaks used for i-FIT = 2

Monoisotopic Mass, Even Electron Ions
111 formula(e) evaluated with 1 results within limits (up to 10 best isotopic matches for each mass)
Elements Used:
C: 0-100 H: 0-150 N: 14-16 O: 12-14

Minimum:				-1.5				
Maximum:		5.0	5.0	150.0				

Mass	Calc. Mass	mDa	PPM	DBE	i-FIT	Norm	Conf(%)	Formula
1256.6792	1256.6792	0.0	0.0	24.5	155.7	n/a	n/a	C61 H90 N15 O14

HRMS report of compound **7**.

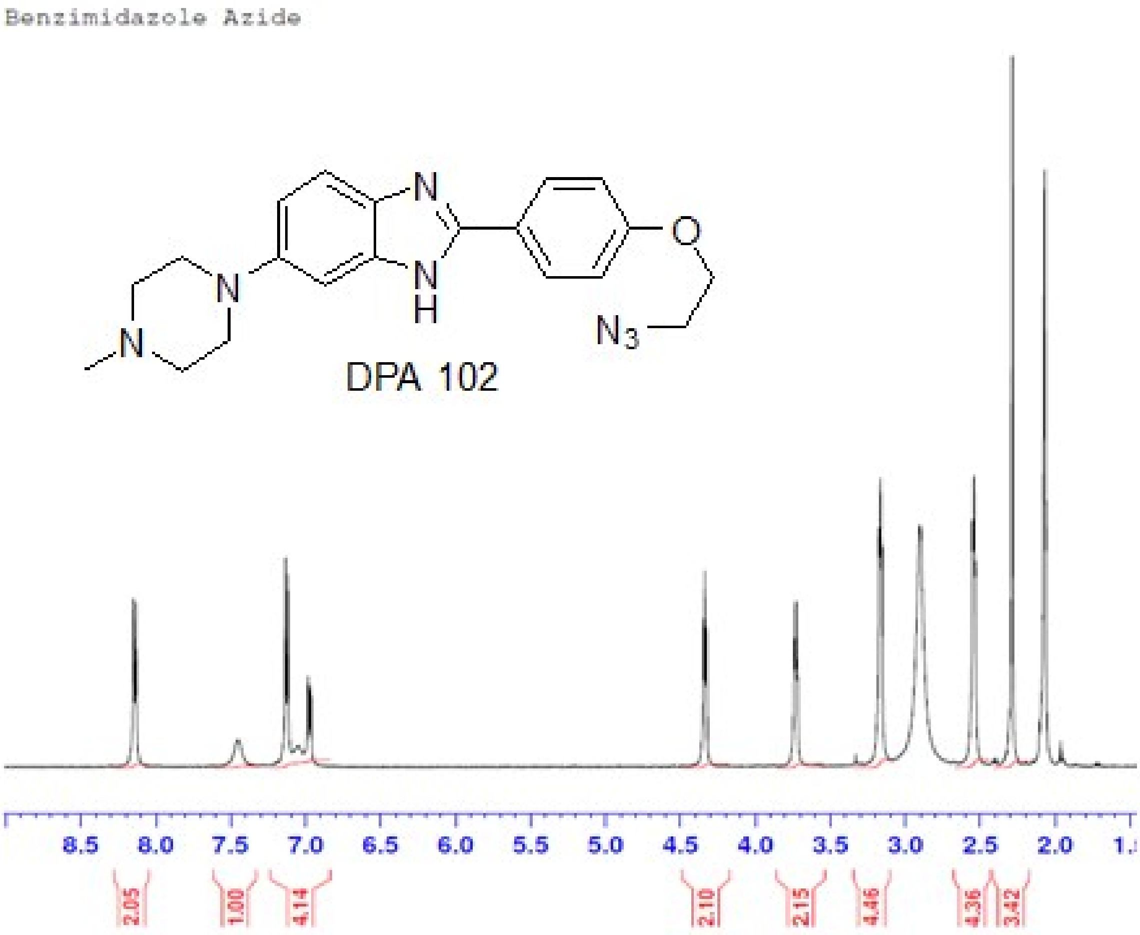

^{1}H NMR spectrum of compound **16**.

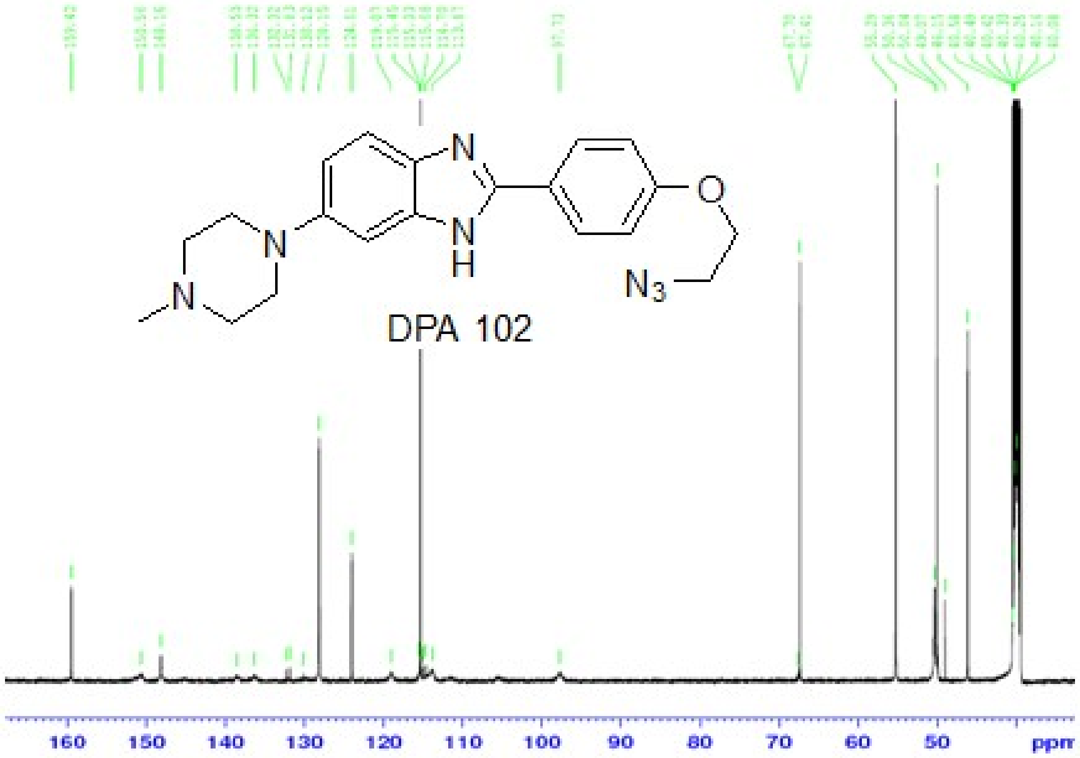

^{13}C NMR spectrum of compound **16**.

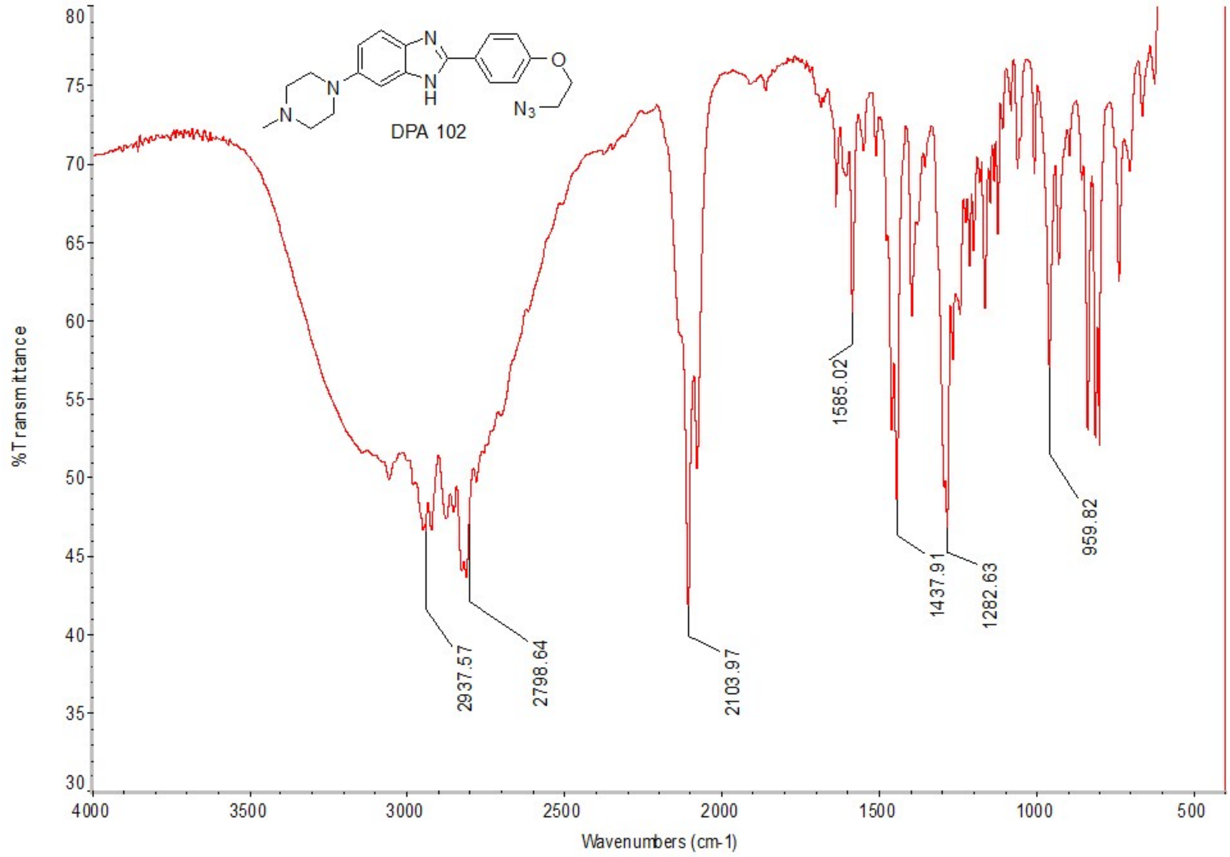

IR spectrum of compound **16**.

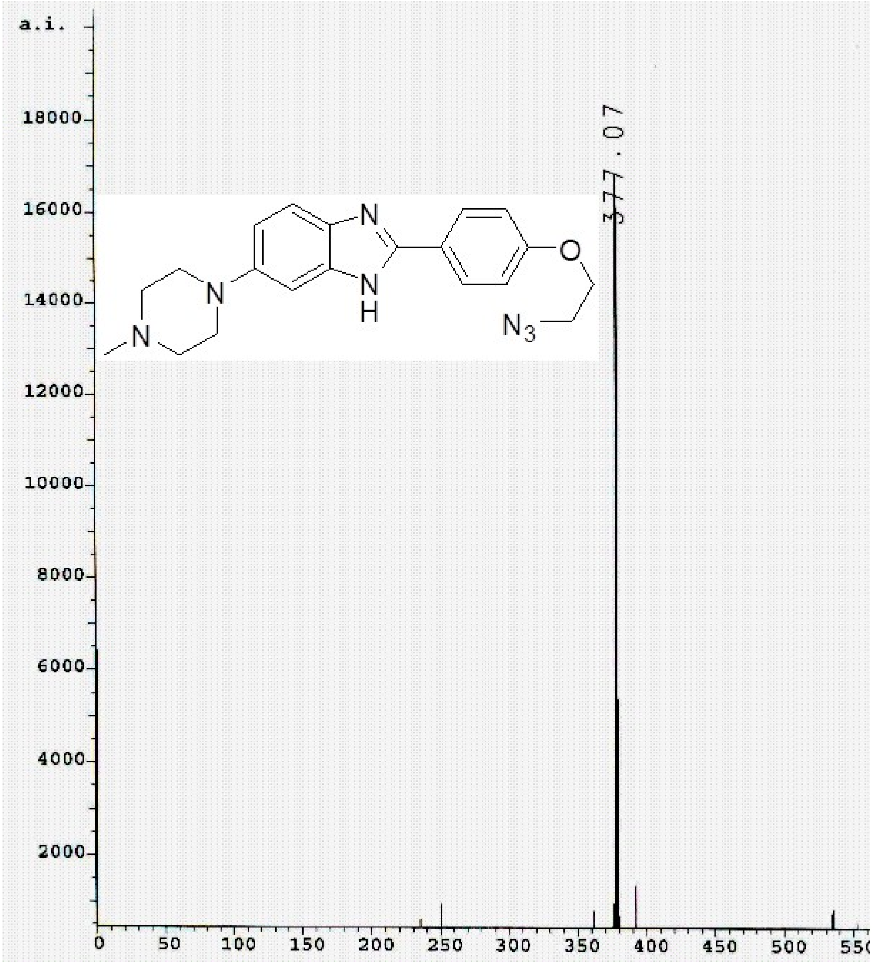

MALDI-TOF spectrum of **16**.

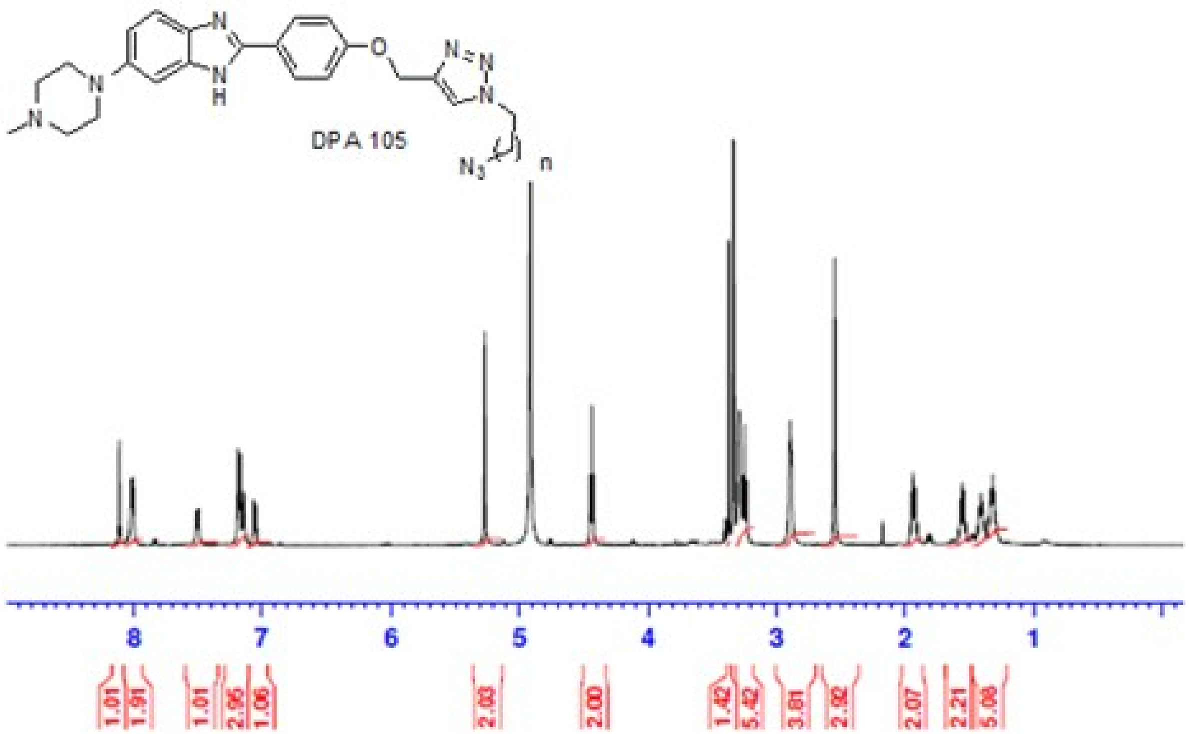

^{1}H NMR spectrum of **17**.

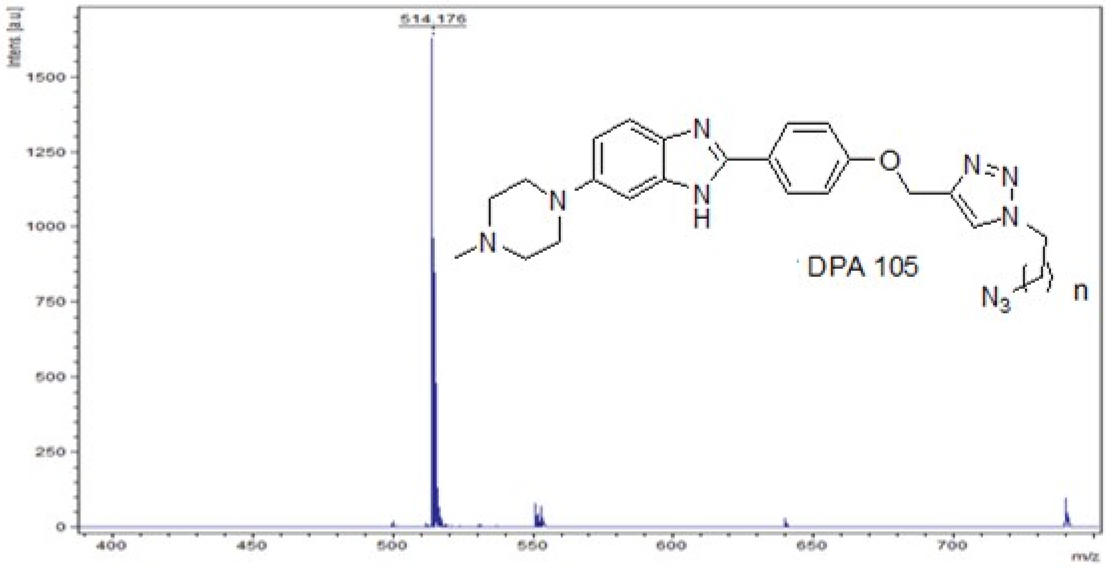

MALDI-TOF spectrum of **17**.

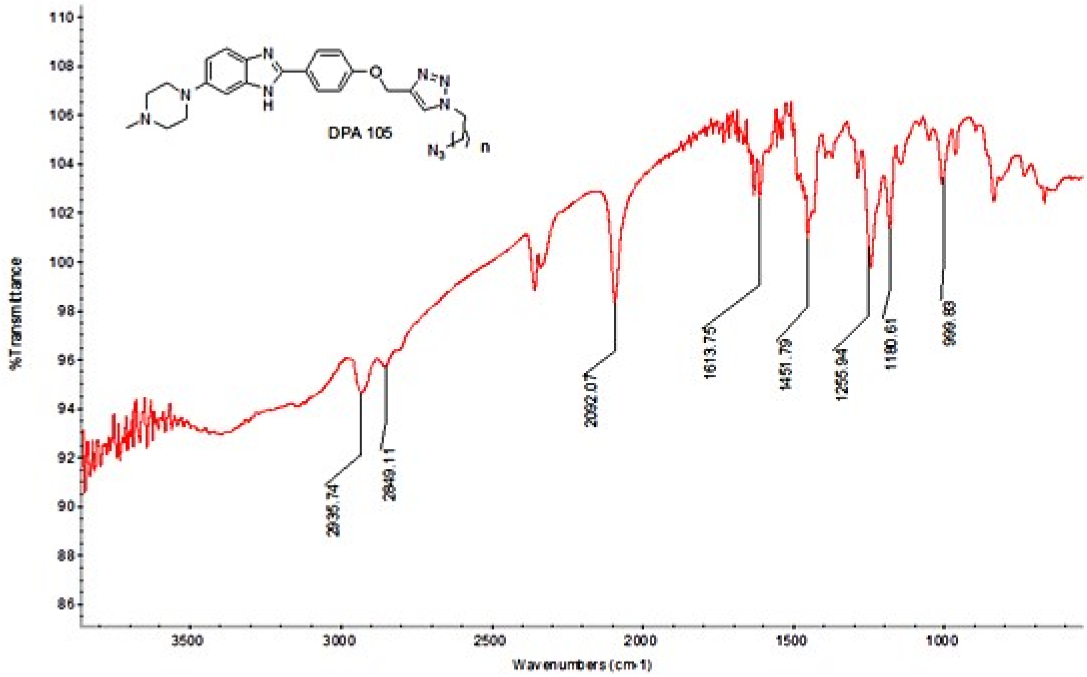

IR spectrum of **17**.

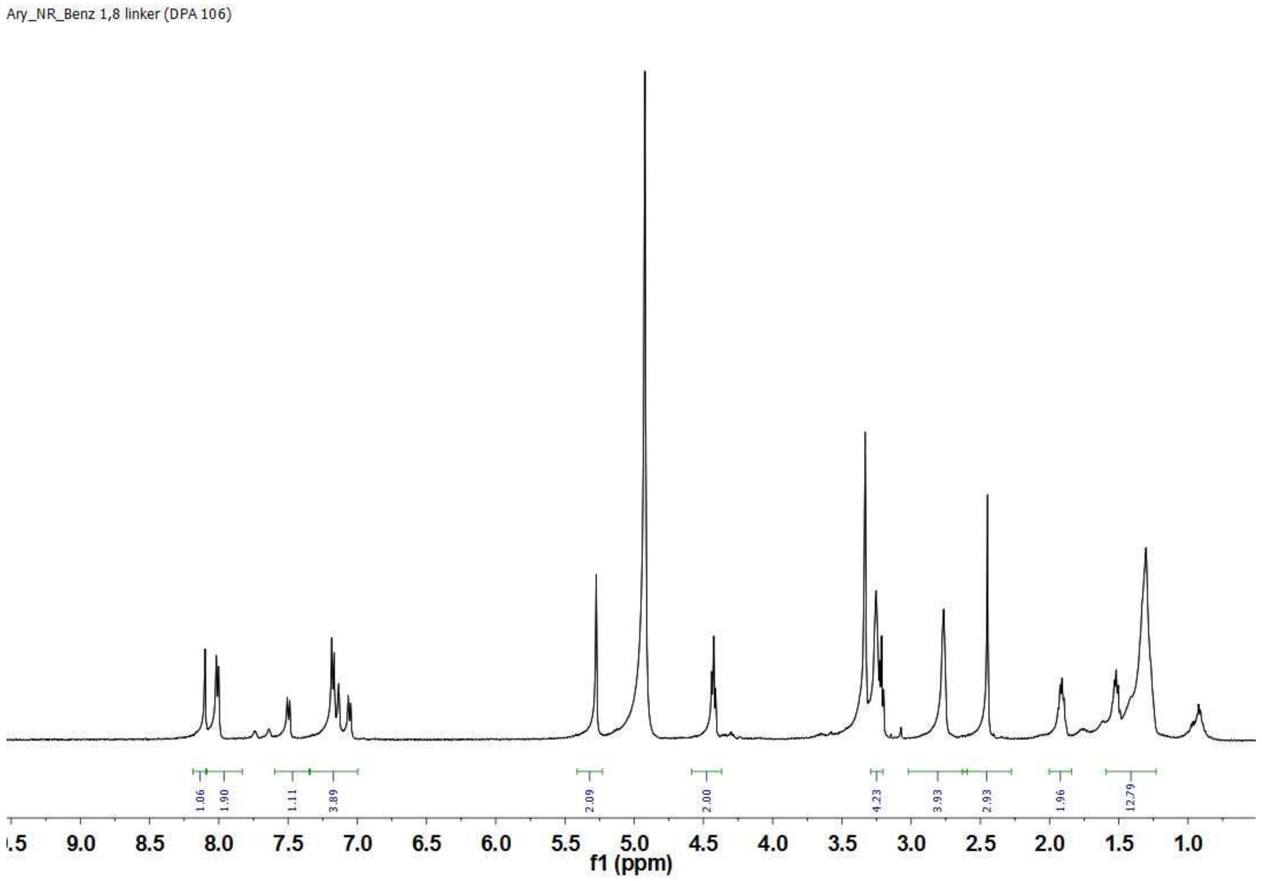

^{1}H NMR spectrum of compound **18**.

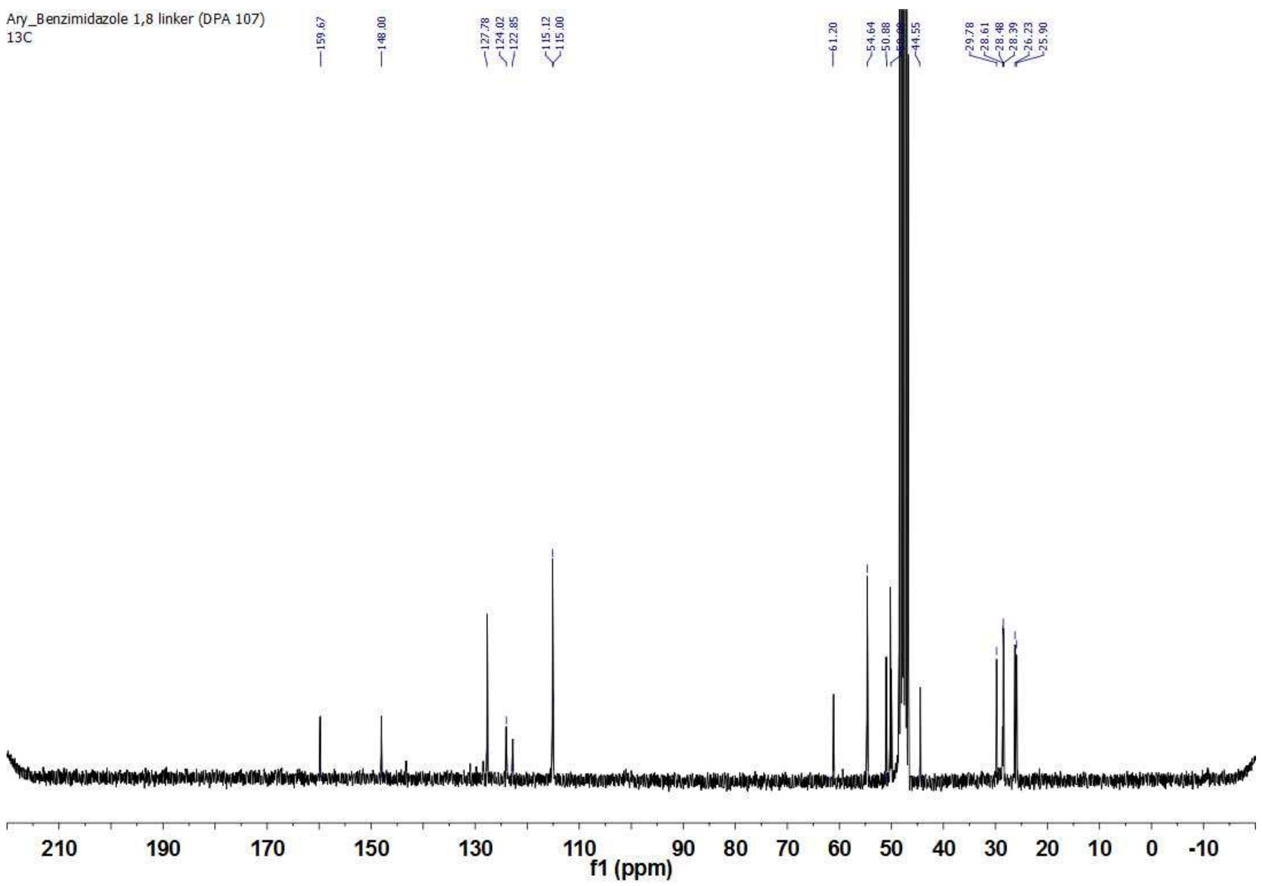

^{13}C NMR spectrum of compound **18.**

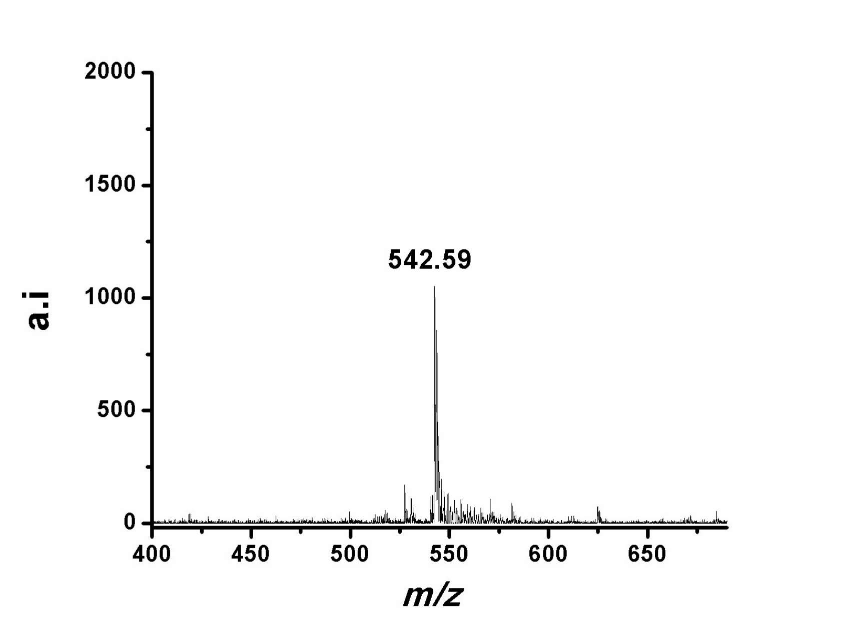

MALDI-TOF spectrum of compound **18**.

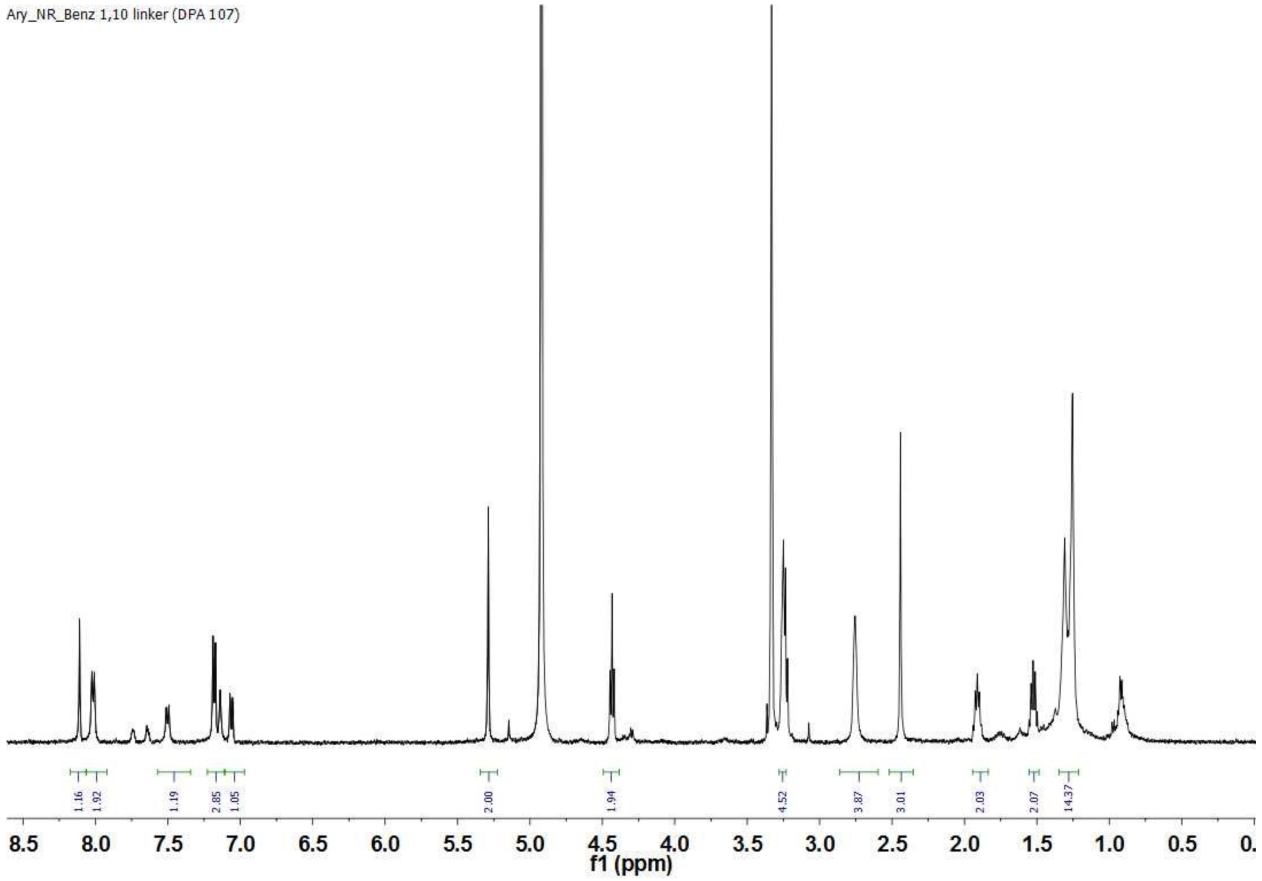

^{1}H NMR spectrum of compound **19**.

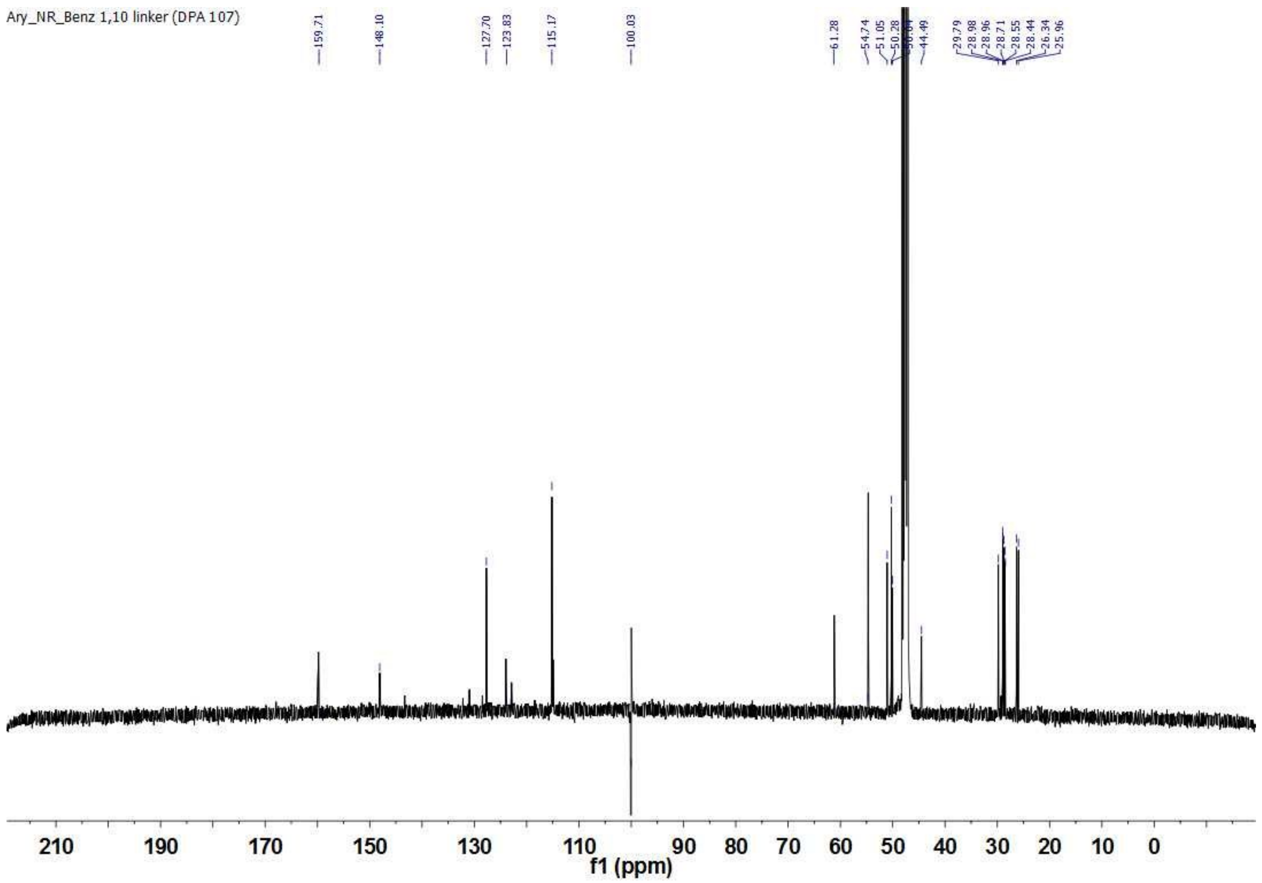

^{13}C NMR spectrum of compound **19.**

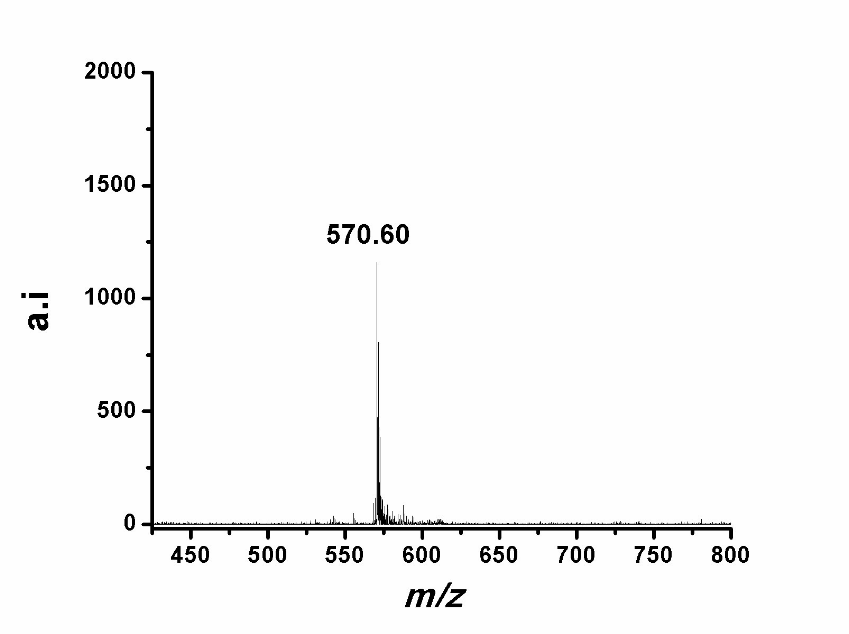

MALDI-TOF spectrum of compound **19**.

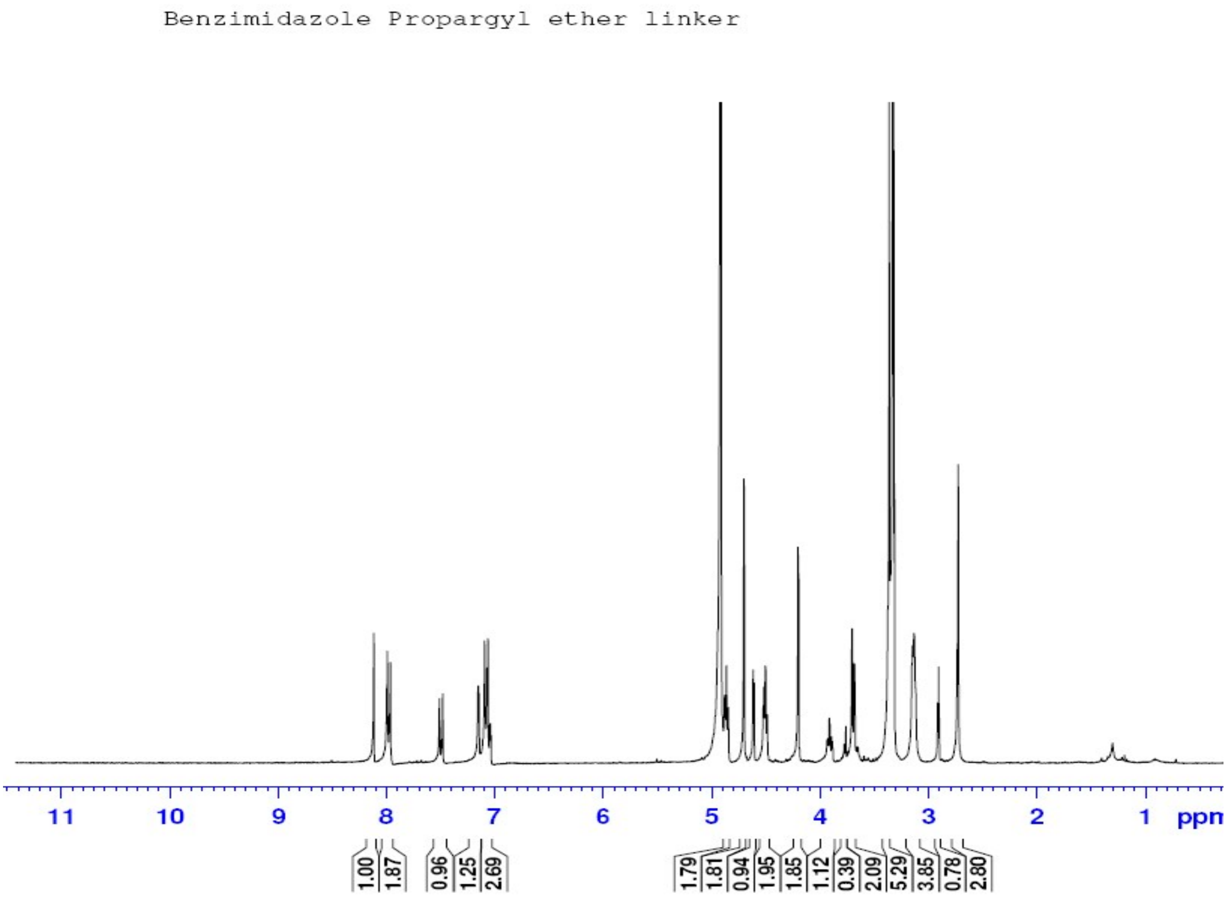

^{1}H NMR spectrum of compound **20**.

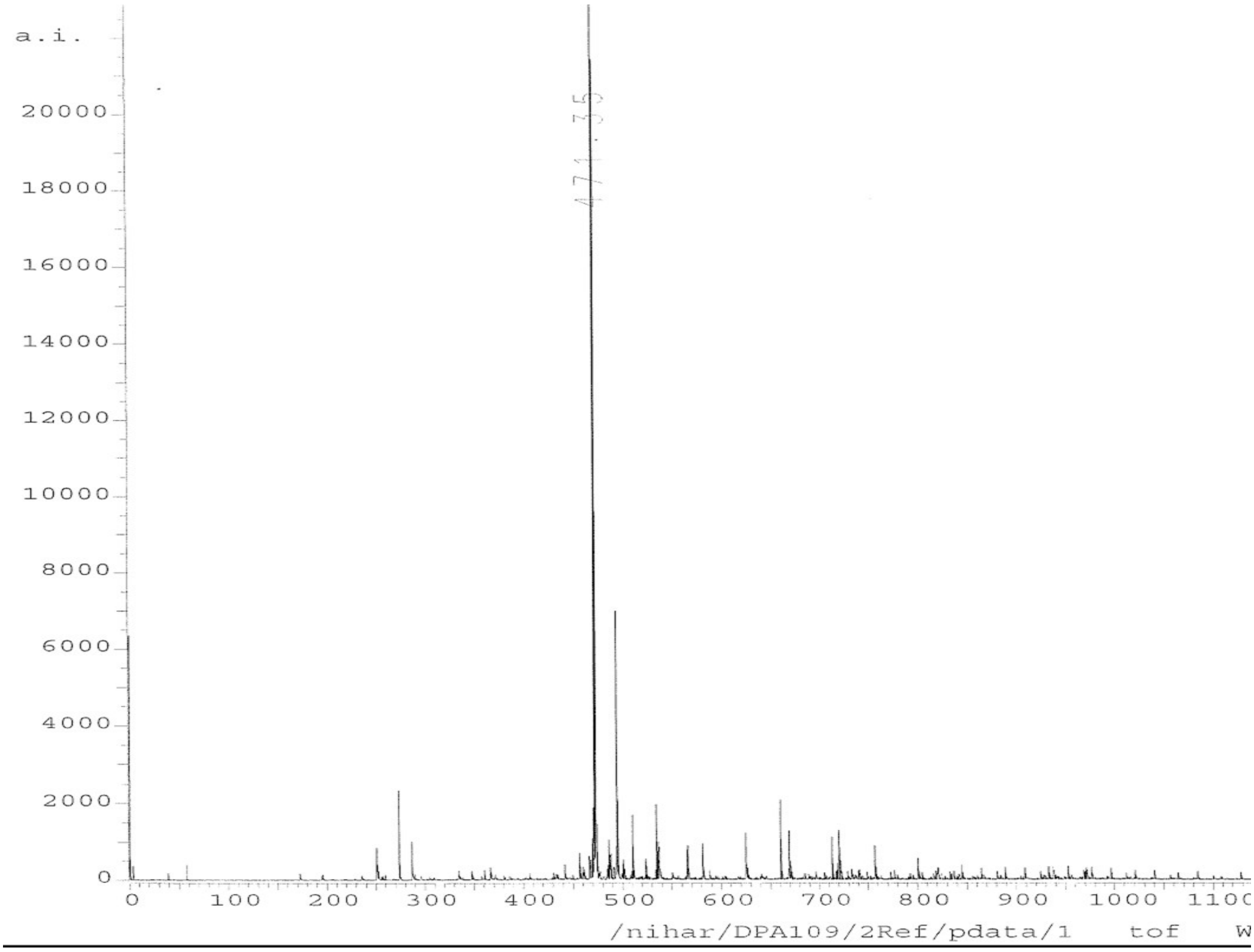

MALDI-TOF spectrum of compound **20**.

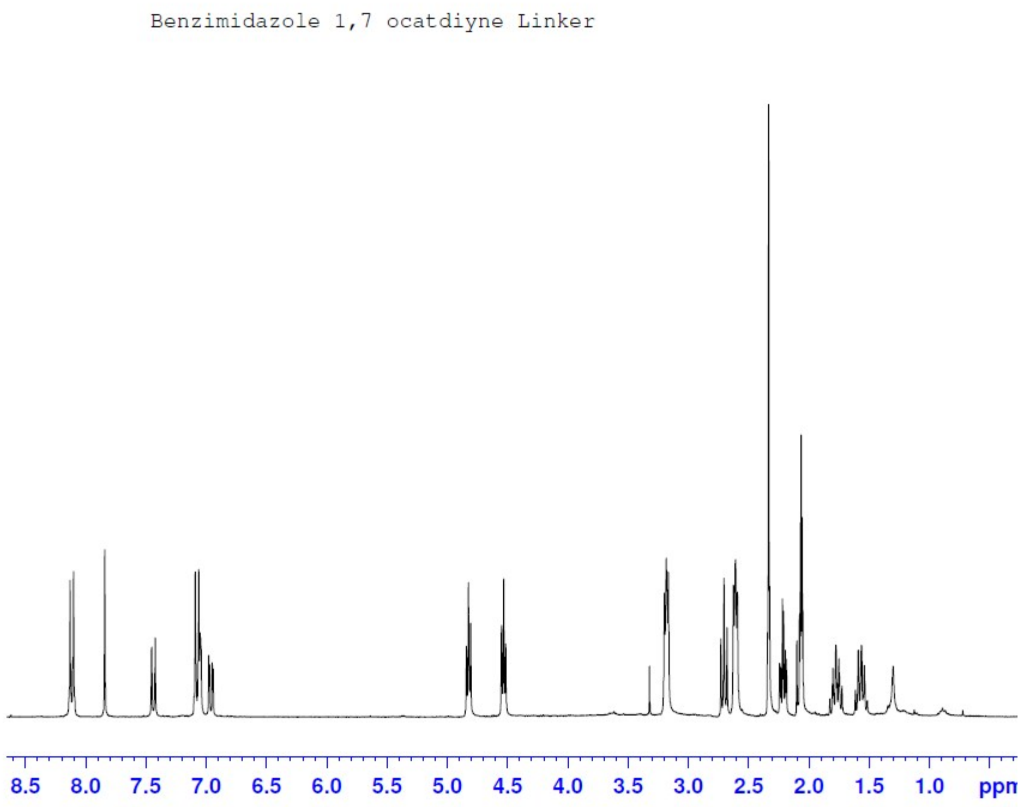

^{1}H NMR spectrum of compound **21**.

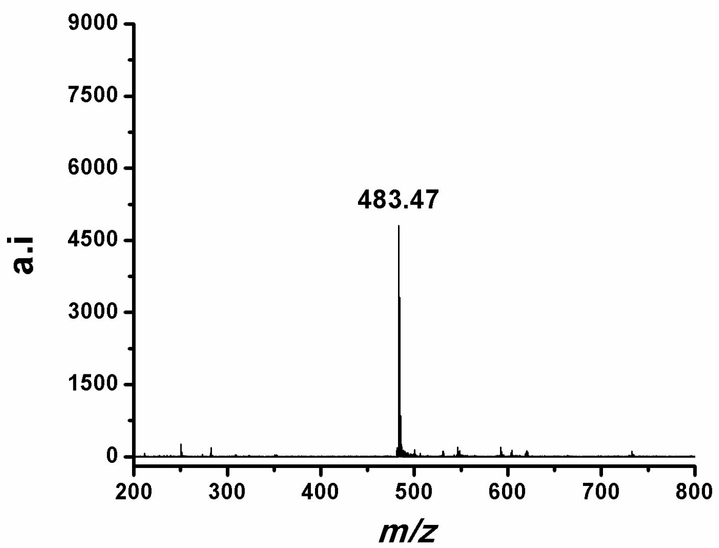

MALDI-TOF spectrum of compound **21**.

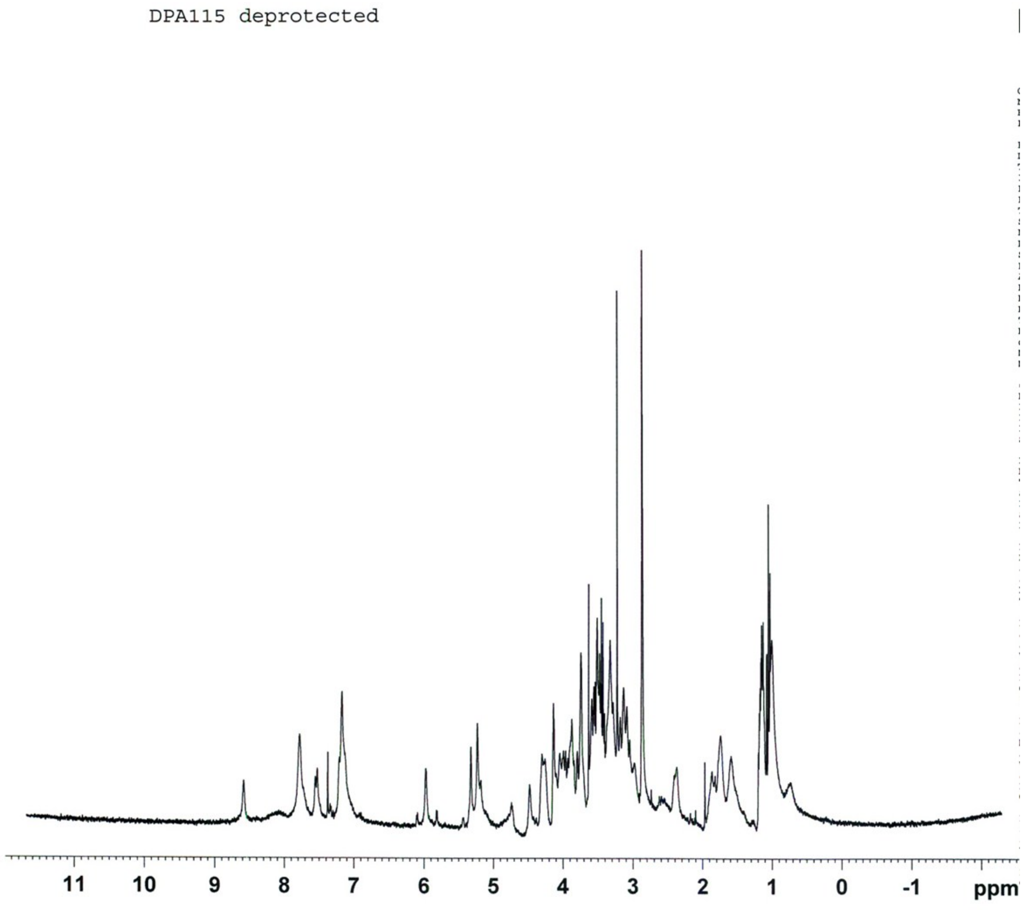

^{1}H NMR spectrum of **8**.

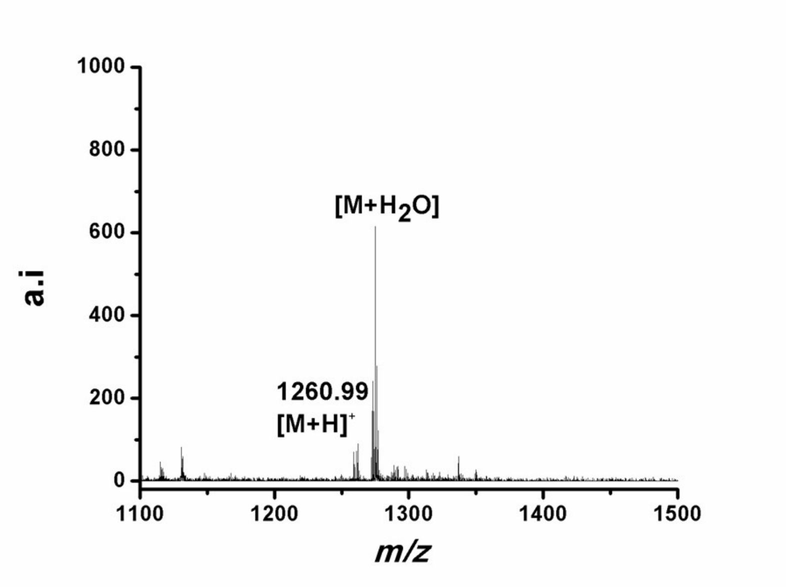

MALDI-TOF spectrum of **8**.

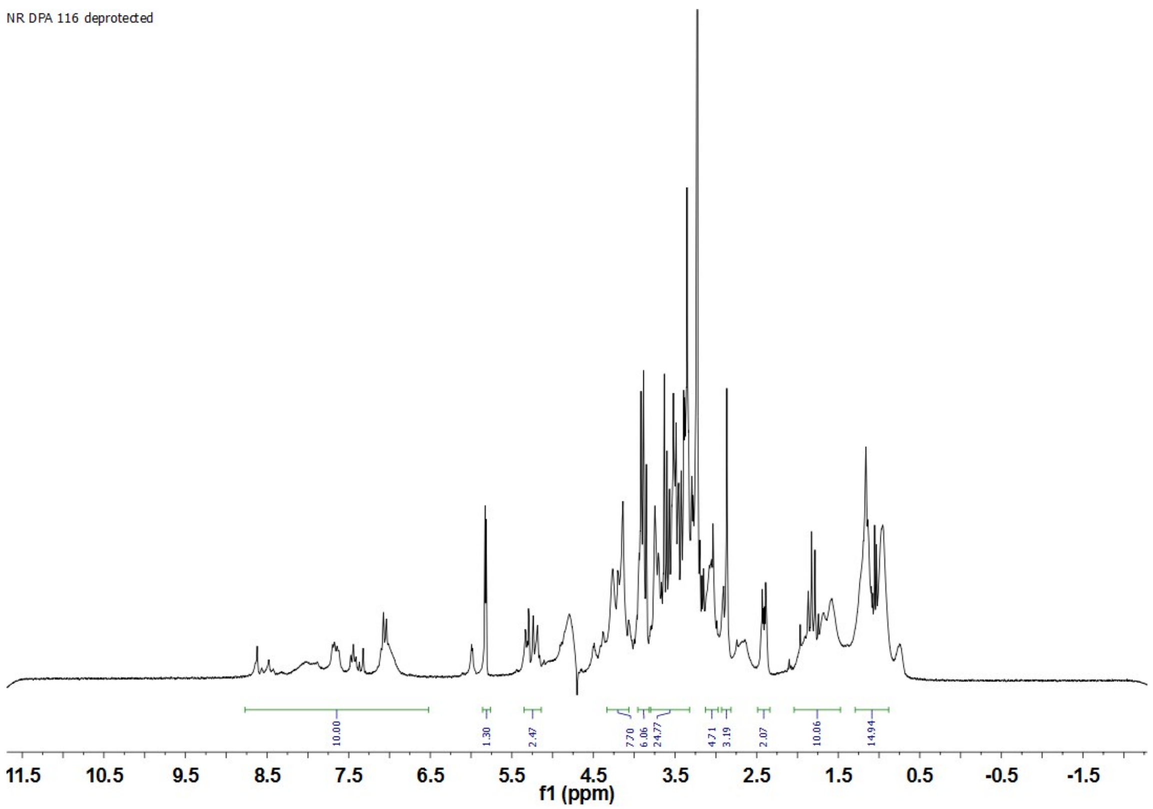

^{1}H NMR spectrum of **9**.

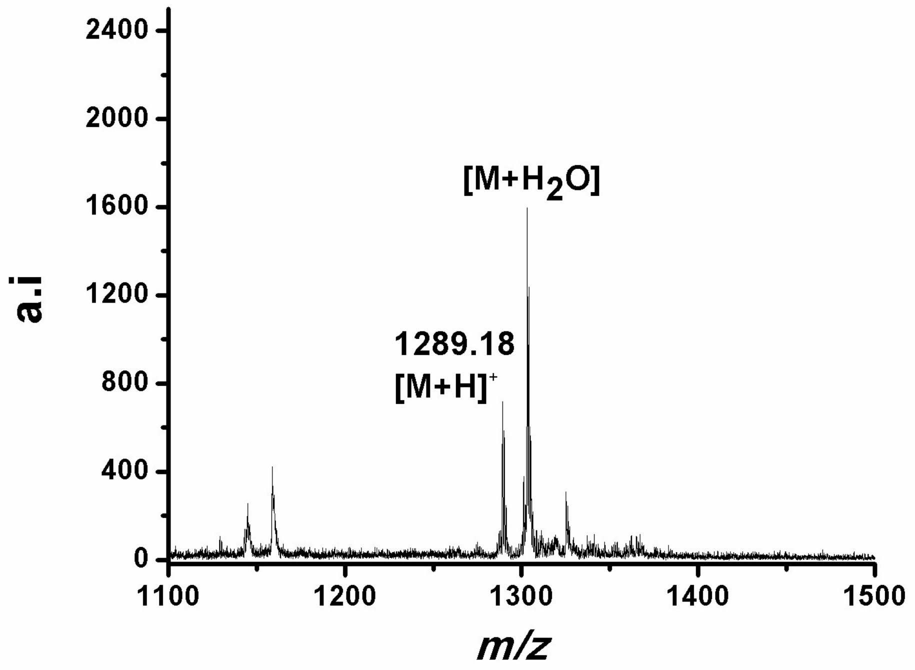

MALDI-TOF spectrum of **9**.

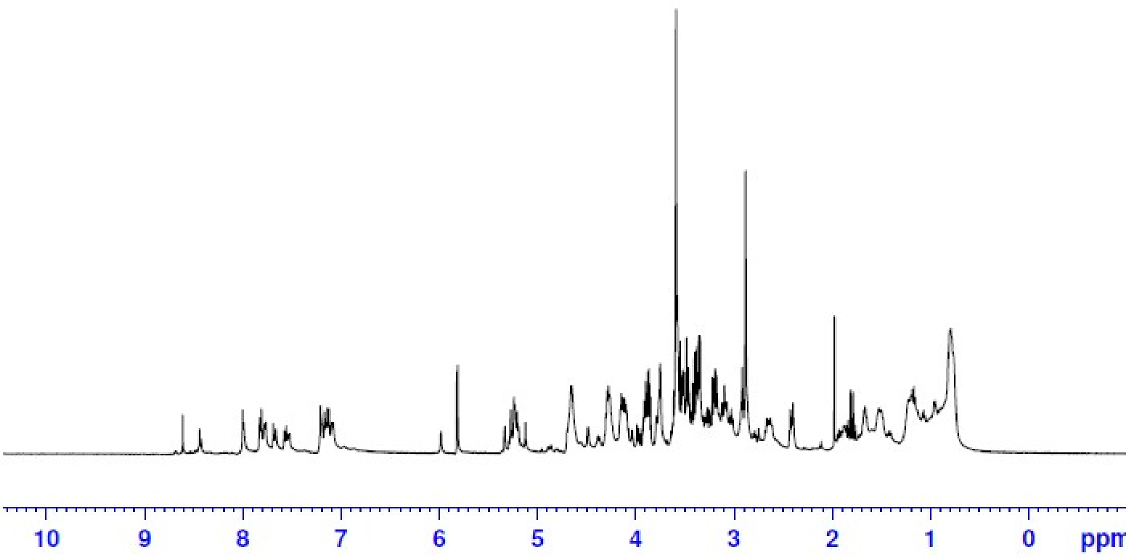

^{1}H NMR spectrum of **10**.

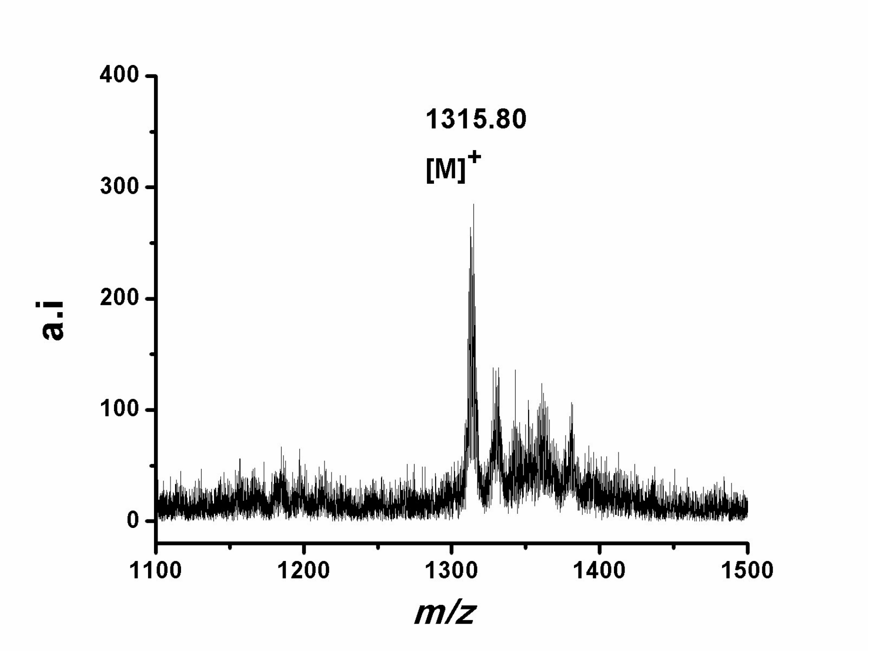

MALDI-TOF spectrum of **10**.

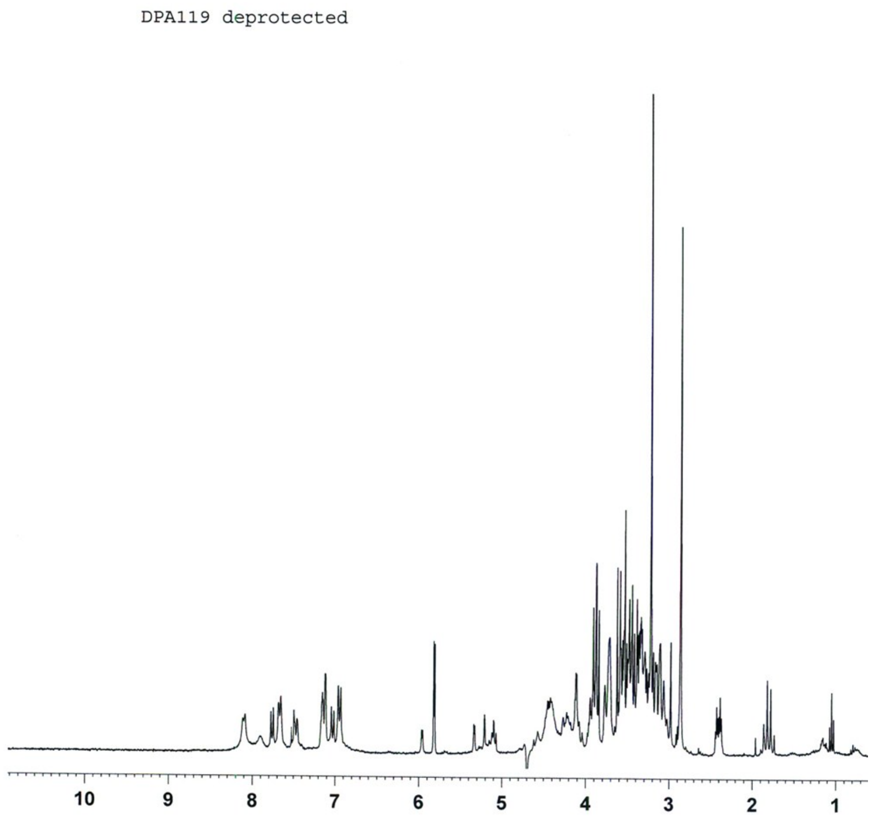

^{1}H NMR spectrum of **11**.

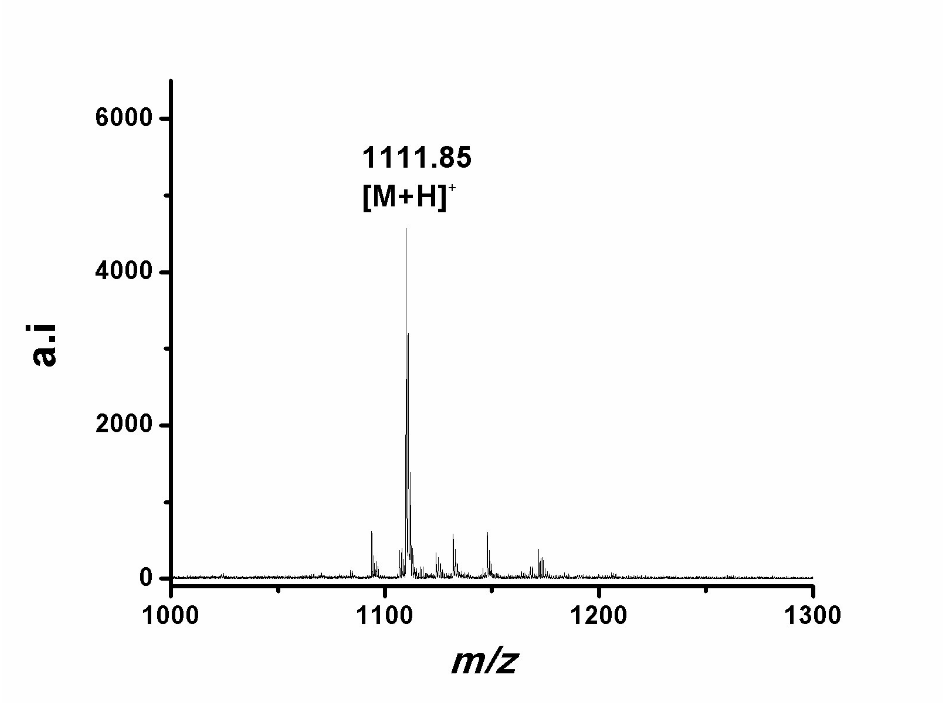

MALDI-TOF spectrum of **11**.

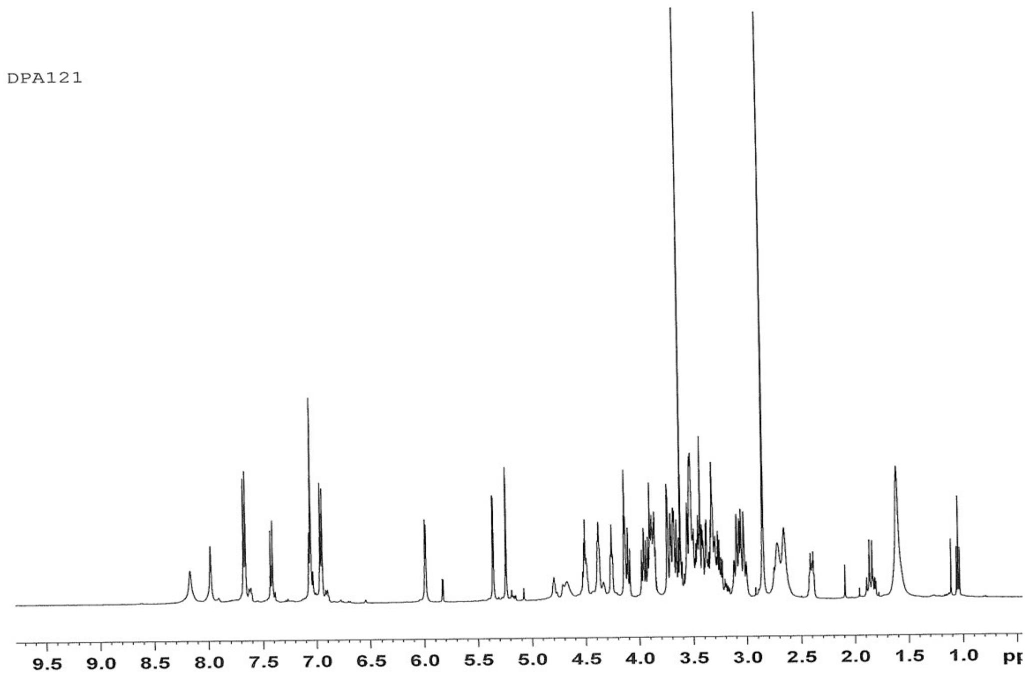

^{1}H NMR spectrum of **12**.

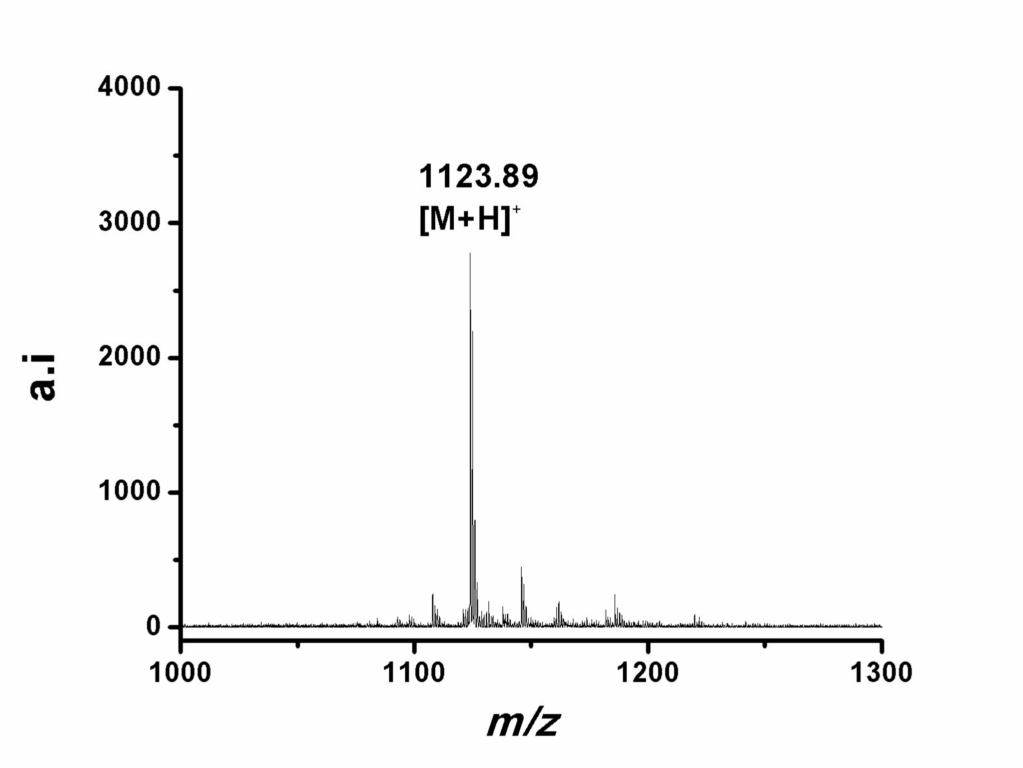

MALDI-TOF spectra of **12**

References

1. Ranjan, N.and Kumar, S.and Watkins, D.and Wang, D.and Appella, D. H., Arya, D. P., *Bioorg Med Chem Lett,* 2013, *20,* pp. 5689-5693.

2. Zhang, X.and Xiao, Y., Qian, X., *Org.Lett.,* 2008, *10,* pp. 29-32.

3. Kelly, D. P.and Bateman, S. A.and Hook, R. J.and Martin, R. F.and Reum, M. E.and Rose, M., Whittaker, A. R. D., *Aust.J.Chem.,* 1994, *47,* pp. 1751-1769.
